# Additive pharmacological interaction between sirtuin inhibitor cambinol and paclitaxel in MCF7 luminal and MDA-MB-231 triple-negative breast cancer cells

**DOI:** 10.1007/s43440-022-00393-w

**Published:** 2022-07-28

**Authors:** Anna Wawruszak, Estera Okon, Ilona Telejko, Arkadiusz Czerwonka, Jarogniew Luszczki

**Affiliations:** 1 Department of Biochemistry and Molecular Biology, Medical University of Lublin, Lublin, Poland; 2 Department of Pathophysiology, Medical University of Lublin, Lublin, Poland

**Keywords:** Breast cancer, Histone deacetylase inhibitor, Sirtuin inhibitor, Cambinol, Paclitaxel, Anticancer drugs

## Abstract

**Background:**

Breast cancer (BC) is the most common malignancy and the leading cause of cancer-related death in women worldwide. Sirtuin inhibitors (SIRTi), belonging to the histone deacetylase inhibitors group (HDIs), are potent epigenetic drugs that have been investigated for therapeutic use in different clinical disorders, including hematological malignancies and solid tumors.

**Methods:**

The influence of cambinol (CAM; SIRTi) used individually or in combination with standard chemotherapeutic paclitaxel (PAX) on viability (MTT assay), proliferation (BrdU assay), induction of apoptosis and cell cycle arrest (FACS analysis) was determined in MCF7 luminal and MDA-MB-231 triple-negative breast cancer (TNBC) cells. The types of pharmacological drug–drug interaction between CAM and PAX were determined by an exact and rigorous pharmacodynamic method—an isobolography, to determine the presence of synergism, addition or antagonism between analyzed drugs using a variety of fixed-dose ratios.

**Results:**

The combination of CAM and PAX at a fixed ratio of 1:1 exerted additive interaction in the viability of MCF7 and MDA-MB-231 BC cells. Both active agents used separately reduced viability and proliferation of BC cells as well as induced apoptosis and cell cycle arrest. These effects were much more evident in MCF7 than in MDA-MB-231 BC cells. Additionally, CAM combined with PAX increased anti-cancer activity compared to PAX used alone.

**Conclusion:**

CAM might be considered a potential therapeutic agent individually or in combined therapy with PAX against luminal or TNBC.

**Graphical abstract:**

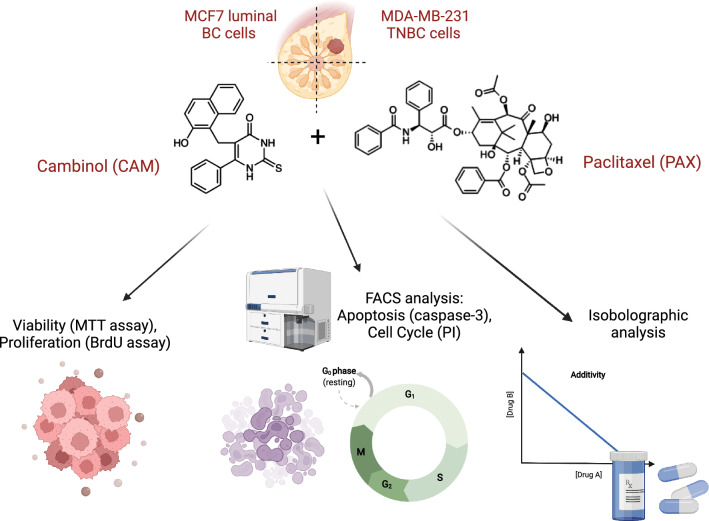

**Supplementary Information:**

The online version contains supplementary material available at 10.1007/s43440-022-00393-w.

## Introduction

Breast cancer (BC) was a leading cause of morbidity and mortality in women worldwide in 2021 [1]. Nowadays, BC is still the foremost cause of cancer-related deaths in women. Moreover, the incidence and mortality rates are expected to increase significantly in the following years. Despite significant progress in BC research and therapy, BC remains a serious clinical problem and represents a top oncological research priority. The standard therapeutic options depend on the subtype of BC and contain surgery, radiotherapy, chemotherapy, hormone therapy and immunotherapy [2].

The estrogen receptor-positive (ER+) subtype of BC accounts for approximately 75% of all BC cases. Endocrine therapy using aromatase inhibitors, selective ER modulators and ER-down-regulators provide appreciable clinical benefits through the reduction of BC recurrence and mortality. Anyhow, resistance to endocrine therapies is a severe obstacle limiting the effectiveness of ER+ BC treatment [3]. In turn, the most aggressive subtype of BC—triple-negative breast cancer (TNBC) accounts for 10–20% of all cases. Due to the lack of hormone receptors expression, hormone therapy is mainly ineffective for TNBC. Nonetheless, TNBC responds very well to traditional chemotherapy, which constitutes the most often recommended type of treatment [4].

Paclitaxel (PAX) is a tetracyclic diterpenoid that was first isolated from the bark of the Pacific yew tree. PAX, as high efficient and broad-spectrum natural anti-cancer drug, has been widely used in the therapy of ovarian, uterine, testis and other cancers [5]. PAX is also a frontline chemotherapy drug in BC treatment, especially in advanced metastatic cancer and TNBC. Unfortunately, resistance to PAX often impedes clinical management and adversely affects patient outcomes. To minimize or eliminate PAX resistance, diterpenoid is combined with other natural or synthetic chemotherapeutic agents [6].

New beneficial, targeted therapies combined with PAX are investigated to achieve better clinical outcomes in patients with BC [4]. Epigenetic abnormalities have emerged as an important hallmark of cancer development and progression. Given that histone deacetylases (HDACs) are essential to chromatin remodeling, their inhibitors have become promising anti-cancer drugs [7]. Histone deacetylase inhibitors (HDIs) are a relatively new class of anti-neoplastic agents that plays a vital role in the epigenetic and non-epigenetic regulation in cancer, including cell death, apoptosis or cell cycle arrest in cancer cells [7]. A balance in the activity of opposing enzymes: histone acetyltransferases (HATs) and histone deacetylases (HDACs), is indispensable in the epigenetic regulation of gene expression. Impairment in the balance between HATs and HDACs has been reported in the development of BC. Through the targeting of histone as well as non-histone proteins, HDIs maintain the cellular acetylation profile and reverse the function of several proteins responsible for BC development [8].

Sirtuins (SIRTs) belong to the 3rd class of HDACs. SIRTs require NAD+ as a cofactor and include SIRT1-7 proteins in mammals [9]. Cambinol (CAM) is a cell-permeable β-naphthol derivative that inhibits the activity of SIRT1 and SIRT2 [10]. It has been demonstrated that CAM enhances the cell response to PAX treatment in Burkitt lymphoma xenografts [10, 11]. All these findings suggest that SIRTs have a pivotal role in facilitating adverse effects of standard chemotherapeutics through reduction of their doses in combinatorial therapy [10].

According to our knowledge, there are no studies regarding the effect of CAM and PAX treatment in BC. Therefore, our present study aimed to investigate the anti-cancer activity of CAM individually or in combination with PAX to establish if this kind of treatment could enhance its anti-proliferative and pro-apoptotic activity in MCF7 luminal and MDA-MB-231 TNBC cells. Types of pharmacological interactions between CAM and PAX were determined using the advanced pharmacokinetic isobolographic method.

## Materials and methods

### Cell lines and culture conditions

The human MCF7 luminal and MDA-MB-231 TNBC cells (American Type Culture Collection; ATTC; Manassas, VA, USA) were incubated in Dulbecco's modified Eagle's medium (DMEM)/HAM’s F12 (Sigma, CA, USA) supplemented with 10% fetal bovine serum (FBS), penicillin (100 IU/mL) and streptomycin (100 µg/mL) (Sigma). Cultures were kept at 37 °C in a humidified atmosphere of 95% air and 5% CO_2_.

### Drug treatment

PAX (Sigma) and CAM (Sigma) were dissolved in dimethyl sulfoxide (DMSO) (Sigma) at 1 mM and 10 mM, respectively. To obtain the final drugs’ concentrations, stock solutions were diluted in the DMEM/HAM’s F12 culture medium.

### Cell viability assessment

Cell viability was measured using the MTT (4,5-dimethylthiazol-2-yl)-2,5-diphenyltetrazolium bromide) method, based on the ability of the mitochondria of viable cells to reduce yellow tetrazolium salt (MTT) to purple formazan crystals. MCF7 and MDA-MB-231 BC cells (1 × 10^4^ cells/ml) were incubated with PAX (0.001–1 µM) and CAM (0.01–0.1 mM) individually or in combination for 96 h. Following, BC cells were incubated with MTT solution at 5 mg/ml for 3 h. The absorbance was measured at 570 nm with an Infinite M200 Pro microplate reader (Tecan, Männedorf, Switzerland) after adding sodium dodecyl sulfate (SDS) buffer (10% SDS in 0.01 N HCl) overnight.

### Isobolographic analysis of PAX/CAM pharmacological interactions

Classification of pharmacodynamic interactions of PAX with CAM was performed by means of the type I isobolographic analysis for non-parallel concentration–effect curves in two BC cell lines, as described in detail earlier [12, 13]. In this study, the log-probit method allowed the determination of concentration–effect curves for PAX and CAM, when administered either alone or in combination at the fixed ratio of 1:1. The % of the inhibition of BC cell viability was transformed to probit (in two MCF7 and MDA-MB-231 cell lines measured by the MTT assay). After determining the median inhibitory concentrations (IC_50s_) for PAX and CAM, the test of parallelism between concentration–effect curves of PAX and CAM was performed as described in detail earlier [13]. Since the concentration–effect curves for PAX and CAM were non-parallel to each other, we calculated two IC_50_ theoretically calculated as additive (IC_50 add_) values for lower and upper isoboles of additivity, as presented earlier [14–16]. Additivity was present if the IC_50 mix_ values are placed close to or within the area bounded by the lower and upper isoboles of additivity [17].

### Cell proliferation assay (ELISA BrdU)

Cell proliferation was evaluated with Cell Proliferation Elisa, BrdU Kit (Roche, Germany). Optimized amounts of MCF7 and MDA-MB-231 BC cells (1 × 10^4^ cells/ml) were placed on a 96-well plate (Nunc, Rochester, NY, US) and treated with PAX and CAM (1/2 IC_50_ and IC_50_) individually or in combination for 48 h, followed by 10 µL/well BrdU Labeling Solution (100 µM) which was added, and cells were reincubated for an additional 24 h at 37 °C. Then, the culture medium was removed and cells were fixed in FixDenat solution (200 µL/well) (30 min, room temperature (RT)). The solution of anti-BrdU antibody coupled with horseradish peroxidase was subsequently added (100 µL/well) for 90 min at RT, and detected using tetramethylbenzidine substrate (TMB) (100 µL/well). Finally, 1 M sulfuric acid was added (25 µL/well) to stop the enzymatic reaction, and quantitation was performed spectrophotometrically at 450 nm using an Infinite M200 Pro microplate reader (Tecan).

### Detection of apoptosis

The assay was performed using PE Active Caspase-3 Apoptosis Kit (BD Pharmingen, San Diego, CA, USA). The BC cells were seeded at 1 × 10^5^/ml on 6-well plates (Nunc) and treated with PAX and CAM alone or in combination (PAX/CAM) for 48 h. Then, cells were washed with phosphate-buffered saline (PBS), fixed and permeabilized using the Cytofix/Cytoperm Solution according to the manufacturer’s protocol. Finally, cells were washed twice in the Perm/Wash Buffer before intracellular staining with PE-conjugated anti-active caspase-3 monoclonal rabbit antibodies. Labeled cells were analyzed by FACSCalibur (BD Biosciences), operating with CellQuest software.

### The cell cycle assessment

The cell cycle assessment was performed with the FACS Calibur™ Flow Cytometer (BD Biosciences) equipped with an argon-ion laser (488 nm). In the beginning, BC cells were fixed with 70% ethanol at − 20 °C. After that step, the cells were stained with propidium iodide (PI) utilizing PI/RNase Staining Buffer (BD Biosciences), according to the manufacturer's protocol. The course of the cell cycle was determined by a non-commercial flow cytometry analyzing software—WinMDI 2.9 (facs.scripps.edu/software.html) and Cylchred Version 1.0.2 (University of Wales, UK). 10,000 events were recorded and an acquisition rate was 60 events/second.

### Statistical analysis

Statistical analysis was performed using GraphPad Prism 5 software. One-way analysis of variance (ANOVA test) with Tukey’s post hoc testing was used for multiple comparisons. The normal distribution to justify parametric statistical methods was checked using the Shapiro–Wilk normality test. Results were statistically relevant if *p* < 0.05 (*p < 0.05, **p < 0.01, ***p < 0.001). All the IC_50_ values for PAX and CAM (when administered singly and in combination) were computed by means of the log-probit analysis. The Student’s *t* test with Welch correction was used to statistically compare the experimentally derived IC_50 mix_ values with their respective theoretical additive IC_50 add_ values, as presented elsewhere [13].

## Results

### Decrease of MCF7 luminal and MDA-MB-231 TNBC viability after CAM and PAX treatment administered individually or in combination

**Fig. 1:**
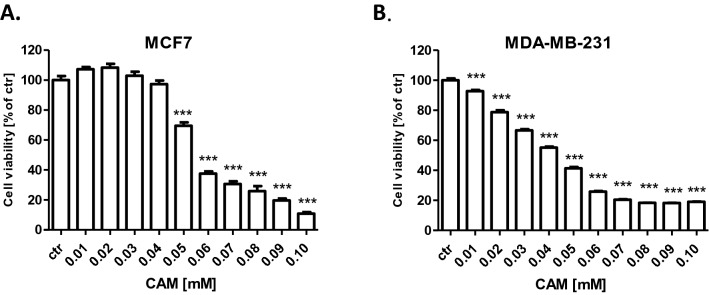
The effect of cambinol (CAM) (0.01–0.1 mM) on the viability of **A** MCF7 and **B** MDA-MB-231 human breast cancer (BC) cell lines was measured by (4,5-dimethylthiazol-2-yl)-2,5-diphenyltetrazolium bromide (MTT) assay after 96 h. Data are presented as mean ± standard deviation (± SD) of the mean, *n* = 18 per concentration from three independent experiments. One-way ANOVA, Tukey’s post hoc testing. *p < 0.05, **p < 0.01, ***p < 0.001

**Fig. 2:**
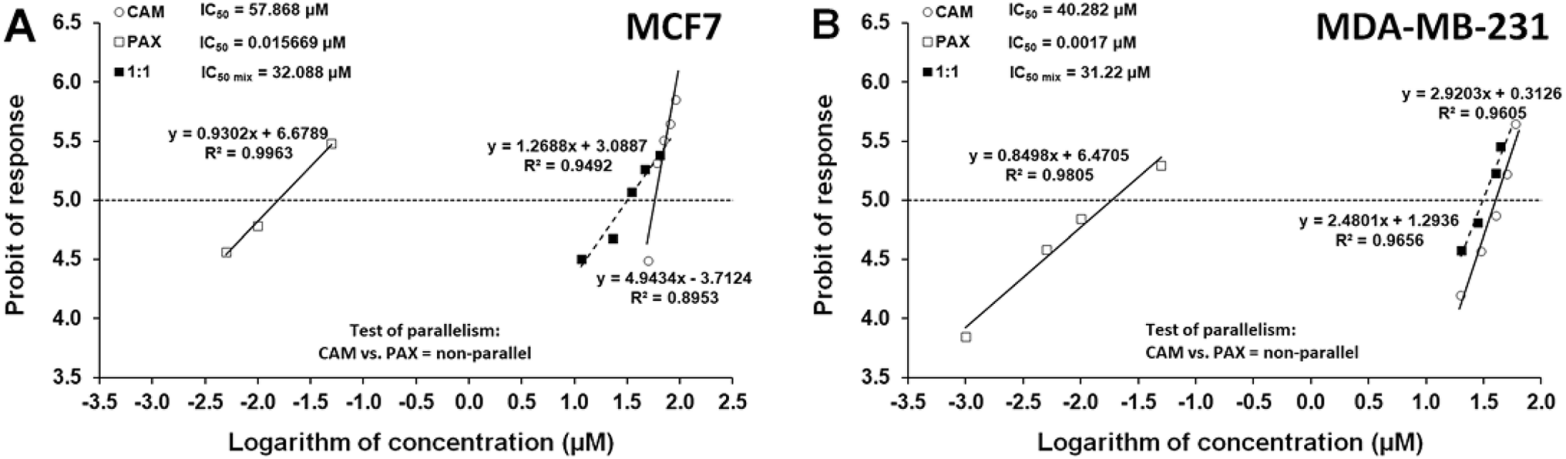
Concentration–effect relationship curves (CECs) for paclitaxel (PAX) and cambinol (CAM) when administered singly and combined in a fixed ratio of 1:1 for **A** MCF7 and **B** MDA-MB-231 cells. Concentrations of CAM and PAX were transformed into logarithms and the anti-proliferative effects into probits. Linearly related equations of CECs are presented on the graph. The dotted line reflecting the 5^th^ probit indicates the IC_50_ values for PAX and CAM. Test of parallelism between PAX and CAM indicated that both lines are not parallel to each other

**Fig. 3:**
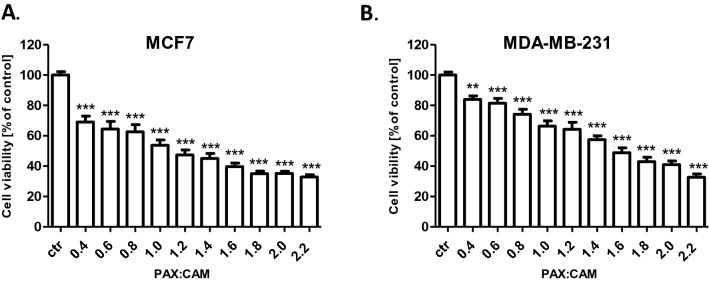
The effect of paclitaxel (PAX) and cambinol (CAM) (1:1) on the viability of **A** MCF7 and **B** MDA-MB-231 human breast cancer (BC) cell lines was measured by the (4,5-dimethylthiazol-2-yl)-2,5-diphenyltetrazolium bromide (MTT) test after 96 h incubation. Different ratios of the PAX and CAM IC_50_ (2.0 = IC_50_ + IC_50_) were used. Data are presented as mean ± standard deviation (± SD) of the mean, *n* = 18 per concentration from three independent experiments. One-way ANOVA, Tukey’s post hoc testing. **p < 0.01, ***p < 0.001

In this experiment we determined the cell growth inhibitory activity of CAM and PAX [18] using 3-(4,5-dimethylthiazol-2-yl)-2,5-diphenyltetrazolium bromide (MTT) assay. The influence of both active agents on the viability of MCF7 luminal and MDA-MB-231 TNBC cell lines was done to determine the IC_50_ values, which were calculated based on the log-probit analysis of the concentration–response relationship (CRR) effects and they were shown in Table 1. Analyzed BC cell lines were exposed to a clear culture medium (control) or increasing concentrations of CAM (0.01–0.1 mM) (Fig. 1) and PAX (0.001–1 µM) [18]. As shown in Fig. 1, CAM administered individually reduced cell viability in both investigated BC cell lines in a dose-dependent manner compared with control (untreated cells). The cytotoxic effect of CAM was weaker in MCF7 luminal BC cells (IC_50_ = 57.87 ± 3.48 µM; Fig. 2) (one-way ANOVA, number of groups 11, *F* = 313.4, *R* square 0.9477, *df* = 183 (total)) than in MDA-MB-231 TNBC cells (IC_50_ = 40.28 ± 4.10 µM; Fig. 2) (one-way ANOVA, number of groups 11, *F* = 1774, *R*^2^ = 0.9896, *df* = 197 (total)). Then, we analyzed the dose-related inhibitory effects of CAM and PAX on BC cells to subsequently find out whether a combination of CAM with PAX can enhance the anti-cancer activity of this agent. To determine the cytotoxic effect of PAX and CAM in combination, BC cells were incubated with a 1:1 drug mixture in increasing, different ratios of IC_50_ (2.0 = IC_50_ of PAX + IC_50_ of CAM). Similar to individual treatment, we have shown the concentration-dependent inhibition of BC cell viability in both analyzed BC cell lines (Fig. 3). As opposed to a single therapy, the MCF7 cell line was more sensitive to PAX/CAM combined treatment than MDA-MB-231 TNBC cells.

**Table 1:** Half-maximal inhibitory concentrations (IC_50_ ± SEM) for cambinol (CAM) and paclitaxel (PAX) [18] in MCF7 and MDA-MB-231 breast cancer (BC) cells

Cell line	Drug	IC_50_ (μM)	*N*	*S*	*f* ratio	Collateralism
MCF7	PAX	0.0157 ± 0.0065 [18]	72	7.460	2.615	NC
	CAM	57.87 ± 3.48	120			
MDA-MB-231	PAX	0.0017 ± 0.0005 [18]	72	6.828	1.998	NC
	CAM	40.28 ± 4.10	120			

The parallelism between the dose–response lines of PAX and CAM was assessed by means of the log-probit method as described earlier [13, 18]

*n* number of items, *S* slope function ratio, *f ratio* factor for slope function ratio, *NC* not collateral

### Log-probit concentration–response effects of CAM, PAX, and their combination (CAM + PAX) in the MCF7 and MDA-MB-231 cell lines

Both, CAM and PAX exerted, in a concentration-dependent manner, the anti-proliferative effect in the MTT assay in both, MCF7 (Fig. 3A), and MDA-MB-231 (Fig. 2B) cell lines, respectively. For CAM, the linearly related log-probit equation (*y* = 4.943*x* − 3.7124; *R*^2^ = 0.8953) allowed calculating the IC_50_ value, which was 57.868 µM in the MCF7 cell line (Fig. 2A; Table 1). For PAX, the log-probit equation (*y* = 0.9302*x* + 6.6789; *R*^2^ = 0.9963) allowed calculating the IC_50_ value, which amounted to 0.0157 µM in the MCF7 cell line (Fig. 2A; Table 1). Additionally, the combination of both drugs (CAM + PAX) at the fixed ratio of 1:1 produced also the anti-proliferative effect with log-probit equation (*y* = 1.2688*x* + 3.0887; *R*^2^ = 0.9492) and IC_50 mix_ value amounting to 32.088 µM in the MCF7 cell line (Fig. 2A; Table 2). Similarly, in the MDA-MB-231 cell line, the linearly related log-probit equation for CAM (*y* = 2.9203*x* + 0.3126; *R*^2^ = 0.9656) allowed calculating the IC_50_ value, which was 40.282 µM (Fig. 2B; Table 1). For PAX, the log-probit equation (*y* = 0.8498*x* + 6.4705; *R*^2^ = 0.9805) allowed calculating the IC_50_ value, which amounted to 0.0017 µM in the MDA-MB-231 cell line (Fig. 2B, Table 1). Additionally, the combination of both drugs (CAM + PAX) at the fixed ratio of 1:1 exerted the anti-proliferative effect with log-probit equation (*y* = 2.4801*x* + 1.2936; *R*^2^ = 0.9656) and IC_50 mix_ value amounting to 31.22 µM in the MDA-MB-231 cell line (Fig. 2B; Table 2). The test of parallelism between log-probit concentration–response lines for CAM and PAX revealed that both log-probit lines are not parallel to each other in the two tested cell lines (MCF7; Fig. 2A and MDA-MB-231; Fig. 2B).

**Table 2:** Isobolographic analysis of pharmacological drug–drug interactions between paclitaxel (PAX) and cambinol (CAM) in MCF7 and MDA-MB-231 breast cancer (BC) cells

Cell line	IC_50 mix_ (μM)	*n*_mix_	Lower IC_50 add_ (μM)	*n*_add_	Upper IC_50 add_ (μM)	Interaction
MCF7	32.09 ± 7.51	120	10.23 ± 5.59	188	47.69 ± 6.86	Additivity
MDA-MB-231	31.22 ± 4.18	96	13.19 ± 3.81	188	27.10 ± 4.02	Additivity

Results are IC_50_ values (± SEM) for the mixture of PAX and CAM determined experimentally (IC_50 mix_) and theoretically calculated as an additive (IC_50 add_), which inhibit proliferation in MCF7 and MDA-MB-231 BC lines, as measured in the MTT assay. The experimentally derived IC_50 mix_ values for the mixture of PAX with CAM were statistically compared with their respective theoretical additive IC_50 add_ values by the use of unpaired Student’s *t* test with Welch correction, according to Tallarida [16]

*n*_*mix*_ number of items for the experimental mixture, *n*_*add*_ number of items calculated for the additive mixture

### Type I isobolographic analysis of drug–drug pharmacological interaction between CAM and PAX in MCF7 and MDA-MB-231 BC cells

**Fig. 4:**
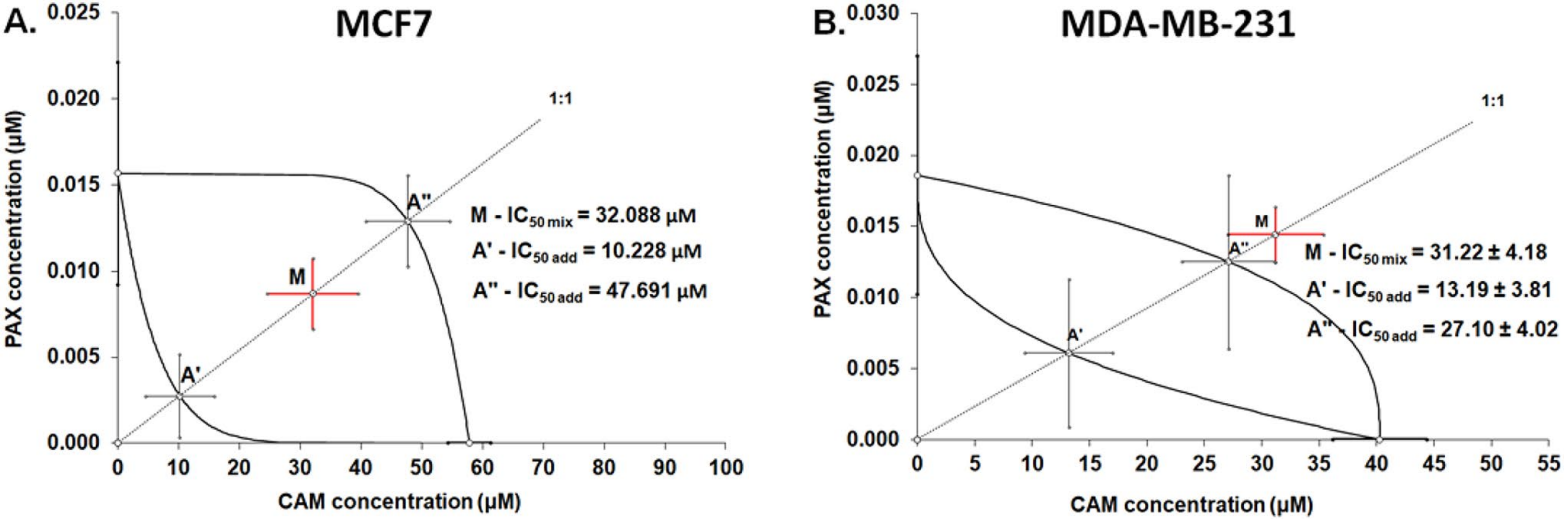
Isobolographic analysis of interactions between paclitaxel (PAX) and cambinol (CAM) in (A) MCF7 and (B) MDA-MB-231 breast cancer (BC) cells. Isobolograms display additive interactions between PAX and CAM with respect to their anti-proliferative effects in MCF7 and MDA-MB-231 cell lines measured in vitro by the (4,5-dimethylthiazol-2-yl)-2,5-diphenyltetrazolium bromide (MTT) assay. The IC_50_ values for CAM and PAX are plotted graphically on abscissa and ordinate, respectively. The lower and upper isoboles of additivity represent the curves connecting the IC_50_ values for PAX and CAM administered alone. The points *A*′ and *A*″ depict the theoretically calculated IC_50 add_ values. The point *M* represents the experimentally derived IC_50 mix_ value for the mixture of PAX and CAM that produced a 50% anti-proliferative effect (50% isobole) in two BC cell lines measured in vitro by the MTT assay

The isobolographic analysis of interaction revealed that the mixture of PAX with CAM at the fixed ratio of 1:1 exerted additive interaction in the MCF7 breast cancer cells (Fig. 4A). The experimentally determined IC_50 mix_ value was 32.088 µM and was placed on isobologram within the area bounded by the lower and upper isoboles of additivity (Fig. 4A). The theoretically calculated IC_50 add_ values, accepted as additive, were 10.228 µM for the lower and 47.691 µM for the upper isoboles, respectively (Fig. 4A). The experimental IC_50 mix_ value for this combination did not significantly differ from the computed IC_50 add_ values with Student’s *t* test with Welch correction (*t* = 1.534, *df* = 277.6, *p* = 0.1262, Table 2, Fig. 4A). Similarly, in the MDA-MB-231 cancer cell line, the mixture of PAX with CAM at the fixed ratio of 1:1 exerted additive interaction (Fig. 4B). The experimentally determined IC_50 mix_ value was 31.22 µM and was placed on an isobologram slightly above the area bounded by the lower and upper isoboles of additivity (Fig. 4B). The theoretically calculated IC_50 add_ values, accepted as an additive, were 13.19 µM for the lower and 27.10 µM for the upper isoboles, respectively (Fig. 4A). No statistical significance was observed between the IC_50 mix_ value for the combination of PAX with CAM and the IC_50 add_ values (with Student’s *t* test with Welch correction: *t* = 0.7114, *df* = 245.4, *p* = 0.4775, Table 2, Fig. 4B).

### Decrease the proliferation of MCF7 and MDA-MB-231 BC cells after CAM and PAX treatment administered singly and in combination

**Fig. 5:**
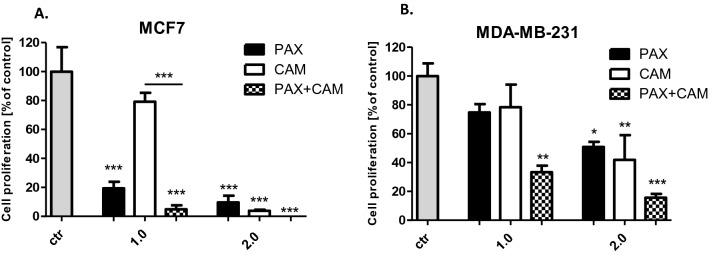
The effect of paclitaxel (PAX) and cambinol (CAM) on the proliferation of **A** MCF7 and **B** MDA-MB-231 breast cancer (BC) cells in the 5-bromo-2'-deoxyuridine (BrdU) assay. BC cells were incubated for 48 h individually (control) or with the drugs (1.0 = ½ IC_50_, 2.0 = IC_50_ determined in the (4,5-dimethylthiazol-2-yl)-2,5-diphenyltetrazolium bromide (MTT) assay). Data are presented as mean ± standard deviation (± SD) of the mean, *n* = 4 per concentration from two independent experiments. One-way ANOVA, Tukey’s post hoc testing. p* < 0.05, **p < 0.01, ***p < 0.001

Inhibition of BC cell proliferation after CAM and PAX treatment was determined by the ELISA BrdU assay. For this purpose, MCF7 luminal and MDA-MB-231 TNBC cells were treated with the culture medium (control) or PAX and CAM individually or in combination in a 1:1 ratio (1.0 = 1/2 IC_50_, 2.0 = IC_50_ determined in the MTT assay). In our study, we have demonstrated that both PAX and CAM administered alone reduced the proliferation of BC cells as evaluated by measuring BrdU incorporation into cellular DNA in proliferating cells. The stronger statistically significant anti-proliferative individual effect of PAX treatment was observed in MCF7 luminal BC cells (PAX_2.0_ = 9.665%) than in MDA-MB-231 TNBC cells (Fig. 5) (PAX_2.0_ = 50.88%) (with Student’s *t* test: *t* = 7.138, *df* = 6, *p* = 0.0004). The combination of PAX with CAM reduced BC cell proliferation in both analyzed BC cell lines. The effect of the combined PAX/CAM therapy was also more evident in MCF7 cells (1.0 decrease in cell proliferation to 4.8%) than in MDA-MB-231 BC cells (1.0 decrease in proliferation to 33.32%) (with Student’s *t* test: *t* = 5.323, *df* = 6, *p* = 0.018). However, the statistical significance was demonstrated only at a concentration of 1/2 IC_50_ (1.0) of both active agents, suggesting that CAM and PAX in this combination were much more effective than in individual, which confirms the additive nature of the pharmacological PAX–CAM interaction (Fig. 5).

### Induction of apoptosis in MCF7 and MDA-MB-231 BC cells after CAM and PAX treatment administered singly and in combination

**Fig. 6:**
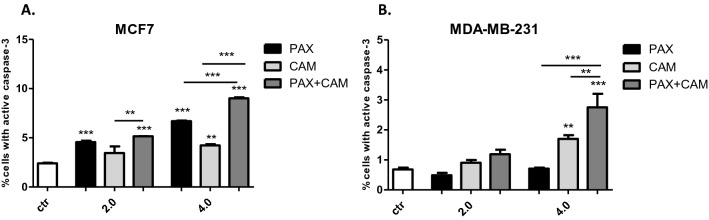
The effect of paclitaxel (PAX) and cambinol (CAM) on the caspase-3 activation in (**A**) MCF7 and (**B**) MDA-MB-231 breast cancer (BC) cells. The % of cells with active caspase-3 was determined after an individual or concomitant drug treatment for 48 h using selected ratios of the IC_50_ determined in the (4,5-dimethylthiazol-2-yl)-2,5-diphenyltetrazolium bromide (MTT) assay (2.0 = IC_50_ + IC_50_, 4.0 = 2IC_50_ + 2IC_50_). Data are presented as mean ± standard deviation (± SD) of the mean, *n* = 3 per concentration from three independent experiments. One-way ANOVA, Tukey’s post hoc testing. p* < 0.05, **p < 0.01, ***p < 0.001

**Fig. 7:**
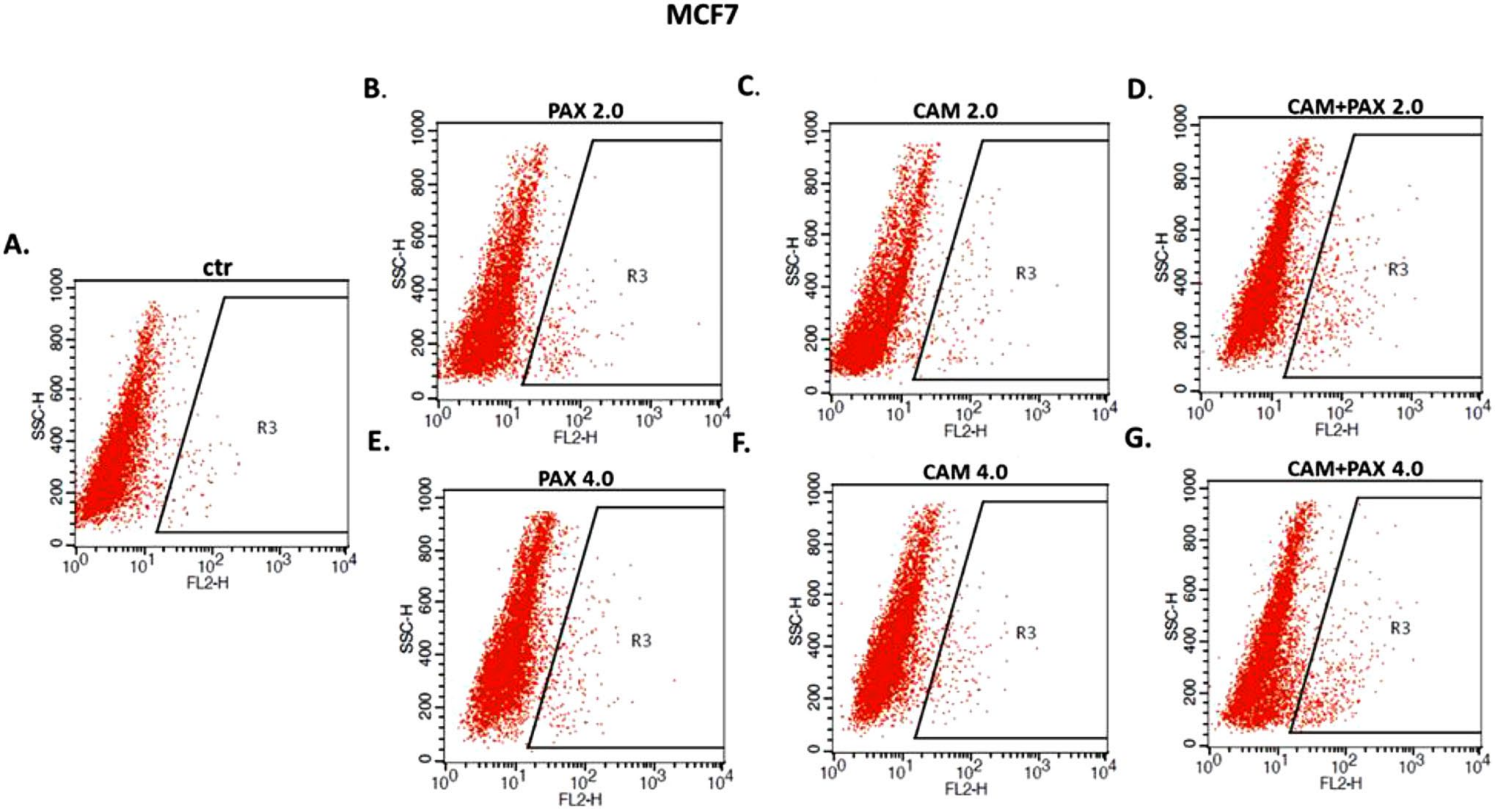
The effect of paclitaxel (PAX) and cambinol (CAM) on the induction of apoptosis in MCF7 luminal breast cancer (BC) cells. Representative dot plots from the fluorescence-activated cell sorting (FACS) analysis after 48 h incubation with medium (ctr) (**A**), PAX (**B**, **E**), CAM (**C**, **F**) and PAX + CAM (**D**, **G**) (2.0 = IC_50_ + IC_50_, 4.0 = 2IC_50_ + 2IC_50_). Gate R3 represents the amount of the apoptotic cells with active caspase-3

Next, we determined whether apoptosis is involved in the cytotoxic effect of CAM/PAX. Induction of apoptosis in both BC cell lines after PAX and CAM treatment applied individually or in combination was determined by FACS as a number of cells with active caspase-3 (Fig. 6). MCF7 luminal and MDA-MB-231 TNBC cells were treated with PAX and CAM for 48 h using selected ratios of the IC_50_, which were determined in the MTT test, where 2.0 = IC_50_ + IC_50_ and 4.0 = 2IC_50_ + 2IC_50_. As shown in Figs. 6 and 7, PAX used alone dose-dependently increased the number of cells with active caspase-3 in MCF7 BC cells, as follows ctr. = 2.4%, PAX_2.0_ = 4.56%, PAX_4.0_ = 6.69%. In turn, in MDA-MB-231 BC cells only CAM in 4.0 concentration statistically significantly increased in caspase-3 active cells (CAM_4.0_ = 1.7% vs. ctr. = 0.68%) (with Student’s *t* test: *t* = 6.650, *df* = 5, *p* = 0.012). PAX and CAM used in combination increased the percentage of apoptotic cells in comparison to individual treatment in MCF7 cells (both 2.0 = 5.15% and 4.0 = 9.02%) and MDA-MB-231 BC cells (only 4.0 = 2.75%), suggesting that CAM strengthens the effect of PAX in these combinations (Fig. 6).

### Cell cycle arrest in MCF7 and MDA-MB-231 BC cells after CAM and PAX treatment administered singly and in combination

**Fig. 8:**
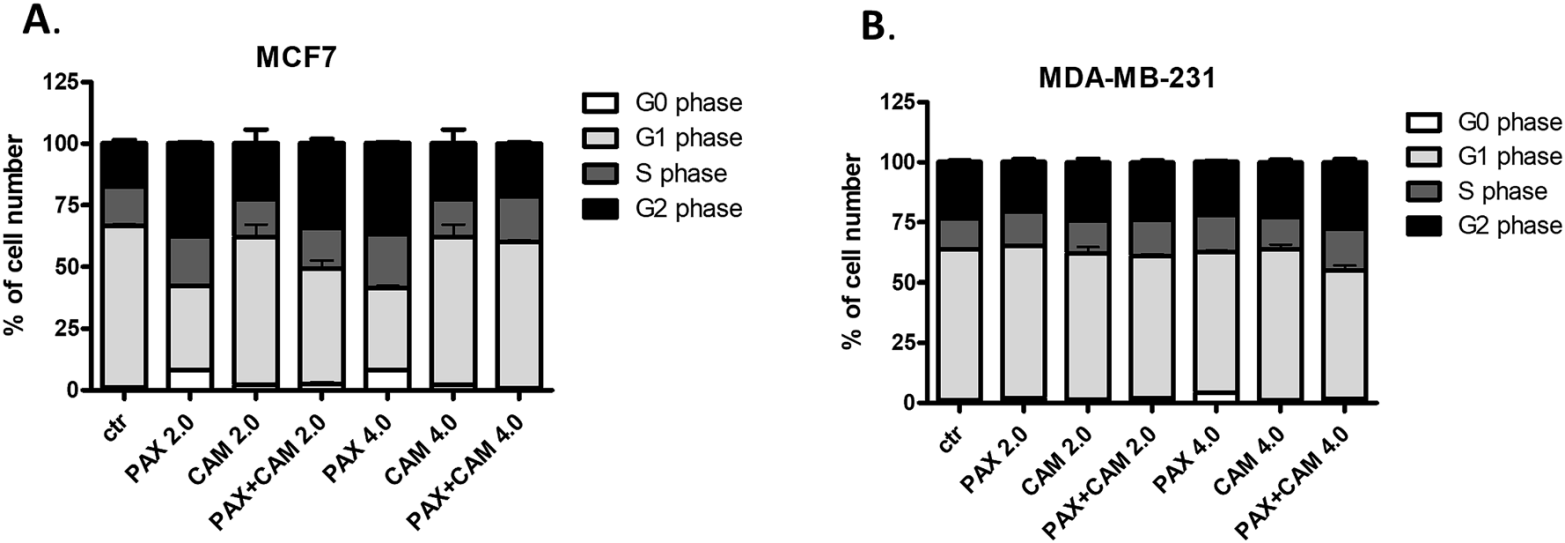
The effect of paclitaxel (PAX) and cambinol (CAM) on the cell cycle progression in (**A**) MCF7 luminal and (**B**) MDA-MB-231 triple-negative breast cancer (TNBC) cells. BC cells were exposed to the individual or concomitant drug treatments for 48 h using selected ratios of the IC_50_ determined in the (4,5-dimethylthiazol-2-yl)-2,5-diphenyltetrazolium bromide (MTT assay) (2.0 = IC_50_ + IC_50_, 4.0 = 2IC_50_ + 2IC_50_), stained with propidium iodide (PI) and analyzed by fluorescence-activated cell sorting (FACS). Data are presented as mean ± standard deviation (± SD) of the mean, *n* = 4 per concentration from three independent experiments

**Fig. 9:**
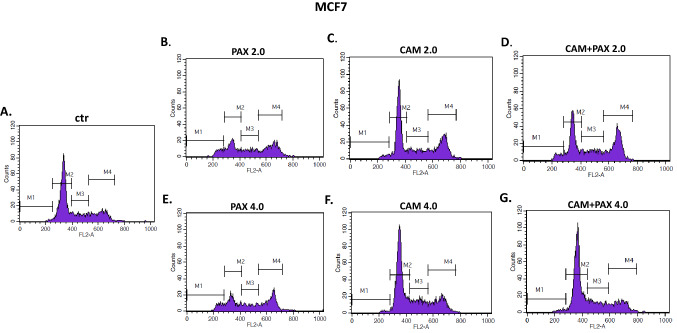
The effect of paclitaxel (PAX) and cambinol (CAM) on the cell cycle progression in MCF7 luminal breast cancer (BC) cells after 48-h incubation with medium (ctr) (**A**), PAX (**B**, **E**), CAM (**C**, **F**) and PAX + CAM (**D**, **G**) (2.0 = IC_50_ + IC_50_, 4.0 = 2IC_50_ + 2IC_50_). Gates on the representative histograms from the fluorescence-activated cell sorting (FACS) analysis: *M1* pre-G1 phase, *M2* G1 phase, *M3* S phase; *M4* G2 phase

**Fig. 10:**
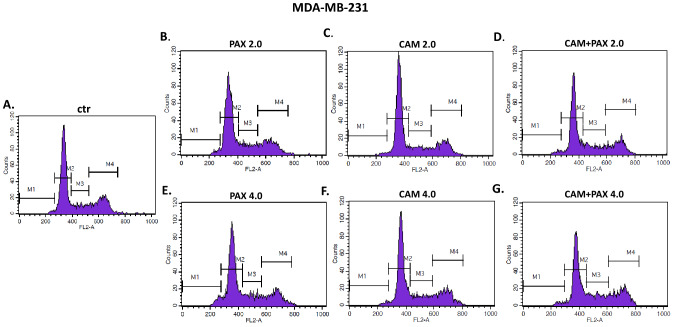
The effect of paclitaxel (PAX) and cambinol (CAM) on the cell cycle progression in MDA-MB-231 triple-negative breast cancer (TNBC) cells after 48-h incubation with medium (ctr) (**A**), PAX (**B**, **E**), CAM (**C**, **F**) and PAX + CAM (**D**, **G**) (2.0 = IC_50_ + IC_50_, 4.0 = 2IC_50_ + 2IC_50_). Gates on the representative histograms from the fluorescence-activated cell sorting (FACS) analysis: *M1* pre-G1 phase, *M2* G1 phase, *M3* S phase, *M4* G2 phase

To further analyze the mechanism by which CAM and PAX inhibited the proliferation of BC cells, we performed cell cycle analysis by means of FACS. The influence of PAX and CAM used individually or in combination on the cell cycle progression was determined using PI-staining. FACS analysis revealed that treatment of MCF7 BC cells with PAX separately for 48 h leads to the accumulation of BC cells in the pre-G1 (ctr. = 1.05%, 2.0 = 8.22%, 4.0 = 8.23%) and G2 phases (ctr. = 17.73%, 2.0 = 37.98%, 4.0 = 37.16%). However, this effect was only evident in luminal subtype of BC. Incubation of BC cells with CAM caused cell cycle arrest in the G2 phase in MCF7 cell line (ctr. = 17.72%, 2.0 = 22.73%, 4.0 = 37.16%); similarly to PAX, no changes in the course of the cell cycle were observed in the MDA-MB-231 TNBC cells after CAM treatment. Concomitant treatment with PAX and CAM tended to be intermediate between both drugs (Figs. 8, 9, 10) in MCF7 BC cells. In MDA-MB-231 TNBC cells (Figs. 8, 10), the changes in the cell cycle progression were not observed.

## Discussion

Despite significant advances in the understanding of the BC biology and implementation of the new therapeutic options, resulting in a substantial decrease in both disability and death of BC patients [19, 20], chemotherapy for this very heterogeneous disease still does not bring the expected results [21]. Thus, combinations of established anti-cancer chemotherapeutics and novel targeted active agents are being tested to improve the clinical outcomes of oncological patients [22–24].

Chemotherapy with PAX is used primarily in TNBC patients [25]. However, a phenomenon of the drug resistance to standard medicaments used in the other subtypes of BC, e.g., luminal, has extended the use of PAX also in non-TNBC subtypes of BC. Moreover, it is known that therapy with PAX is limited by the many adverse effects, including diarrhea, hypotension, bleeding, allergic reactions, etc. [6]. Therefore, combinations of standard chemotherapeutics and new active agents with documented, remarkably lower toxicity are looked for to overcome these obstacles. In this context, both synthetic and natural compounds, including SIRTi, have become interesting classes of active agents for combined BC therapy [26, 27].

Sirtuins are a group of nicotinamide adenine dinucleotide (NAD+)-dependent protein deacetylases that regulate various biological processes ranging from gene transcription to energy metabolism. Both industry and academia have intensely studied human sirtuins as new potential therapeutic targets for a broad spectrum of diseases such as cancers, metabolic disorders or neurodegenerative diseases [28]. SIRT1 and SIRT2 have recently gathered giant attention because of their various regulatory effects in many pathological conditions. Numerous studies have found that both these sirtuins are highly expressed in a wide range of different tumors [29]. It has been revealed that SIRT1 is significantly up-regulated in invasive ductal breast carcinoma relative to normal tissue, further suggesting a role of SIRT1 in breast cancer development and progression [30]. Consequently, interest in discovering and testing sirtuin inhibitors has increased in the last decade [31]. So far, no SIRTi has been approved by the Food and Drug Administration (FDA) for cancer therapy; however, some seem to promise new anti-cancer agents. Several studies have confirmed the utility of SIRTi in combined anti-cancer therapy both in in vitro and in vivo settings [10].

In our study, we have analyzed the type of pharmacological drug–drug interactions between CAM (SIRT1 and SIRT2 inhibitor) and the PAX (standard chemotherapeutic) in luminal and TNBC cell lines differing from each other by molecular profile. Our results indicated that CAM inhibits BC cell proliferation in a dose-dependent manner in both analyzed cell lines. MCF7 luminal A cells were more resistant to CAM treatment than MDA-MB-231 TNBC cell line. As opposed to a single therapy, MCF7 cell line was more sensitive to PAX/CAM combined treatment than MDA-MB-231 TNBC cells. We have also demonstrated that both PAX and CAM administered alone reduced the proliferation of BC cells in a dose-dependent way. The more substantial anti-proliferative individual effect of both CAM and PAX treatment was observed in MCF7 luminal BC cells than in MDA-MB-231 TNBC cells. The combined PAX/CAM therapy effect was also more evident in MCF7 cells. Interestingly, combinatorial co-treatment was much more effective than individual ones. Similar to our results, CAM administered separately inhibited cell viability also in T47D luminal and MDA-MB-468 TNBC cell line in a concentration-dependent manner [10]. Additionally, results from the proliferation assay revealed cell growth inhibition in Mia-PaCa-2 cells and PANC-1 pancreatic cancer cells after CAM treatment [32]. Another study demonstrated a cytostatic effect, characterized by an altered morphology, impaired proliferation and cellular senescence as well as a decrease in the number of colonies and cellular migration in hepatocellular carcinoma (HCC) cells after CAM treatment [33, 34]. CAM at the 56 and 59 µM, respectively, acted cytotoxic against cancer cells in vitro and had a marked anti-proliferative effect in a Burkitt lymphoma in a mouse xenograft model [35].

Further, we have determined if apoptosis is involved in the cytotoxic effect of CAM and PAX. We have demonstrated that PAX and CAM used individually increased the number of apoptotic cells dose-dependently. The level of apoptosis was more evident in the MCF7 luminal than in the MDA-MB-231 TNBC cells. Interestingly, PAX and CAM used together increased the percentage of apoptotic cells in comparison to individual treatment, suggesting that CAM gently strengthens the pro-apoptotic effect of PAX. In our previous study, we have determined the effect of CAM with another chemotherapeutic–cisplatin (CDDP). Similar to our results, both agents used separately induced cell apoptosis; however, applying them in combination ameliorated anti-proliferative effect for all analyzed BC cell lines. CAM and CDDP used in combination significantly decreased the number of apoptotic cells, suggesting that CAM abolishes the effect of CDDP [10]. To further analyze the mechanism by which CAM and PAX inhibited the proliferation of BC cells, we performed a cell cycle analysis. We have noted the accumulation of BC cells in the pre-G1 and G2 phases in the MCF7 and MDA-MB-231 BC cells after PAX treatment. This effect was stronger in luminal subtype BC. Incubation of BC cells with CAM arrested the cell cycle in the G2 phase in MCF7 cell line; however, no changes were observed in the MDA-MB-231 TNBC cells. Concomitant treatment with PAX and CAM tended to be intermediate between both drugs. Like a single therapy, the effect was much more apparent in MCF7 luminal than TNBC MDA-MB-231 cells, where the changes in the cell cycle progression were not significant.

Using the advanced isobolography method, we have determined additive pharmacological interaction between CAM and PAX in both MCF7 luminal and MDA-MB-231 TNBC cells, proving that these compounds can be used in BC polytherapy in the future. CAM has been extensively studied in combination with other active agents in numerous types of cancer, showing their beneficial effects. Combining SIRTi and HDIs resulted in a synergistic antileukemic effect. Interestingly, CAM and FK866 enhanced HDIs activity in leukemia cells, but not in healthy leukocytes or hematopoietic progenitors [36]. In turn, the combination of CAM with gefitinib led to a synergistic inhibitory effect on cell growth for Mia-PaCa-2 cells and PANC-1 pancreatic cancer cells. Moreover, the combinatory regimen of CAM and gefitinib caused cell cycle arrest but no induction of apoptosis in Mia-Paca-2 cells [32]. In another study, CAM enhanced the inhibitory effect of sorafenib in HCC cell lines. Cell cultures treated with sorafenib in combination with CAM showed a more remarkable fall in cellular viability, proliferation, migration, invasion as well as induction of apoptosis and cell cycle arrest when compared with cells treated only with sorafenib. It has been confirmed that induction of caspase-3/7, cyclin D1 and proliferation index-67 (Ki-67) protein expression by CAM are probably involved in enhancing the sensitivity of HCC cells to sorafenib [37]. In turn, silybin, a natural flavonolignan, treatment resulted in the decrease of the A549 lung adenocarcinoma cells’ viability, adhesion and migratory capability as well as an increase in apoptosis and reactive oxygen species level. CAM enhanced the anti-cancer activity of silybin. Due to silybin being a potent inhibitor of lung adenocarcinoma cell growth that interferes with SIRT1 signaling, combinatorial treatment with CAM/silybin may be used for therapeutic intervention in lung adenocarcinoma therapy in the future [38]. Interestingly, CAM can also sensitize neuroblastoma cells to doxorubicin (DOX). DOX applied alone had no efficacy in the DOX resistance tumors, but co-treatment with CAM suppressed tumor growth in 70% of volume [39].

These results are very promising and may create a positive attitude to continue our research in other pre-clinical models. Good anti-cancer properties of CAM individually or in combined therapy make this drug a subject of clinical interest as a new approach in the BC regimens. However, further studies are needed to elucidate the targets and molecular mechanisms of CAM/PAX action in other pre-clinical models.

## Supplementary Information

Below is the link to the electronic supplementary material.

Supplementary file1 (PZF 588 kb)

Supplementary file2 (PZF 426 kb)

Supplementary file3 (PDF 1191 kb)

Supplementary file4 (PDF 1097 kb)

Supplementary file5 (PZF 421 kb)

Supplementary file6 (PZF 422 kb)

Supplementary file7 (PZF 367 kb)

Supplementary file8 (PZF 365 kb)

Supplementary file9 (PZF 433 kb)

Supplementary file10 (PZF 362 kb)

Supplementary file11 (PZF 252 kb)

Supplementary file12 (PZF 251 kb)

Supplementary file13 (PDF 337 kb)

Supplementary file14 (PDF 335 kb)

Supplementary file15 (PDF 318 kb)

## Data Availability

All data generated or analysed during this study are included in this published article and its supplementary information files.

## Abbreviations

ANOVA: Analysis of variance
ATTC: American Type Culture Collection
BC: Breast cancer
BrdU: 5-Bromo-2'-deoxyuridine
CAM: Cambinol
CDDP: Cisplatin
CRR: Concentration–response relationship
DMEM: Dulbecco's modified Eagle's medium
DMSO: Dimethyl sulfoxide
DOX: Doxorubicin
ER+: Estrogen receptor positive
F ratio: Factor for slope function ratio
FACS: Fluorescence-activated cell sorting
FBS: Fetal bovine serum
FDA: Food and Drug Administration
HATs: Histone acetyltransferases
HCC: Hepatocellular carcinoma
HCl: Hydrochloric acid
HDACs: Histone deacetylases
HDIs: Histone deacetylase inhibitors
IC_50 add_: IC_50_ theoretically calculated as an additive
IC_50 mix_: IC_50_ for the mixture
Ki-67: Proliferation index-67
MTT: 3-(4,5-Dimethylthiazol-2-yl)-2,5-diphenyltetrazolium bromide
*n*: Number of items
*n*_add_: Number of items calculated for the additive mixture
NC: Not collateral
*n*_mix_: Number of items for the experimental mixture
NAD: Nicotinamide adenine dinucleotide
PAX: Paclitaxel
PBS: Phosphate-buffered saline
PI: Propidium iodide
RT: Room temperature
S: Slope function ratio
SD: Standard deviation
SDS: Sodium dodecyl sulfate
SEM: Standard error of the mean
SIRTi: Sirtuin inhibitor
TMB: Tetramethylobenzidine
TNBC: Triple-negative breast cancer
