# Supplementary material for: Additive pharmacological interaction between sirtuin inhibitor cambinol and paclitaxel in MCF7 luminal and MDA-MB-231 triple-negative breast cancer cells
Source: Pharmacol Rep. 2022 Jul 28;74(5):1011–24. doi: 10.1007/s43440-022-00393-w (PMC9585000; doi:10.1007/s43440-022-00393-w)
Supplement: Supplementary file 3 — Supplementary file3 (PDF 1191 kb) [file 43440_2022_393_MOESM3_ESM.pdf]

CAM

log(dose)	DOSE	EFFECT	TOTAL	%	PROBITS	Log(dose)*PROBIT	Log(dose)^2
1.69897	**50**	**30.47**	**100**	30.5%	4.48905	7.626761298	2.886499076
1.77815	**60**	**62.46**	**100**	62.5%	5.31755	9.455408181	3.161821869
1.84510	**70**	**69.3**	**100**	69.3%	5.50435	10.1560654	3.404386777
1.90309	**80**	**74.2**	**100**	74.2%	5.64950	10.75150688	3.621751499
1.95424	**90**	**80.39**	**100**	80.4%	5.85560	11.44326244	3.819063786
9.179552	350	316.82	500	3.1682	26.81605	49.4330042	16.89352301

N **120**

n' 5

a	**4.9433822**		
b	**-3.7123965**		
ED_16	**36.31998983**	1.560146	
ED_50	**57.86771987**	1.762436	
ED_84	**92.19917238**	1.964727	
ED_97	**138.9647432**	2.142905	
S	**1.593274672**		
f_ED_50	**1.124999361**		
N	**120**	10.95445	0.252865
CL lower	**51.43800243**		
CL upper	**65.10114789**		
ED_50	**57.86771987**		
SE(ED_50)	**3.475876968**		
ED_16	**36.31998983**		
SE(ED_16)	**2.181593061**		
ED_84	**92.19917238**		
SE(ED_84)	**5.538026735**		

ANOVA	
r^2	0.89532
Mean	5.3632
SSt	1.1106
SSreg	0.9943
SSres	0.1163
SSt	1.1106
MSreg	0.9943
MSres	0.0388
**F-test**	**25.658**

Expected

x	y=ax+b	%
1.69897	4.686261	37.7%
1.778151	5.077685	53.1%
1.845098	5.408628	65.9%
1.90309	5.695305	75.7%
1.954243	5.948171	82.8%

		(E-O)^2/E*(100-E)	(E-O)^2/E
f ratio $S_1 = A^{2.77/sqrtN'}$	1.108126		
$A = 10^{\gamma}$	1.500845	0.0222	1.38154
$\gamma = 1.1\ (\log S)^2 / \log R$	0.176336	0.0352	1.65194
R = (largest / smallest) dose	1.8	0.0053	0.17986
log R	0.255273	0.0012	0.02804
log S	0.202291	0.0042	0.07289
$(\log S)^2$	0.040922		
largest dose	90	χ_{LW} (Chi) ^{2}	χ (Chi) ^{2}
smallest dose	50	**6.805844**	3.31427

PAX

log(dose)	DOSE	EFFECT	TOTAL	%	PROBITS	Log(dose)*PROBIT	Log(dose)^2
-2.30103	**0.005**	**33.05**	**100**	33.1%	4.56145	-10.49603327	5.294739041
-2.00000	**0.01**	**41.52**	**100**	41.5%	4.78580	-9.5716	4
-1.30103	**0.05**	**68.39**	**100**	68.4%	5.47860	-7.127822934	1.69267905
-5.60206	0.065	142.96	300	1.4296	14.82585	-27.19545621	10.98741809

N **72**

n' 3

a	**0.9301880**		
b	**6.6789396**		
ED_16	**0.001318262**	-2.879998	
ED_50	**0.015669438**	-1.804947	
ED_84	**0.186253712**	-0.729895	
ED_97	**1.648201565**	0.21701	
S	**11.88643245**		
f_ED_50	**2.243613393**		
N	**72**	8.485281	0.326448
CL lower	**0.006984019**		
CL upper	**0.03515616**		
ED_50	**0.015669438**		
SE(ED_50)	**0.006457424**		
ED_16	**0.001318262**		
SE(ED_16)	**0.00054326**		
ED_84	**0.186253712**		
SE(ED_84)	**0.076755729**		

ANOVA	
r^2	0.99629
Mean	4.9420
SSt	0.4572
SSreg	0.4555
SSres	0.0017
SSt	0.4572
MSreg	0.4555
MSres	0.0017
**F-test**	**268.680**

Expected

x	y=ax+b	%
-2.30103	4.538549	32.2%
-2	4.818564	42.8%
-1.30103	5.468737	68.0%

		(E-O)^2/E*(100-E)	(E-O)^2/E
f ratio $S_2 = A^{2.77/sqrtN'}$	2.600255		
$A = 10^{\gamma}$	18.6771	0.0003	0.02128
$\gamma = 1.1\ (\log S)^2 / \log R$	1.271309	0.0007	0.03832
R = (largest / smallest) dose	10	0.0001	0.00184
log R	1		
log S	1.075052		
$(\log S)^2$	1.155736		
largest dose	0.05	χ_{LW} (Chi) ^{2}	χ (Chi) ^{2}
smallest dose	0.005	**0.104160**	0.06144

Elaborated by J.J. Luszczki

log (f_S_1)^2	0.001988194
log (f_S_2)^2	0.172238222
sqrt (N31+N32)	0.41740438
Slope Ratio (S.R.)	7.460378719
f ratio (S.R.)	2.614594719
**Test for Parallelism**	**0.350464074**
**NOT Parallel**	

log (f_ED_50_1)	0.002616555	0.354656271
log (f_ED_50_2)	0.123164515	0.464599715
sqrt (Q42+Q43)	0.354656271	0.595822536
		2.26285263
Potency Ratio (P.R.)	3693.031056	2.91473929
f ratio (P.R.)	2.26285263	3.942961491

**Test of Significance**	**1632.024554**	1632.024554 at (p<0.05)
**SIGNIFICANT at**	*****	1267.019341 at (p<0.01)
		936.6135236 at (p<0.001)

1:1

log(dose)	DOSE	EFFECT	TOTAL	CAM	PAX	%	PROBITS	Log(dose)*PROBIT	Log(dose)^2
				DOSE					
1.06358	**11.577**	**30.98**	**100**	**11.57357**	**0.003133894**	31.0%	4.50355	4.789907232	1.131212582
1.36461	**23.153**	**37.36**	**100**	**23.14713**	**0.006267787**	37.4%	4.67765	6.383190301	1.862173483
1.54070	**34.73**	**52.61**	**100**	**34.7206**	**0.009401653**	52.6%	5.06545	7.804363045	2.373771229
1.66564	**46.307**	**60.38**	**100**	**46.29416**	**0.012535547**	60.4%	5.26315	8.766533337	2.774369377
1.81218	**64.89**	**64.89**	**100**	**64.87243**	**0.017566176**	64.9%	5.38235	9.753775043	3.283988285
7.446726	180.6568	246.22	500			2.4622	24.89215	37.49776896	11.42551496
N	**120**								
n'	5								

a	**1.2688286**		
b	**3.0887062**		
ED_16	**5.226568016**	0.718217	
ED_50	**32.08818263**	1.506345	
ED_84	**197.0033608**	2.294474	
ED_97	**974.2204253**	2.988657	
S	**6.139436536**		
f_ED_50	**1.582305406**		
N	**120**	10.95445	0.252865
CL lower	**20.27938634**		
CL upper	**50.77330484**		
ED_50	**32.08818263**		
SE(ED_50)	**7.509212115**		
ED_16	**5.226568016**		
SE(ED_16)	**1.22311096**		
ED_84	**197.0033608**		
SE(ED_84)	**46.10233122**		

ANOVA	
r^2	0.94925
Mean	4.9784
SSt	0.5678
SSreg	0.5390
SSres	0.0288
SSt	0.5678
MSreg	0.5390
MSres	0.0096
**F-test**	**56.112**

Expected

x	y=ax+b	%
1.063585	4.438213	28.7%
1.364615	4.820168	42.9%
1.540705	5.043597	51.7%
1.665644	5.202123	58.0%
1.812178	5.388049	65.1%

		(E-O)^2/E*(100-E)	(E-O)^2/E
f ratio $S_2 = A^{2.77/sqrtN'}$	1.701363		
$A = 10^{\gamma}$	8.179509	0.0025	0.17905
$\gamma = 1.1\ (\log S)^2 / \log R$	0.912727	0.0124	0.70663
R = (largest / smallest) dose	5.605224	0.0003	0.01474
log R	0.748593	0.0023	0.09699
log S	0.788129	0.0000	0.00067
$(\log S)^2$	0.621147		
largest dose	64.89	χ_{LW} (Chi) ^{2}	χ (Chi) ^{2}
smallest dose	11.5767	**1.751336**	0.99808

MCF7 IC50

CAM	**57.868**	µg/ml
PAX	**0.0157**	µg/ml

NON_parallel DRRCs

ISOBOLOGRAPHY	0.025	0.05	0.1	0.20	0.40	0.60	0.80	1.00	1.20	1.40	1.60	1.80	2.00	2.20
CAM	0.72	1.45	2.89	5.79	11.57	17.36	23.15	28.93	34.72	40.51	46.29	52.08	57.87	63.65
PAX	0.00	0.00	0.00	0.00	0.00	0.00	0.01	0.01	0.01	0.01	0.01	0.01	0.02	0.02
Mixture (1:1)	**0.724**	**1.447**	**2.894**	**5.788**	**11.577**	**17.365**	**23.1534**	**28.9417**	**34.7300**	**40.5184**	**46.3067**	**52.0951**	**57.8834**	**63.6717**

ISOBOLOGRAPHY	2.40	2.60	2.80	3.00	3.20	3.40	3.60	3.80	4.00	4.20	4.40	4.60	4.80	5.00
CAM	69.44	75.23	81.01	86.80	92.59	98.38	104.16	109.95	115.74	121.52	127.31	133.10	138.88	144.67
PAX	0.02	0.02	0.02	0.02	0.03	0.03	0.03	0.03	0.03	0.03	0.03	0.04	0.04	0.04
Mixture (1:1)	**69.4601**	**75.248**	**81.037**	**86.825**	**92.613**	**98.402**	**104.190**	**109.978**	**115.767**	**121.555**	**127.343**	**133.132**	**138.920**	**144.708**

PAX

[uM]	ctr	0.001	0.005	0.01	0.05	0.1	0.5	1
		**4.66**	**33.05**	**41.52**	**68.39**	**66.55**	**73.71**	**77.08**
**Mean**	100	95.34	66.95	58.48	31.61	33.45	26.29	22.92
**SE**	4.655	4.492	6.445	6.613	1.84	1.976	1.79	1.864

CAM

[mM]	ctr	0.01	0.02	0.03	0.04	0.05	0.06	0.07	0.08	0.09	0.1
		**-7.3**	**-8.3**	**-2.9**	**2.7**	**30.47**	**62.46**	**69.3**	**74.2**	**80.39**	**89.18**
**Mean**	100	107.3	108.3	102.9	97.3	69.53	37.54	30.7	25.8	19.61	10.82
**SE**	2.755	1.424	2.555	2.758	2.411	2.207	1.478	1.756	3.479	1.291	1.054

MIX

		11.5767	17.3650	23.1534	28.9417	34.7300	40.5184	46.3067	52.0951	57.8834	63.6717
	**ctr**	**0.4**	**0.6**	**0.8**	**1**	**1.2**	**1.4**	**1.6**	**1.8**	**2**	**2.2**
		**30.98**	**35.54**	**37.36**	**46.25**	**52.61**	**54.95**	**60.38**	**65**	**64.89**	**67.18**
**Mean**	100	69.02	64.46	62.64	53.75	47.39	45.05	39.62	35	35.11	32.82
**SE**	2.266	3.972	4.952	4.695	3.458	3.281	3.298	2.425	1.736	1.497	1.526

CAM				
a	4.943382155			
b	-3.7123965			
			SEM	
4 probit	1.560145718	36.32		
5 probit	1.76243637	57.868	3.48	
6 probit	1.964727023	92.20		
p	6.108			

PAX				
a	0.930187972			
b	6.678939608			
			SEM	
4 probit	-2.879998116	0.00		
5 probit	-1.804946589	0.016	0.0065	
6 probit	-0.729895063	0.19		
q	0.683			

0	54.39		0.00	0.02
0	57.87		28.93386	0.007835
0	61.34	01:01	28.93386	0.007835
			28.93386	0.007835
0.01	0		57.87	0
0.02	0			
0.02	0			

		a	b	sum (a+b)
		28.93385994	0.007835	28.94169
01:01	proportions	0.999729293	0.000271	

0	0
69.44126	0.01880
1.2	1.2

01:01	ED50 mix	a	b
SEM	24.579	24.57231682	0.006653699
7.509	32.088	32.07949614	0.008686495
	39.597	39.58667546	0.010719291

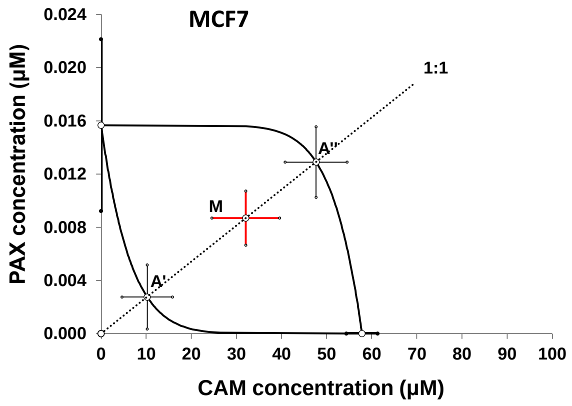

Lower

		ED50 add		SEM (a)	SEM total
X	47.6329	0.010248061	47.6780106		6.8637
Y	0.0129	0.013	47.6780106	0.00265	
		0.02	47.6780106	0.006456	
		ED50 add		SEM (b)	SUM
		0.01	40.8170106		47.69091
		0.013	47.6780106	6.861	
		0.01	54.5390106	73327.83	

Upper

		ED50 add		SEM (a)	SEM total
X	10.1657	0.000342665	10.226		5.5934
Y	0.003	0.003	10.226	0.00241	
		0.005162665	10.226	0.006457	
		ED50 add		SEM (b)	SUM
		0.00	4.635		10.22856
		0.003	10.226	5.591	
		0.003	15.817	15726.7	

ED50 mix		SEM (a)	SEM total
24.572	0.009		7.50921
32.079	0.009	7.507179	
39.587	0.009		
ED50 mix		SEM (b)	
32.079	0.007		
32.079	0.009	0.002033	
32.079	0.011		

	(a)	(b)	sum (a+b)
	28.93385994	0.007835	28.94169
proportions	0.999729293	0.000271	

109
0.00009

lower additivity	b	a	expression 1	q/p	expression 2
	0.0000	57.868			
0.000248	0.0002	57.76457619	0.984170482	0.1118062	0.998217595
0.0002	0.0005	57.65994834	0.968340964	0.1118062	0.996409543
0.0002	0.0007	57.55379018	0.952511446	0.1118062	0.994575047
0.0002	0.0010	57.44605334	0.936681928	0.1118062	0.992713269
0.0002	0.0012	57.33668704	0.920852411	0.1118062	0.990823332
0.0002	0.0015	57.22564	0.905022893	0.1118062	0.988904317
0.0002	0.0017	57.11285018	0.889193375	0.1118062	0.986955254
0.0002	0.0020	56.99826469	0.873363857	0.1118062	0.984975126
0.0002	0.0022	56.88181949	0.857534339	0.1118062	0.982962861
0.0002	0.0025	56.76344922	0.841704821	0.1118062	0.980917329
0.0002	0.0027	56.64308493	0.825875	0.1118062	0.978837339
0.0002	0.0030	56.52065385	0.810046	0.1118062	0.976721633
0.0002	0.0032	56.39607902	0.794216267	0.1118062	0.974568881
0.0002	0.0035	56.26927904	0.778386749	0.1118062	0.972377677
0.0002	0.0037	56.14016769	0.762557232	0.1118062	0.970146531
0.0002	0.0040	56.00865351	0.746727714	0.1118062	0.967873862
0.0002	0.0042	55.8746394	0.730898196	0.1118062	0.965557992
0.0002	0.0045	55.73802211	0.715068678	0.1118062	0.963197137
0.0002	0.0047	55.59869176	0.699239	0.1118062	0.960789398
0.0002	0.0050	55.45653116	0.683409642	0.1118062	0.958332751
0.0002	0.0052	55.31141525	0.667580124	0.1118062	0.955825033
0.0002	0.0055	55.16321026	0.651750606	0.1118062	0.953263933
0.0002	0.0057	55.01177296	0.635921088	0.1118062	0.950646977
0.0002	0.0060	54.85694972	0.62009157	0.1118062	0.947971509
0.0002	0.0062	54.69857543	0.604262053	0.1118062	0.945234676
0.0002	0.0064	54.53647233	0.588432535	0.1118062	0.942433406
0.0002	0.0067	54.37044869	0.572603017	0.1118062	0.939564386
0.0002	0.0069	54.20029728	0.556773499	0.1118062	0.936624035
0.0002	0.0072	54.02579364	0.540943981	0.1118062	0.933608474
0.0002	0.0074	53.84669405	0.525114463	0.1118062	0.930513491
0.0002	0.0077	53.66273331	0.509284945	0.1118062	0.927334504
0.0002	0.0079	53.47362205	0.493455427	0.1118062	0.924066512
0.0002	0.0082	53.27904371	0.477625909	0.1118062	0.920704044
0.0002	0.0084	53.07865095	0.461796392	0.1118062	0.917241099
0.0002	0.0087	52.8720615	0.445966874	0.1118062	0.913671069
0.0002	0.0089	52.65885326	0.430137356	0.1118062	0.909986662
0.0002	0.0092	52.43855849	0.414307838	0.1118062	0.906179794
0.0002	0.0094	52.21065693	0.39847832	0.1118062	0.902241475
0.0002	0.0097	51.97456752	0.382648802	0.1118062	0.898161663
0.0002	0.0099	51.72963841	0.366819284	0.1118062	0.893929094
0.0002	0.0102	51.4751349	0.350989766	0.1118062	0.889531072
0.0002	0.0104	51.21022	0.335160248	0.1118062	0.884953211
0.0002	0.0107	50.93395882	0.31933073	0.1118062	0.880179121
0.0002	0.0109	50.64525023	0.303501213	0.1118062	0.875190008
0.0002	0.0112	50.34284342	0.287671695	0.1118062	0.869964179
0.0002	0.0114	50.02527831	0.271842177	0.1118062	0.864476403
0.0002	0.0117	49.69084243	0.256012659	0.1118062	0.858697086
0.0002	0.0119	49.33750845	0.240183141	0.1118062	0.852591195
0.0002	0.0122	48.96285091	0.224353623	0.1118062	0.846116816
0.0002	0.0124	48.5639332	0.208524105	0.1118062	0.839223203
0.0002	0.0127	48.13715105	0.192694587	0.1118062	0.831848069
0.0002480	0.0129	47.6780106	0.176865069	0.1118062	0.823913759
0.0000480	0.0129	47.58488798	0.173799251	0.1118062	0.822304526
0.0000	0.0130	47.49029473	0.170733433	0.1118062	0.82066988
0.0000	0.0130	47.3941806	0.167667615	0.1118062	0.819008952
0.0000	0.0131	47.29649263	0.164601797	0.1118062	0.817320826
0.0000	0.0131	47.19717501	0.16153598	0.1118062	0.815604539
0.0000	0.0132	47.09616884	0.158470162	0.1118062	0.813859073
0.0000	0.0132	46.99341189	0.155404344	0.1118062	0.812083351
0.0000	0.0133	46.88883837	0.152338526	0.1118062	0.810276238
0.0000	0.0133	46.78237861	0.149272708	0.1118062	0.808436529
0.0000	0.0134	46.67395878	0.14620689	0.1118062	0.806562949
0.0000	0.0134	46.56350054	0.143141072	0.1118062	0.804654143
0.0000	0.0135	46.45092065	0.140075254	0.1118062	0.802708673
0.0000	0.0135	46.33613056	0.137009436	0.1118062	0.80072501
0.0000	0.0136	46.21903592	0.133943618	0.1118062	0.798701522
0.0000	0.0136	46.09953612	0.1308778	0.1118062	0.796636471
0.0000	0.0137	45.97752362	0.127811982	0.1118062	0.794527998
0.0000	0.0137	45.85288339	0.124746164	0.1118062	0.792374116
0.0000	0.0138	45.72549211	0.121680346	0.1118062	0.790172694
0.0000	0.0138	45.5952174	0.118614528	0.1118062	0.787921444
0.0000	0.0139	45.46191688	0.11554871	0.1118062	0.785617905
0.0000	0.0139	45.32543713	0.112482892	0.1118062	0.783259427
0.0000	0.0140	45.18561248	0.109417074	0.1118062	0.780843147
0.0000	0.0140	45.04226372	0.106351256	0.1118062	0.778365967
0.0000	0.0141	44.89519647	0.103285438	0.1118062	0.775824528
0.0000	0.0141	44.7441995	0.10021962	0.1118062	0.773215181
0.0000	0.0141	44.58904264	0.097153802	0.1118062	0.770533948
0.0000	0.0142	44.42947443	0.094087984	0.1118062	0.767776483
0.0000	0.0142	44.2652194	0.091022166	0.1118062	0.764938026
0.0000	0.0143	44.09597484	0.087956348	0.1118062	0.762013346
0.0000	0.0143	43.92140712	0.08489053	0.1118062	0.758996678
0.0000	0.0144	43.74114718	0.081824712	0.1118062	0.755881643
0.0000	0.0144	43.55478534	0.078758894	0.1118062	0.752661163
0.0000	0.0145	43.36186504	0.075693076	0.1118062	0.749327348
0.0000	0.0145	43.16187529	0.072627258	0.1118062	0.745871366
0.0000	0.0146	42.95424156	0.06956144	0.1118062	0.742283291
0.0000	0.0146	42.7383147	0.066495622	0.1118062	0.738551904
0.0000	0.0147	42.51335722	0.063429804	0.1118062	0.734664461
0.0000	0.0147	42.27852635	0.060363986	0.1118062	0.730606397
0.0000	0.0148	42.03285271	0.057298168	0.1118062	0.726360963
0.0000	0.0148	41.77521335	0.05423235	0.1118062	0.721908751
0.0000	0.0149	41.504297	0.051166532	0.1118062	0.717227102
0.0000	0.0149	41.21855898	0.048100714	0.1118062	0.712289322
0.0000	0.0150	40.91616158	0.045034896	0.1118062	0.707063656
0.0000	0.0150	40.59489386	0.041969078	0.1118062	0.701511896
0.0000	0.0151	40.25206189	0.03890326	0.1118062	0.695587488
0.0000	0.0151	39.88433498	0.035837442	0.1118062	0.689232876
0.0000	0.0152	39.4875248	0.032771624	0.1118062	0.682375682
0.0000	0.0152	39.05625872	0.029705806	0.1118062	0.674923063
0.0000	0.0153	38.58347949	0.026639988	0.1118062	0.666753063
0.0000	0.0153	38.05964652	0.02357417	0.1118062	0.657700815
0.0000	0.0153	37.47139374	0.020508352	0.1118062	0.647535341
0.0000	0.0154	36.79912617	0.017442534	0.1118062	0.635918026
0.0000	0.0154	36.01234424	0.014376716	0.1118062	0.622321811
0.0000	0.0155	35.05947214	0.011310898	0.1118062	0.605855427
0.0000	0.0155	33.84185356	0.00824508	0.1118062	0.584814014
0.0000	0.0156	32.1275393	0.005179262	0.1118062	0.555189307
	0.0157	0			

upper additivity	b	a	expression 1	q/p	expression 2	expression 3
	0.00	57.868				
0.000	0.000	26.499	239.0833511	0.1118062	1.844745444	31.36894581
0.000	0.000	23.971	119.5416756	0.1118062	1.707180499	33.89666172
0.000	0.000	22.399	79.69445037	0.1118062	1.631516068	35.4686791
0.000	0.000	21.240	59.77083778	0.1118062	1.579873942	36.62806147
0.000	0.000	20.314	47.81667022	0.1118062	1.540945577	37.55338329
0.000	0.000	19.541	39.84722518	0.1118062	1.509851901	38.32675233
0.000	0.000	18.875	34.15476444	0.1118062	1.484052576	38.99303892
0.000	0.001	18.288	29.88541889	0.1118062	1.462060792	39.57955797
0.000	0.001	17.763	26.56481679	0.1118062	1.442933327	40.10422298
0.000	0.001	17.288	23.90833511	0.1118062	1.426035363	40.5794424
0.000	0.001	16.854	21.7348501	0.1118062	1.410919834	41.0141799
0.000	0.001	16.453	19.92361259	0.1118062	1.397260381	41.41512968
0.000	0.001	16.080	18.39102701	0.1118062	1.384811711	41.78742815
0.000	0.001	15.733	17.07738222	0.1118062	1.373384944	42.13510577
0.000	0.001	15.406	15.93889007	0.1118062	1.362831645	42.46138552
0.000	0.001	15.099	14.94270944	0.1118062	1.353033114	42.76888664
0.000	0.001	14.808	14.06372654	0.1118062	1.343892989	43.05976766
0.000	0.001	14.532	13.28240839	0.1118062	1.335332007	43.33582926
0.000	0.001	14.269	12.58333427	0.1118062	1.327284211	43.59858982
0.000	0.001	14.018	11.95416756	0.1118062	1.319694146	43.84934196
0.000	0.001	13.779	11.38492148	0.1118062	1.31251476	44.08919551
0.000	0.001	13.549	10.86742505	0.1118062	1.3057058	44.31911069
0.000	0.002	13.328	10.39492831	0.1118062	1.299232567	44.53992407
0.000	0.002	13.115	9.961806296	0.1118062	1.29306495	44.75236908
0.000	0.002	12.911	9.563334044	0.1118062	1.287176655	44.95709245
0.000	0.002	12.713	9.195513504	0.1118062	1.281544592	45.15466744
0.000	0.002	12.522	8.85493893	0.1118062	1.276148379	45.34560466
0.000	0.002	12.337	8.538691111	0.1118062	1.270969933	45.53036099
0.000	0.002	12.158	8.244253486	0.1118062	1.265993147	45.70934685
0.000	0.002	11.985	7.969445037	0.1118062	1.261203607	45.88293242
0.000	0.002	11.816	7.712366165	0.1118062	1.256588369	46.05145274
0.000	0.002	11.653	7.471354722	0.1118062	1.252135765	46.21521204
0.000	0.002	11.493	7.244950033	0.1118062	1.247835243	46.3744875
0.000	0.002	11.338	7.031863268	0.1118062	1.24367723	46.52953232

0.000	0.002	11.187	6.830952889	0.1118062	1.239653016	46.68057847
0.000	0.002	11.040	6.641204197	0.1118062	1.235754651	46.82783902
0.000	0.002	10.896	6.461712192	0.1118062	1.231974865	46.97151014
0.000	0.002	10.756	6.291667134	0.1118062	1.228306989	47.11177288
0.000	0.003	10.619	6.130342336	0.1118062	1.224744891	47.24879467
0.000	0.003	10.485	5.977083778	0.1118062	1.221282924	47.38273067
0.000	0.003	10.354	5.831301246	0.1118062	1.217915873	47.51372499
0.000	**0.003**	**10.226**	**5.692460741**	**0.1118062**	**1.214638915**	**47.64191167**
0.000	0.003	9.914	5.36954716	0.1118062	1.20673392	47.95400122
0.000	0.003	9.617	5.081302471	0.1118062	1.19931248	48.25074436
0.000	0.003	9.334	4.822427897	0.1118062	1.192321329	48.53366158
0.000	0.003	9.064	4.588652134	0.1118062	1.185715421	48.80405436
0.000	0.004	8.805	4.376493818	0.1118062	1.179456332	49.06304567
0.000	0.004	8.556	4.183086982	0.1118062	1.17351103	49.3116114
0.000	0.004	8.317	4.006050832	0.1118062	1.16785092	49.55060521
0.000	0.004	8.087	3.843391248	0.1118062	1.162451089	49.78077825
0.000	0.004	7.865	3.693425345	0.1118062	1.157289703	50.0027951
0.000	0.004	7.650	3.554723033	0.1118062	1.152347524	50.21724668
0.000	0.005	7.443	3.42606127	0.1118062	1.147607518	50.42466084
0.000	0.005	7.242	3.306387896	0.1118062	1.143054532	50.62551109
0.000	0.005	7.047	3.194792802	0.1118062	1.138675028	50.82022389
0.000	0.005	6.859	3.090484733	0.1118062	1.134456867	51.0091847
0.000	0.005	6.675	2.992772505	0.1118062	1.13038912	51.19274316
0.000	0.005	6.497	2.901049669	0.1118062	1.126461914	51.3712174
0.000	0.006	6.323	2.814781909	0.1118062	1.122666304	51.54489778
0.000	0.006	6.154	2.73349663	0.1118062	1.118994159	51.71405
0.000	0.006	5.989	2.656774296	0.1118062	1.115438066	51.87891792
0.000	0.006	5.828	2.584241184	0.1118062	1.111991252	52.03972584
0.000	0.006	5.671	2.515563301	0.1118062	1.108647509	52.19668057
0.000	0.006	5.518	2.450441238	0.1118062	1.105401135	52.34997327
0.000	0.007	5.368	2.388605809	0.1118062	1.102246883	52.49978092
0.000	0.007	5.221	2.329814328	0.1118062	1.09917991	52.64626777
0.000	0.007	5.078	2.27384743	0.1118062	1.09619574	52.78958651
0.000	0.007	4.938	2.220506333	0.1118062	1.093290228	52.92987934
0.000	0.007	4.800	2.16961048	0.1118062	1.090459527	53.06727894
0.000	0.007	4.666	2.120995499	0.1118062	1.08770006	53.20190926
0.000	0.008	4.534	2.074511425	0.1118062	1.085008497	53.33388635
0.000	0.008	4.404	2.030021159	0.1118062	1.082381734	53.46331895
0.000	0.008	4.277	1.987399116	0.1118062	1.07981687	53.59030914
0.000	0.008	4.153	1.946530041	0.1118062	1.077311191	53.71495287
0.000	0.008	4.030	1.907307969	0.1118062	1.074862157	53.83734042
0.000	0.008	3.910	1.869635305	0.1118062	1.072467384	53.9575569
0.000	0.009	3.792	1.833422018	0.1118062	1.070124631	54.07568257
0.000	0.009	3.676	1.798584918	0.1118062	1.067831795	54.19179325
0.000	0.009	3.562	1.76504702	0.1118062	1.065586893	54.30596065
0.000	0.009	3.449	1.732736978	0.1118062	1.063388056	54.41825262
0.000	0.009	3.339	1.701588573	0.1118062	1.061233522	54.52873348
0.000	0.009	3.230	1.671540266	0.1118062	1.059121625	54.63746421
0.000	0.010	3.123	1.642534788	0.1118062	1.05705079	54.74450272
0.000	0.010	3.018	1.614518778	0.1118062	1.055019528	54.84990404
0.000	0.010	2.914	1.587442455	0.1118062	1.053026426	54.9537205
0.000	0.010	2.812	1.56125932	0.1118062	1.051070144	55.05600192
0.000	0.010	2.711	1.535925895	0.1118062	1.049149412	55.15679577
0.000	0.010	2.612	1.511401477	0.1118062	1.047263023	55.2561473
0.000	0.011	2.514	1.487647922	0.1118062	1.045409829	55.35409968
0.000	0.011	2.417	1.464629448	0.1118062	1.043588737	55.45069416
0.000	0.011	2.322	1.442312453	0.1118062	1.041798707	55.54597014
0.000	0.011	2.228	1.420665352	0.1118062	1.040038748	55.6399653
0.000	0.011	2.135	1.399658428	0.1118062	1.038307916	55.7327157
0.000	0.011	2.043	1.379263697	0.1118062	1.036605307	55.82425587
0.000	0.012	1.953	1.359454783	0.1118062	1.03493006	55.91461889
0.000	0.012	1.864	1.340206802	0.1118062	1.033281352	56.00383649
0.000	0.012	1.776	1.32149626	0.1118062	1.031658395	56.0919391
0.000	0.012	1.689	1.303300958	0.1118062	1.030060437	56.17895593
0.000	0.012	1.603	1.285599903	0.1118062	1.028486755	56.26491504
0.000	0.012	1.518	1.268373227	0.1118062	1.026936658	56.34984339
0.000	0.013	1.434	1.251602111	0.1118062	1.025409485	56.43376691
0.000	0.013	1.351	1.235268721	0.1118062	1.0239046	56.51671054
0.000	0.013	1.269	1.21935614	0.1118062	1.022421392	56.59869829
0.000	0.013	1.188	1.203848313	0.1118062	1.020959276	56.67975328
0.000	0.013	1.108	1.188729991	0.1118062	1.01951769	56.75989778
0.000	0.013	1.029	1.173986681	0.1118062	1.018096093	56.83915326
0.000	0.014	0.950	1.159604601	0.1118062	1.016693965	56.91754043
0.000	0.014	0.873	1.145570635	0.1118062	1.015310806	56.99507927
	0.016	0.000				
