# Supplementary material for: Additive pharmacological interaction between sirtuin inhibitor cambinol and paclitaxel in MCF7 luminal and MDA-MB-231 triple-negative breast cancer cells
Source: Pharmacol Rep. 2022 Jul 28;74(5):1011–24. doi: 10.1007/s43440-022-00393-w (PMC9585000; doi:10.1007/s43440-022-00393-w)
Supplement: Supplementary file 4 — Supplementary file4 (PDF 1097 kb) [file 43440_2022_393_MOESM4_ESM.pdf]

CAM

log(dose)	DOSE	EFFECT	TOTAL	%	PROBITS	Log(dose)*PROBIT	Log(dose)^2
1.30103	**20**	**21.24**	**100**	21.2%	4.20185	5.466732887	1.69267905
1.47712	**30**	**33.33**	**100**	33.3%	4.56915	6.749188581	2.181887201
1.60206	**40**	**44.84**	**100**	44.8%	4.87025	7.802432673	2.566596216
1.69897	**50**	**58.64**	**100**	58.6%	5.21825	8.865650225	2.886499076
1.77815	**60**	**74.19**	**100**	74.2%	5.64920	10.04513204	3.161821869
7.857332	200	232.24	500	2.3224	24.5087	38.92913641	12.48948341

N **120**

n' 5

a	**2.9203202**		
b	**0.3125547**		
ED_16	**18.3098712**	1.262685	
ED_50	**40.28222907**	1.605113	
ED_84	**88.62203129**	1.947542	
ED_97	**177.4812438**	2.249152	
S	**2.200027986**		
f_ED_50	**1.220641673**		
N	**120**	10.95445	0.252865
CL lower	**33.00086336**		
CL upper	**49.17016749**		
ED_50	**40.28222907**		
SE(ED_50)	**4.095766933**		
ED_16	**18.3098712**		
SE(ED_16)	**1.86168856**		
ED_84	**88.62203129**		
SE(ED_84)	**9.010801879**		

ANOVA	
r^2	0.96052
Mean	4.9017
SSt	1.2603
SSreg	1.2106
SSres	0.0498
SSt	1.2603
MSreg	1.2106
MSres	0.0166
**F-test**	**72.996**

Expected

x	y=ax+b	%
1.30103	4.111979	18.7%
1.477121	4.626222	35.4%
1.60206	4.991083	49.6%
1.69897	5.274091	60.8%
1.778151	5.505326	69.3%

f ratio $S_1 = A^{2.77/\mathrm{sqrtN'}}$	1.170465
$A = 10^{\gamma}$	1.863526
$\gamma = 1.1 (\log S)^2 / \log R$	0.270335
R = (largest / smallest) dose	3
log R	0.477121
log S	0.342428
$(\log S)^2$	0.117257
largest dose	60
smallest dose	20

(E-O)^2/E*(100-E)	(E-O)^2/E
0.0042	0.33760
0.0019	0.12420
0.0092	0.46469
0.0020	0.07658
0.0111	0.34031
χ_{LW} (Chi)^2	χ (Chi)^2
**2.835511**	1.34337

PAX

log(dose)	DOSE	EFFECT	TOTAL	%	PROBITS	Log(dose)*PROBIT	Log(dose)^2
-3.00000	**0.001**	**12.47**	**100**	12.5%	3.84815	-11.54445	9
-2.30103	**0.005**	**33.98**	**100**	34.0%	4.58695	-10.55470954	5.294739041
-2.00000	**0.01**	**43.85**	**100**	43.9%	4.84520	-9.6904	4
-1.30103	**0.05**	**61.47**	**100**	61.5%	5.29155	-6.884465274	1.69267905
-8.60206	0.066	151.77	400	1.5177	18.57185	-38.67402481	19.98741809

N **72**

n' 4

a	**0.8498266**		
b	**6.4705274**		
ED_16	**0.001238523**	-2.9071	
ED_50	**0.018604363**	-1.73039	
ED_84	**0.279463689**	-0.55367	
ED_97	**3.039289494**	0.482772	
S	**15.02140591**		
f_ED_50	**2.421778289**		
N	**72**	8.485281	0.326448
CL lower	**0.007682108**		
CL upper	**0.045055643**		
ED_50	**0.018604363**		
SE(ED_50)	**0.008391915**		
ED_16	**0.001238523**		
SE(ED_16)	**0.000558664**		
ED_84	**0.279463689**		
SE(ED_84)	**0.126058354**		

ANOVA	
r^2	0.98050
Mean	4.6430
SSt	1.0964
SSreg	1.0750
SSres	0.0214
SSt	1.0964
MSreg	1.0750
MSres	0.0107
**F-test**	**100.543**

Expected

x	y=ax+b	%
-3	3.921048	14.0%
-2.30103	4.515051	31.4%
-2	4.770874	40.9%
-1.30103	5.364877	64.2%

f ratio $S_2 = A^{2.77/\mathrm{sqrtN'}}$	1.961813
$A = 10^{\gamma}$	7.879374
$\gamma = 1.1 (\log S)^2 / \log R$	0.896492
R = (largest / smallest) dose	50
log R	1.69897
log S	1.176711
$(\log S)^2$	1.384648
largest dose	0.05
smallest dose	0.001

(E-O)^2/E*(100-E)	(E-O)^2/E
0.0020	0.17333
0.0031	0.21446
0.0035	0.20719
0.0033	0.11934
χ_{LW} (Chi)^2	χ (Chi)^2
**1.198696**	0.71432

Elaborated by J.J. Luszczki

log (f_S_1)^2	0.004672876
log (f_S_2)^2	0.085648473
sqrt (N31+N32)	0.300535105
Slope Ratio (S.R.)	6.82782492
f ratio (S.R.)	1.99772224

Test for Parallelism **0.292585452**

NOT Parallel

log (f_ED_50_1)	0.007497515	0.393772445
log (f_ED_50_2)	0.147559223	0.515841902
sqrt (Q42+Q43)	0.393772445	0.661537707
		2.476124313
Potency Ratio (P.R.)	2165.203339	3.279758773
f ratio (P.R.)	2.476124313	4.587094708

Test of Significance **874.4324054**

SIGNIFICANT at *******

874.4324054 at (p<0.05)
660.1715216 at (p<0.01)
472.0206312 at (p<0.001)

1:1

log(dose)	DOSE	EFFECT	TOTAL	DOSE CAM	DOSE PAX	%	PROBITS	Log(dose)*PROBIT	Log(dose)^2
1.30428	**20.15**	**33.68**	**100**	**20.1411**	**0.009302174**	33.7%	4.57875	5.971988861	1.701155896
1.45040	**28.21**	**42.56**	**100**	**28.19698**	**0.013022785**	42.6%	4.81240	6.979919812	2.103669112
1.60531	**40.3**	**59.09**	**100**	**40.2814**	**0.018603978**	59.1%	5.22985	8.395504596	2.577004291
1.64670	**44.33**	**67.39**	**100**	**44.30954**	**0.020464376**	67.4%	5.45070	8.975655324	2.711613418
6.00669	132.9904	202.72	400			2.0272	20.0717	30.32306859	9.093442718

N	**96**
n'	4

a	**2.4801493**		
b	**1.2935533**		
ED_16	**12.33796302**	1.091243	
ED_50	**31.22086967**	1.494445	
ED_84	**79.00353577**	1.897647	
ED_97	**178.9725549**	2.252786	
S	**2.530471976**		
f_ED_50	**1.300139305**		
N	**96**	9.797959	0.282712
CL lower	**24.01348036**		
CL upper	**40.59147979**		
ED_50	**31.22086967**		
SE(ED_50)	**4.1790194**		
ED_16	**12.33796302**		
SE(ED_16)	**1.651478238**		
ED_84	**79.00353577**		
SE(ED_84)	**10.57489148**		

ANOVA	
r^2	0.96564
Mean	5.0179
SSt	0.4673
SSreg	0.4513
SSres	0.0161
SSt	0.4673
MSreg	0.4513
MSres	0.0080
**F-test**	**56.212**

Expected

x	y=ax+b	%
1.304284	4.528372	31.9%
1.450403	4.890769	45.7%
1.605305	5.274949	60.8%
1.646698	5.37761	64.7%

		(E-O)^2/E*(100-E)	(E-O)^2/E
f ratio $S_2 = A^{2.77/sqrtN'}$	1.404911		
$A = 10^{\gamma}$	3.328575	0.0015	0.10410
$\gamma = 1.1 (\log S)^2 / \log R$	0.522258	0.0038	0.20919
R = (largest / smallest) dose	2.199956	0.0013	0.04979
log R	0.342414	0.0031	0.11069
log S	0.403202		
$(\log S)^2$	0.162571		
largest dose	44.33	χ_{LW} (Chi) ^{2}	χ (Chi) ^{2}
smallest dose	20.1504	**0.978469**	0.47377

MDA-MB-231 IC50

CAM **40.2822** μM
PAX **0.0186** μM

NON_parallel DRRCs

ISOBOLOGRAPHY	0.20	0.40	0.60	0.80	1.00	1.20	1.40	1.60	1.80	2.00	2.20	2.40	2.60
CAM	4.03	8.06	12.08	16.11	20.14	24.17	28.20	32.23	36.25	40.28	44.31	48.34	52.37
PAX	0.00	0.00	0.01	0.01	0.01	0.01	0.01	0.01	0.02	0.02	0.02	0.02	0.02
Mixture (1:1)	**4.0301**	**8.0602**	**12.0903**	**16.1203**	**20.1504**	**24.1805**	**28.2106**	**32.2407**	**36.2708**	**40.3008**	**44.3309**	**48.3610**	**52.391**

ISOBOLOGRAPHY	2.80	3.00	3.20	3.40	3.60	3.80	4.00	4.20	4.40	4.60	4.80	5.00
CAM	56.40	60.42	64.45	68.48	72.51	76.54	80.56	84.59	88.62	92.65	96.68	######
PAX	0.03	0.03	0.03	0.03	0.03	0.04	0.04	0.04	0.04	0.04	0.04	0.05
Mixture (1:1)	**56.421**	**60.451**	**64.481**	**68.511**	**72.542**	**76.572**	**80.602**	**84.632**	**88.662**	**92.692**	**96.722**	**100.752**

PAX

[uM]	ctr	0.001	0.005	0.01	0.05	0.1	0.5	1
		**14.01**	**74.8**	**90.063**	**96.074**	**96.198**	**97.101**	**98.213**
**Mean**	100	85.99	25.2	9.937	3.926	3.802	2.899	1.787
**SE**	1.351	1.407	1.707	0.4716	0.223	0.1598	0.2097	0.2252

CAM

[mM]	ctr	0.01	0.02	0.03	0.04	0.05	0.06	0.07	0.08	0.09	0.1
		**7.28**	**21.24**	**33.33**	**44.84**	**58.64**	**74.19**	**79.58**	**81.65**	**81.76**	**80.92**
**Mean**	100	92.72	78.76	66.67	55.16	41.36	25.81	20.42	18.35	18.24	19.08
**SE**	1.222	0.8752	1.309	0.7588	0.7111	0.8701	0.389	0.331	0.1354	0.1598	0.2163

MIX 1:1

	ctr	0.4	0.6	0.8	1	1.2	1.4	1.6	1.8	2	2.2
**Mean**	100	84	81.4	74.16	66.32	64.26	57.44	48.86	42.98	40.91	32.61
		**16**	**18.6**	**25.84**	**33.68**	**35.74**	**42.56**	**51.14**	**57.02**	**59.09**	**67.39**
**SE**	1.973	2.287	3.196	3.335	3.572	4.622	2.547	3.171	2.914	2.492	2.206
		8.0602	12.0903	16.1203	20.1504	24.1805	28.2106	32.2407	36.2708	40.3008	44.3309

CAM					PAX			
a	2.920320184				a	0.84982664		
b	0.312554664				b	6.470527435		
			SEM					SEM
4 probit	1.262685289	**18.31**			4 probit	-2.907095775	**0.00**	
5 probit	1.605113495	**40.282**	4.10		5 probit	-1.730385193	**0.019**	0.0084
6 probit	1.9475417	**88.62**			6 probit	-0.553674612	**0.28**	
p	2.199				q	0.783		

0	36.19			0.00	0.02
0	40.28			20.14111	0.009302
0	44.38		01:01	20.14111	0.009302
				20.14111	0.009302
0.01	0			40.28	0
0.02	0				
0.03	0				

		a	b	sum (a+b)
		20.14111453	0.009302	20.15042
01:01	proportions	0.999538363	0.000462	

0	0
48.34	0.02

01:01	ED50 mix	a	b
SEM	**27.042**	27.02936675	0.012483523
4.179	**31.221**	31.20645696	0.014412714
	**35.400**	35.38354717	0.016341905

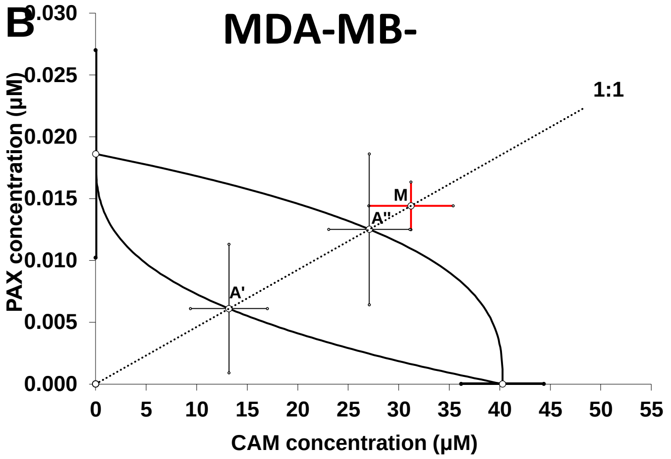

Lower

		ED50 add		SEM (a)	SEM total
X	13.2081	0.000900154	13.18651376		3.8132
Y	0.0061	0.006	13.18651376	0.0052	
		0.01	13.18651376	0.008391	
		ED50 add		SEM (b)	SUM
		0.01	9.378513757		13.19261
		0.006	13.18651376	3.808	
		0.01	16.99451376	13201.02	

Upper

		ED50 add		SEM (a)	SEM total
X	27.0825	0.006408047	27.084		4.0191
Y	0.013	0.013	27.084	0.0061	
		0.018608047	27.084	0.008389	
		ED50 add		SEM (b)	SUM
		0.01	23.071		27.09685
		0.013	27.084	4.013	
		0.013	31.097	27115.08	

ED50 mix		SEM (a)	SEM total
27.029	0.014		4.17902
31.206	0.014	4.17709	
35.384	0.014		
ED50 mix		SEM (b)	
31.206	0.012		
31.206	0.014	0.001929	
31.206	0.016		

	(a)	(b)	sum (a+b)
	20.14111453	0.009302	20.15042
proportions	0.999538363	0.000462	

109
0.00011

lower additivity	b	a	expression 1	q/p	expression 2
	0.0000	40.282			
0.0001	0.0001	39.57241492	0.993694455	2.8105444	0.982378975
0.0001	0.0002	38.87070911	0.98738891	2.8105444	0.964959239
0.0001	0.0004	38.17706989	0.981083365	2.8105444	0.947739755
0.0001	0.0005	37.4914555	0.974777821	2.8105444	0.930719485
0.0001	0.0006	36.8138241	0.968472276	2.8105444	0.913897392
0.0001	0.0007	36.14413	0.962166731	2.8105444	0.897272436
0.0001	0.0008	35.48234269	0.955861186	2.8105444	0.880843576
0.0001	0.0009	34.82840877	0.949555641	2.8105444	0.864609769
0.0001	0.0011	34.18228999	0.943250096	2.8105444	0.848569972
0.0001	0.0012	33.54394428	0.936944551	2.8105444	0.83272314
0.0001	0.0013	32.91332948	0.930639	2.8105444	0.817068227
0.0001	0.0014	32.2904034	0.924333	2.8105444	0.801604185
0.0001	0.0015	31.67512378	0.918027917	2.8105444	0.786329965
0.0001	0.0016	31.06744832	0.911722372	2.8105444	0.771244518
0.0001	0.0018	30.46733466	0.905416827	2.8105444	0.75634679
0.0001	0.0019	29.87474038	0.899111282	2.8105444	0.74163573
0.0001	0.0020	29.289623	0.892805737	2.8105444	0.727110284
0.0001	0.0021	28.71193999	0.886500192	2.8105444	0.712769394
0.0001	0.0022	28.14164878	0.880195	2.8105444	0.698612004
0.0001	0.0023	27.5787067	0.873889103	2.8105444	0.684637056
0.0001	0.0025	27.02307106	0.867583558	2.8105444	0.670843488
0.0001	0.0026	26.47469911	0.861278013	2.8105444	0.657230241
0.0001	0.0027	25.93354801	0.854972468	2.8105444	0.64379625
0.0001	0.0028	25.39957489	0.848666923	2.8105444	0.630540451
0.0001	0.0029	24.87273681	0.842361378	2.8105444	0.617461779
0.0001	0.0031	24.35299077	0.836055833	2.8105444	0.604559165
0.0001	0.0032	23.84029372	0.829750289	2.8105444	0.591831541
0.0001	0.0033	23.33460252	0.823444744	2.8105444	0.579277837
0.0001	0.0034	22.83587399	0.817139199	2.8105444	0.56689698
0.0001	0.0035	22.34406489	0.810833654	2.8105444	0.554687896
0.0001	0.0036	21.8591319	0.804528109	2.8105444	0.542649511
0.0001	0.0038	21.38103166	0.798222564	2.8105444	0.530780748
0.0001	0.0039	20.90972072	0.791917019	2.8105444	0.519080528
0.0001	0.0040	20.44515558	0.785611474	2.8105444	0.507547771
0.0001	0.0041	19.98729266	0.77930593	2.8105444	0.496181396
0.0001	0.0042	19.53608834	0.773000385	2.8105444	0.48498032
0.0001	0.0043	19.09149891	0.76669484	2.8105444	0.473943457
0.0001	0.0045	18.65348061	0.760389295	2.8105444	0.463069722
0.0001	0.0046	18.22198958	0.75408375	2.8105444	0.452358025
0.0001	0.0047	17.79698193	0.747778205	2.8105444	0.441807277
0.0001	0.0048	17.37841367	0.74147266	2.8105444	0.431416386
0.0001	0.0049	16.96624	0.735167115	2.8105444	0.421184258
0.0001	0.0050	16.56041909	0.728861571	2.8105444	0.411109799
0.0001	0.0052	16.16090446	0.722556026	2.8105444	0.401191911
0.0001	0.0053	15.7676526	0.716250481	2.8105444	0.391429495
0.0001	0.0054	15.38061919	0.709944936	2.8105444	0.381821452
0.0001	0.0055	14.99975981	0.703639391	2.8105444	0.372366678
0.0001	0.0056	14.62502997	0.697333846	2.8105444	0.363064068
0.0001	0.0057	14.25638513	0.691028301	2.8105444	0.353912518
0.0001	0.0059	13.89378063	0.684722757	2.8105444	0.344910919
0.0001	0.0060	13.53717177	0.678417212	2.8105444	0.33605816
0.0001173	0.0061	13.18651376	0.672111667	2.8105444	0.32735313
0.0002	0.0063	12.55251141	0.660431039	2.8105444	0.311614121
0.0002	0.0065	11.93848955	0.648750411	2.8105444	0.296371125
0.0002	0.0068	11.34416124	0.637069782	2.8105444	0.281617018
0.0002	0.0070	10.76923861	0.625389154	2.8105444	0.267344654
0.0002	0.0072	10.21343273	0.613708526	2.8105444	0.253546861
0.0002	0.0074	9.676453698	0.602027898	2.8105444	0.240216441
0.0002	0.0076	9.158010529	0.59034727	2.8105444	0.227346171
0.0002	0.0078	8.657811184	0.578666642	2.8105444	0.214928801
0.0002	0.0081	8.175562521	0.566986014	2.8105444	0.202957054
0.0002	0.0083	7.710970276	0.555305386	2.8105444	0.191423624
0.0002	0.0085	7.263739033	0.543624758	2.8105444	0.180321179
0.0002	0.0087	6.833572198	0.53194413	2.8105444	0.169642355
0.0002	0.0089	6.420171965	0.520263501	2.8105444	0.159379759
0.0002	0.0091	6.023239289	0.508582873	2.8105444	0.149525968
0.0002	0.0094	5.642473846	0.496902245	2.8105444	0.140073526
0.0002	0.0096	5.277574006	0.485221617	2.8105444	0.131014945
0.0002	0.0098	4.92823679	0.473540989	2.8105444	0.122342703
0.0002	0.0100	4.594157835	0.461860361	2.8105444	0.114049246
0.0002	0.0102	4.275031351	0.450179733	2.8105444	0.106126981
0.0002	0.0104	3.970550081	0.438499105	2.8105444	0.098568281
0.0002	0.0107	3.68040525	0.426818477	2.8105444	0.091365481
0.0002	0.0109	3.404286523	0.415137849	2.8105444	0.084510877
0.0002	0.0111	3.141881951	0.40345722	2.8105444	0.077996725
0.0002	0.0113	2.892877917	0.391776592	2.8105444	0.071815239
0.0002	0.0115	2.656959077	0.380095964	2.8105444	0.065958591
0.0002	0.0118	2.433808304	0.368415336	2.8105444	0.060418908
0.0002	0.0120	2.223106614	0.356734708	2.8105444	0.055188272
0.0002	0.0122	2.024533103	0.34505408	2.8105444	0.050258716
0.0002	0.0124	1.837764869	0.333373452	2.8105444	0.045622224
0.0002	0.0126	1.662476932	0.321692824	2.8105444	0.041270728
0.0002	0.0128	1.498342145	0.310012196	2.8105444	0.037196108
0.0002	0.0131	1.345031103	0.298331568	2.8105444	0.033390186
0.0002	0.0133	1.202212039	0.286650939	2.8105444	0.029844725
0.0002	0.0135	1.069550716	0.274970311	2.8105444	0.026551428
0.0002	0.0137	0.946710303	0.263289683	2.8105444	0.023501934
0.0002	0.0139	0.833351247	0.251609055	2.8105444	0.020687814
0.0002	0.0141	0.729131125	0.239928427	2.8105444	0.018100565
0.0002	0.0144	0.633704489	0.228247799	2.8105444	0.015731614
0.0002	0.0146	0.546722686	0.216567171	2.8105444	0.013572305
0.0002	0.0148	0.467833661	0.204886543	2.8105444	0.011613897
0.0002	0.0150	0.396681743	0.193205915	2.8105444	0.009847562
0.0002	0.0152	0.332907389	0.181525287	2.8105444	0.008264374
0.0002	0.0154	0.276146913	0.169844658	2.8105444	0.006855304
0.0002	0.0157	0.226032158	0.15816403	2.8105444	0.005611213
0.0002	0.0159	0.18219013	0.146483402	2.8105444	0.004522841
0.0002	0.0161	0.144242572	0.134802774	2.8105444	0.003580799
0.0002	0.0163	0.11180545	0.123122146	2.8105444	0.002775553
0.0002	0.0165	0.084488359	0.111441518	2.8105444	0.00209741
0.0002	0.0167	0.061893788	0.09976089	2.8105444	0.001536504
0.0002	0.0170	0.043616217	0.088080262	2.8105444	0.001082766
0.0002	0.0172	0.029240984	0.076399634	2.8105444	0.000725903
0.0002	0.0174	0.018342804	0.064719006	2.8105444	0.000455357
0.0002	0.0176	0.010483777	0.053038377	2.8105444	0.000260258
0.0002	0.0178	0.005210571	0.041357749	2.8105444	0.000129352
0.0002	0.0181	0.002050157	0.029677121	2.8105444	5.08948E-05
0.0002	0.0183	0.000502622	0.017996493	2.8105444	1.24775E-05
	0.0186	0			

upper additivity	b	a	expression 1	q/p	expression 2	expression 3
	0.00	40.282				
0.000	0.000	40.282	62.47044328	2.8105444	111384.0663	0.000361652
0.000	0.001	40.280	31.23522164	2.8105444	15876.87095	0.002537164
0.000	0.001	40.274	20.82348109	2.8105444	5079.870269	0.007929775
0.000	0.001	40.264	15.61761082	2.8105444	2263.115718	0.017799456
0.000	0.001	40.249	12.49408866	2.8105444	1208.751069	0.033325496
0.000	0.002	40.227	10.41174055	2.8105444	724.0931975	0.055631277
0.000	0.002	40.196	8.92434904	2.8105444	469.5021075	0.08579776
0.000	0.002	40.157	7.80880541	2.8105444	322.5882965	0.124871948
0.000	0.003	40.108	6.941160364	2.8105444	231.6766016	0.173872669
0.000	0.003	40.048	6.247044328	2.8105444	172.2973975	0.233794762
0.000	0.003	39.977	5.679131207	2.8105444	131.8082943	0.305612247
0.000	0.004	39.892	5.205870273	2.8105444	103.2134545	0.390280795
0.000	0.004	39.793	4.805418714	2.8105444	82.42061909	0.488739705
0.000	0.004	39.680	4.46217452	2.8105444	66.92361503	0.601913526
0.000	0.004	39.552	4.164696219	2.8105444	55.12726214	0.730713398
0.000	0.005	39.406	3.904402705	2.8105444	45.98227488	0.876038195
0.000	0.005	39.243	3.674731958	2.8105444	38.77857119	1.038775484
0.000	0.005	39.062	3.470580182	2.8105444	33.02356997	1.219802375
0.000	0.006	38.862	3.287918067	2.8105444	28.36804185	1.419986239
0.000	0.006	38.642	3.123522164	2.8105444	24.55955897	1.64018536
0.000	0.006	38.401	2.974783013	2.8105444	21.41248634	1.881249493
0.000	0.007	38.138	2.839565604	2.8105444	18.78817455	2.144020376
0.000	0.007	37.853	2.716106229	2.8105444	16.58160599	2.429332182
0.000	0.007	37.544	2.602935137	2.8105444	14.71221829	2.738011922
0.000	0.007	37.211	2.498817731	2.8105444	13.11748795	3.070879821
0.000	0.008	36.853	2.402709357	2.8105444	11.74837278	3.42874965
0.000	0.008	36.470	2.313720121	2.8105444	10.56602726	3.81242903
0.000	0.008	36.060	2.23108726	2.8105444	9.539403927	4.222719719
0.000	0.009	35.622	2.154153216	2.8105444	8.643480103	4.660417863
0.000	0.009	35.156	2.082348109	2.8105444	7.857932073	5.126314238
0.000	0.009	34.661	2.01517559	2.8105444	7.166133336	5.621194468
0.000	0.010	34.136	1.952201352	2.8105444	6.554390306	6.145839229
0.000	0.010	33.581	1.893043736	2.8105444	6.01135385	6.70102444
0.000	0.010	32.995	1.837365979	2.8105444	5.527562343	7.287521436
0.000	0.010	32.376	1.784869808	2.8105444	5.095084006	7.906097136
0.000	0.011	31.725	1.735290091	2.8105444	4.707234852	8.557514196

0.000	0.011	31.040	1.688390359	2.8105444	4.358354697	9.242531153
0.000	0.011	30.320	1.643959034	2.8105444	4.043628094	9.961902562
0.000	0.012	29.566	1.601806238	2.8105444	3.758940273	10.71637912
0.000	0.012	28.776	1.561761082	2.8105444	3.500760579	11.5067078
0.000	0.012	27.949	1.523669348	2.8105444	3.266047605	12.33363194
0.000	**0.013**	**27.084**	**1.487391507**	**2.8105444**	**3.052171587**	**13.19789138**
0.000	0.013	26.852	1.478195876	2.8105444	2.999433762	13.42994454
0.000	0.013	26.618	1.469113249	2.8105444	2.947923851	13.66460977
0.000	0.013	26.380	1.460141555	2.8105444	2.89760598	13.90190017
0.000	0.013	26.140	1.451278774	2.8105444	2.848445534	14.14182879
0.000	0.013	25.898	1.442522935	2.8105444	2.800409105	14.38440869
0.000	0.013	25.653	1.433872113	2.8105444	2.753464442	14.6296529
0.000	0.013	25.405	1.425324431	2.8105444	2.707580406	14.87757445
0.000	0.013	25.154	1.416878055	2.8105444	2.662726924	15.12818634
0.000	0.013	24.901	1.408531194	2.8105444	2.618874946	15.38150156
0.000	0.013	24.645	1.400282101	2.8105444	2.575996408	15.63753309
0.000	0.013	24.386	1.392129067	2.8105444	2.534064187	15.8962939
0.000	0.013	24.124	1.384070424	2.8105444	2.493052069	16.15779693
0.000	0.014	23.860	1.376104543	2.8105444	2.452934714	16.42205512
0.000	0.014	23.593	1.368229831	2.8105444	2.413687617	16.68908138
0.000	0.014	23.323	1.360444731	2.8105444	2.375287081	16.95888863
0.000	0.014	23.051	1.352747723	2.8105444	2.337710183	17.23148975
0.000	0.014	22.775	1.34513732	2.8105444	2.300934747	17.50689763
0.000	0.014	22.497	1.337612069	2.8105444	2.264939312	17.78512512
0.000	0.014	22.216	1.330170548	2.8105444	2.229703109	18.06618509
0.000	0.014	21.932	1.322811367	2.8105444	2.195206033	18.35009036
0.000	0.014	21.645	1.315533168	2.8105444	2.161428618	18.63685376
0.000	0.014	21.356	1.308334621	2.8105444	2.128352014	18.9264881
0.000	0.014	21.063	1.301214425	2.8105444	2.095957965	19.21900618
0.000	0.014	20.768	1.294171309	2.8105444	2.064228784	19.51442077
0.000	0.014	20.469	1.287204027	2.8105444	2.033147338	19.81274466
0.000	0.015	20.168	1.280311362	2.8105444	2.002697022	20.11399059
0.000	0.015	19.864	1.27349212	2.8105444	1.972861743	20.41817132
0.000	0.015	19.557	1.266745136	2.8105444	1.943625902	20.72529957
0.000	0.015	19.247	1.260069265	2.8105444	1.914974373	21.03538806
0.000	0.015	18.934	1.253463391	2.8105444	1.886892491	21.3484495
0.000	0.015	18.618	1.246926418	2.8105444	1.859366033	21.66449658
0.000	0.015	18.299	1.240457274	2.8105444	1.832381201	21.98354198
0.000	0.015	17.977	1.234054908	2.8105444	1.805924611	22.30559837
0.000	0.015	17.652	1.227718291	2.8105444	1.779983275	22.63067841
0.000	0.015	17.323	1.221446417	2.8105444	1.754544589	22.95879473
0.000	0.015	16.992	1.215238297	2.8105444	1.72959632	23.28995997
0.000	0.015	16.658	1.209092965	2.8105444	1.705126593	23.62418675
0.000	0.015	16.321	1.203009473	2.8105444	1.681123878	23.96148767
0.000	0.016	15.980	1.196986891	2.8105444	1.657576978	24.30187533
0.000	0.016	15.637	1.191024311	2.8105444	1.63447502	24.64536231
0.000	0.016	15.290	1.18512084	2.8105444	1.611807444	24.99196118
0.000	0.016	14.941	1.179275602	2.8105444	1.589563987	25.3416845
0.000	0.016	14.588	1.173487741	2.8105444	1.567734683	25.69454482
0.000	0.016	14.232	1.167756416	2.8105444	1.546309842	26.05055467
0.000	0.016	13.873	1.162080803	2.8105444	1.52528005	26.40972657
0.000	0.016	13.510	1.156460093	2.8105444	1.504636156	26.77207304
0.000	0.016	13.145	1.150893493	2.8105444	1.484369263	27.13760658
0.000	0.016	12.776	1.145380226	2.8105444	1.46447072	27.50633967
0.000	0.016	12.404	1.139919529	2.8105444	1.444932117	27.8782848
0.000	0.016	12.029	1.134510653	2.8105444	1.425745272	28.25345442
0.000	0.016	11.650	1.129152865	2.8105444	1.406902229	28.63186101
0.000	0.017	11.269	1.123845444	2.8105444	1.388395246	29.01351699
0.000	0.017	10.884	1.118587683	2.8105444	1.370216794	29.3984348
0.000	0.017	10.496	1.113378888	2.8105444	1.352359542	29.78662686
0.000	0.017	10.104	1.108218379	2.8105444	1.33481636	30.17810559
0.000	0.017	9.709	1.103105487	2.8105444	1.317580307	30.57288338
0.000	0.017	9.311	1.098039557	2.8105444	1.300644625	30.97097262
0.000	0.017	8.910	1.093019943	2.8105444	1.284002736	31.37238569
0.000	0.017	8.505	1.088046014	2.8105444	1.267648236	31.77713496
0.000	0.017	8.097	1.083117149	2.8105444	1.251574887	32.18523278
0.000	0.017	7.686	1.078232739	2.8105444	1.235776615	32.5966915
0.000	0.017	7.271	1.073392184	2.8105444	1.220247503	33.01152345
0.000	0.017	6.852	1.068594896	2.8105444	1.204981789	33.42974096
0.000	0.017	6.431	1.063840299	2.8105444	1.189973857	33.85135635
0.000	0.018	6.006	1.059127824	2.8105444	1.175218235	34.27638191
0.000	0.018	5.577	1.054456915	2.8105444	1.160709594	34.70482994
	0.019	0.000				
