# Supplementary material for: Additive pharmacological interaction between sirtuin inhibitor cambinol and paclitaxel in MCF7 luminal and MDA-MB-231 triple-negative breast cancer cells
Source: Pharmacol Rep. 2022 Jul 28;74(5):1011–24. doi: 10.1007/s43440-022-00393-w (PMC9585000; doi:10.1007/s43440-022-00393-w)
Supplement: Supplementary file 13 — Supplementary file13 (PDF 337 kb) [file 43440_2022_393_MOESM13_ESM.pdf]

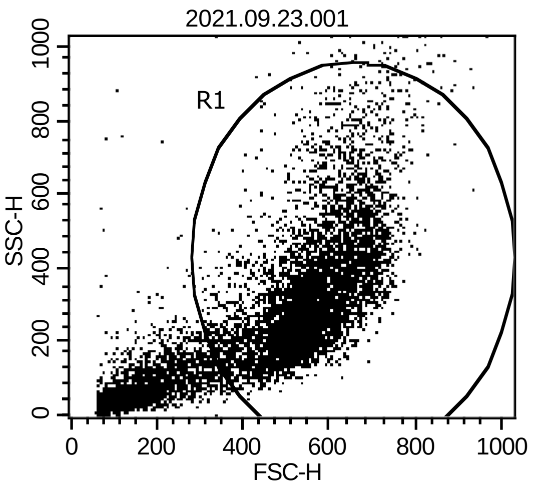

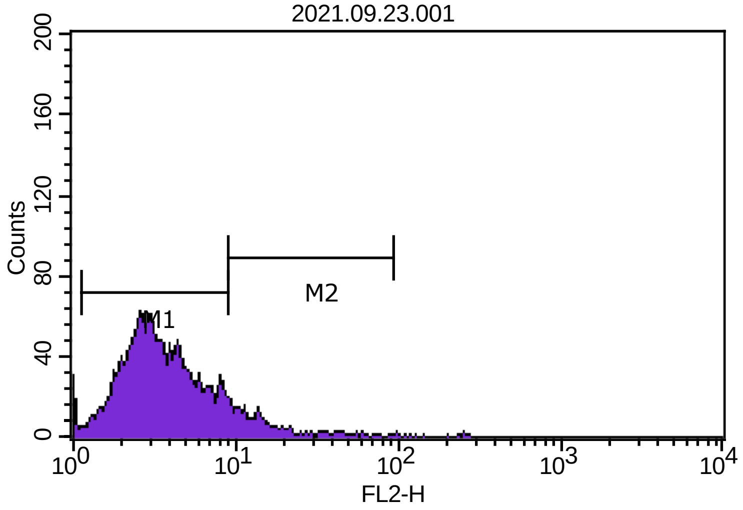

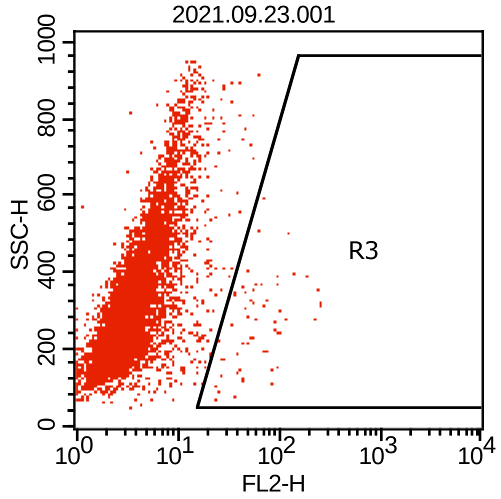

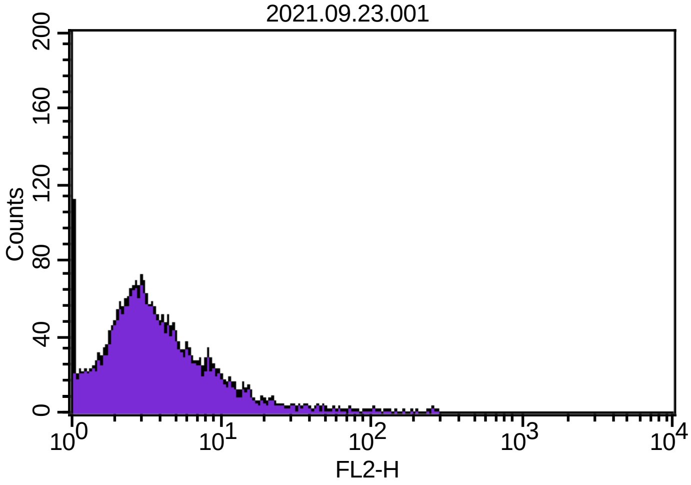

Region Statistics

File: 2021.09.23.001

Sample ID: MCF7 CTR I

Acquisition Date: 23-Sep-21

Region	% Gated
R1	100.00
R3	2.52
R2	94.83

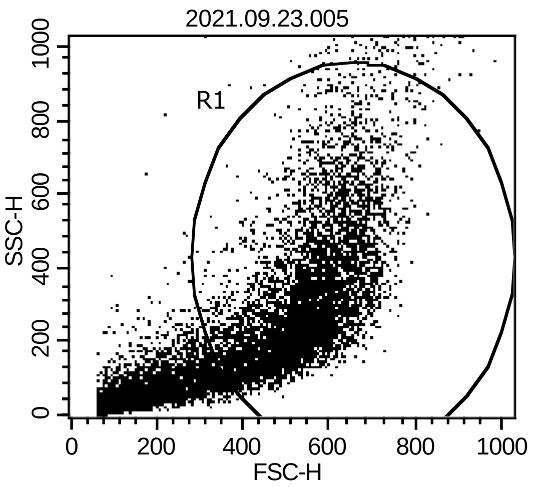

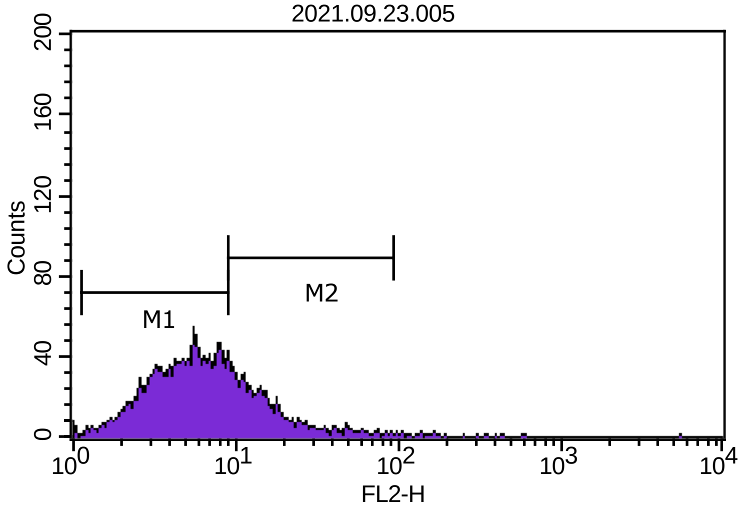

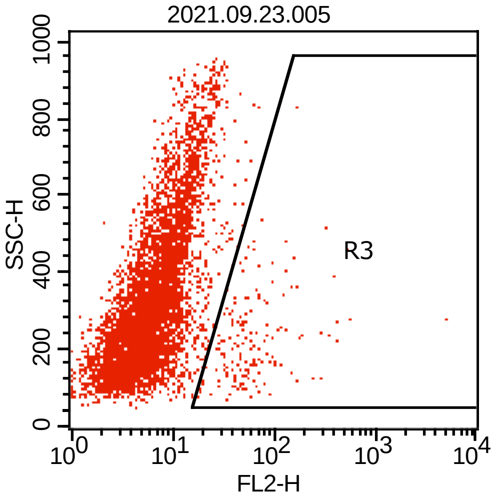

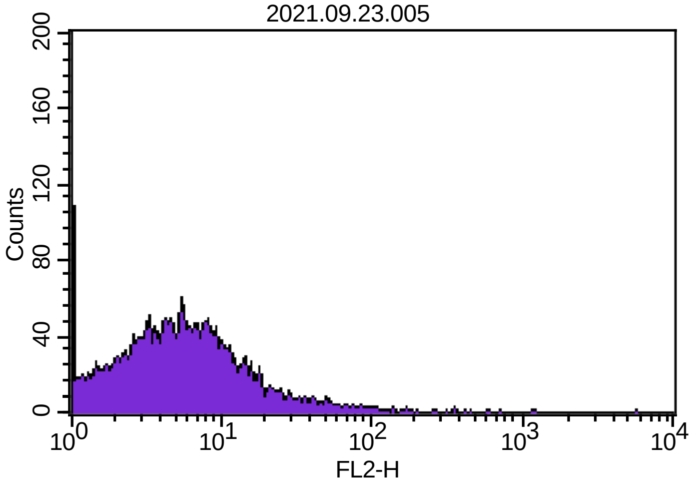

Region Statistics

File: 2021.09.23.005 Sample ID: MCF7 PAX IC50 II

Acquisition Date: 23-Sep-21

Region	% Gated
R1	100.00
R3	4.31
R2	90.99

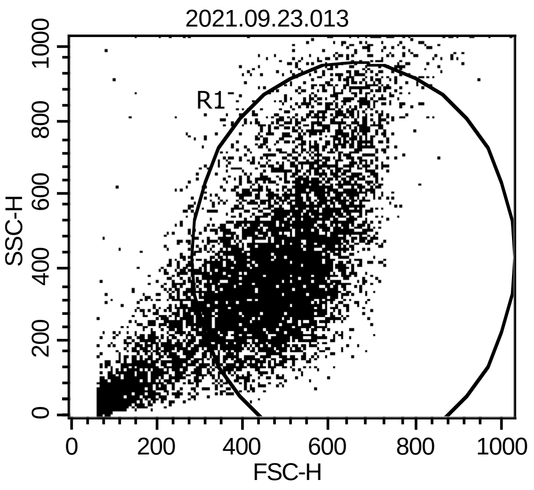

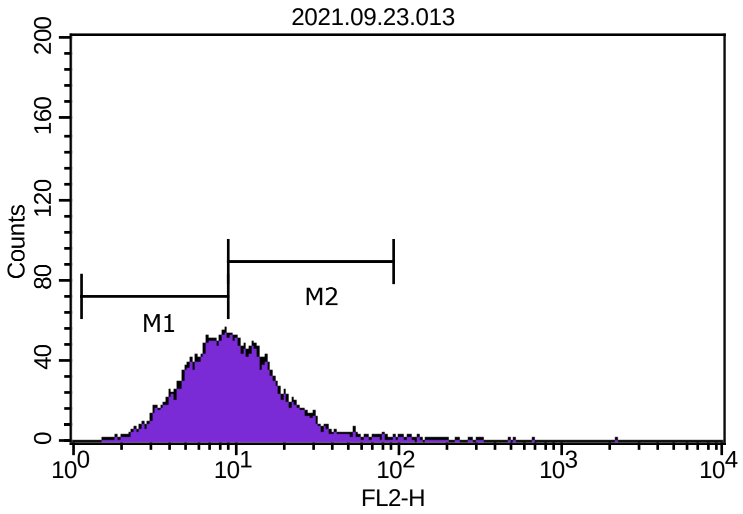

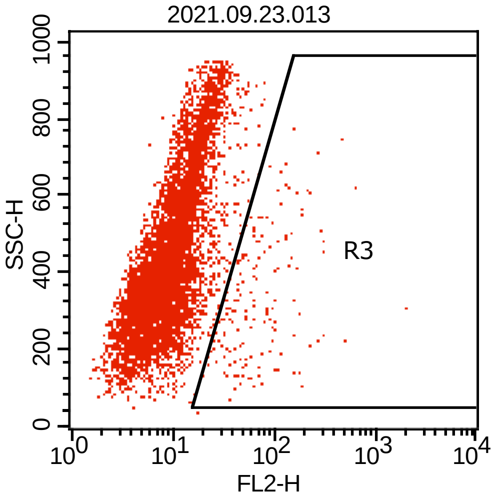

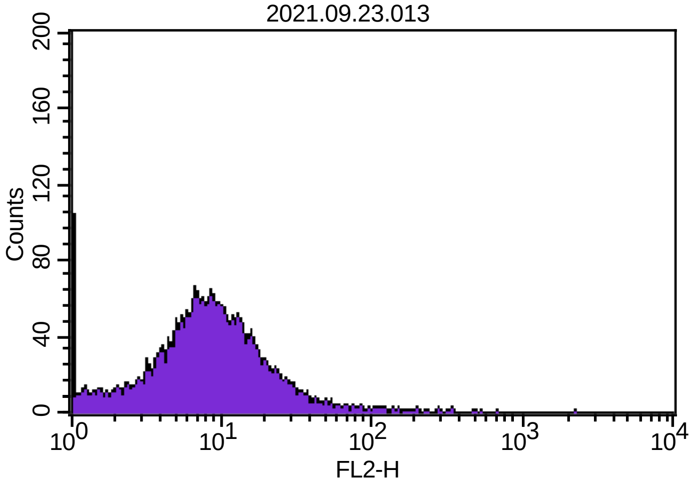

Region Statistics

File: 2021.09.23.013   Sample ID: MCF7 PAX 2IC50 I

Acquisition Date: 23-Sep-21

Region	% Gated
R1	100.00
R3	6.59
R2	81.29

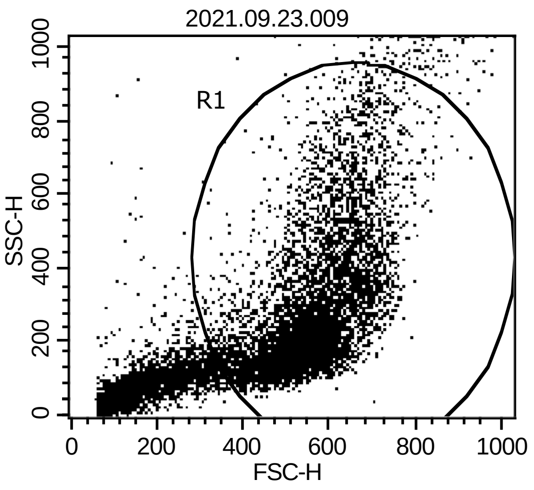

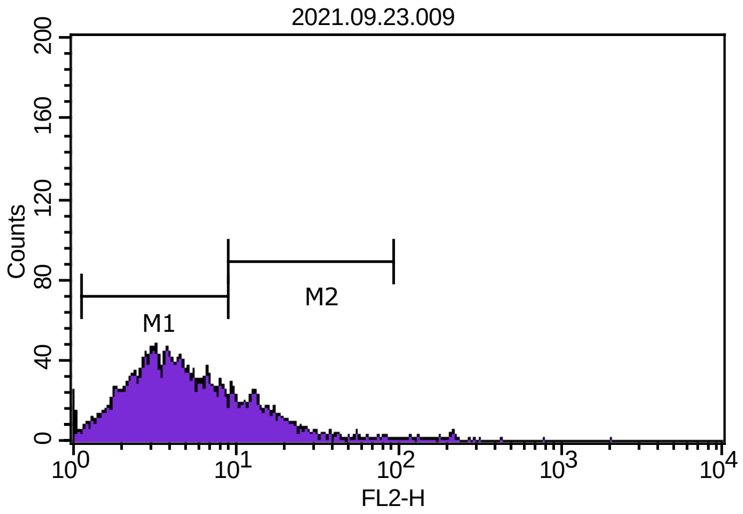

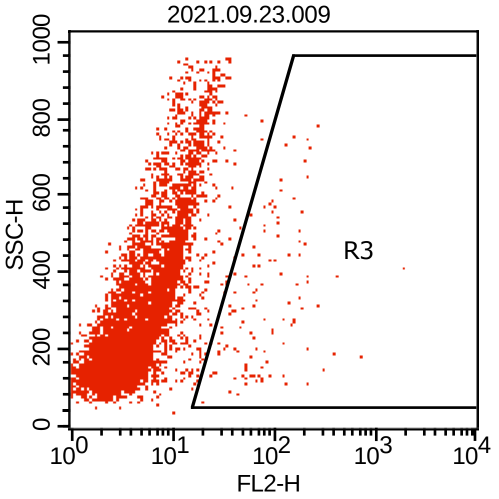

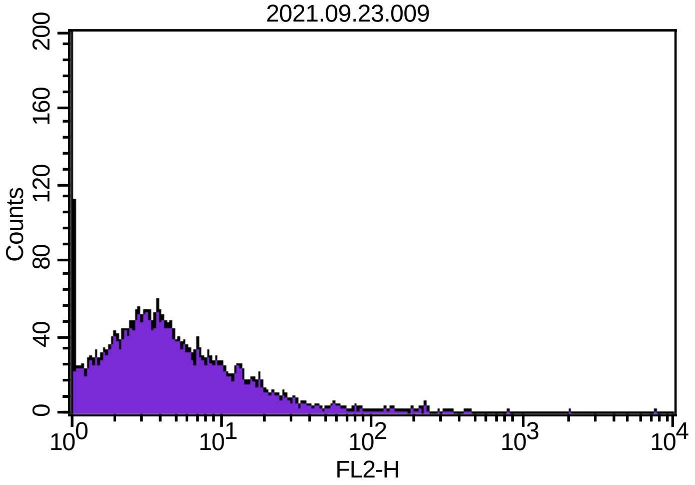

Region Statistics

File: 2021.09.23.009 Sample ID: MCF7 CAM IC50III

Acquisition Date: 23-Sep-21

Region	% Gated
R1	100.00
R3	3.88
R2	90.70

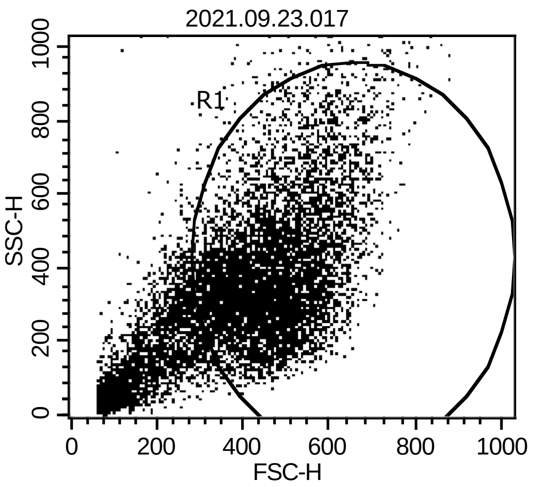

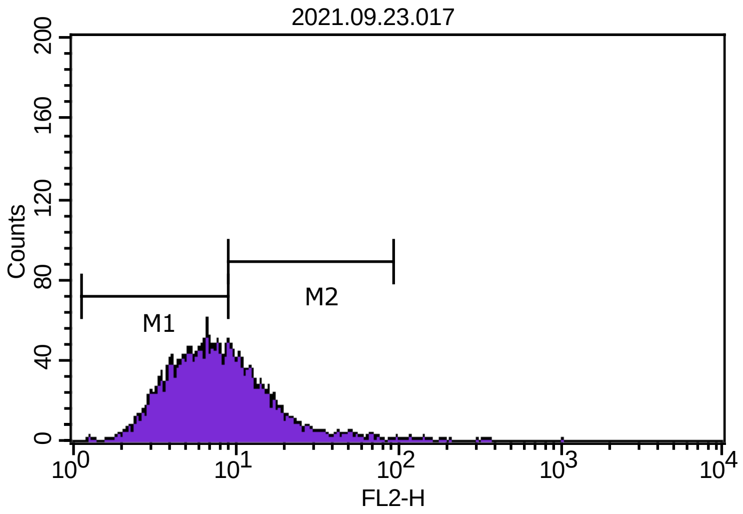

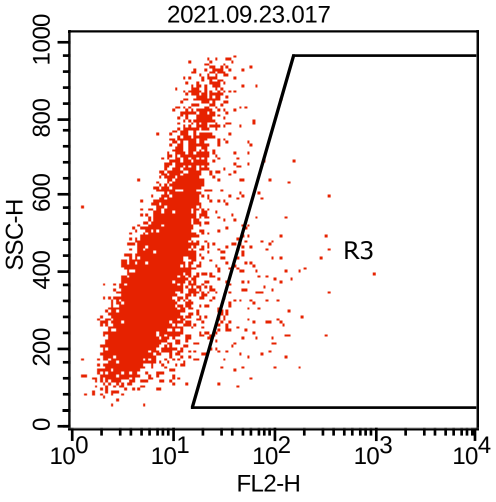

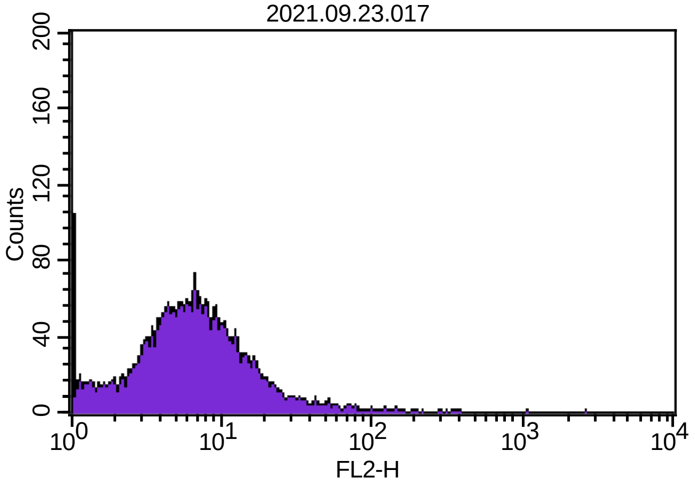

Region Statistics

File: 2021.09.23.017 Sample ID: MCF7 CAM 2IC50 II

Acquisition Date: 23-Sep-21

Region	% Gated
R1	100.00
R3	4.03
R2	74.84

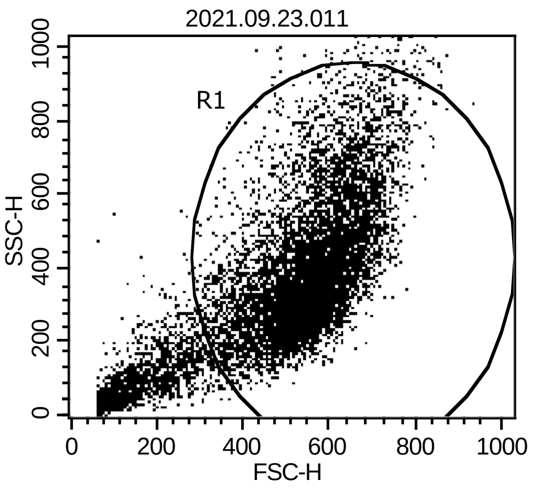

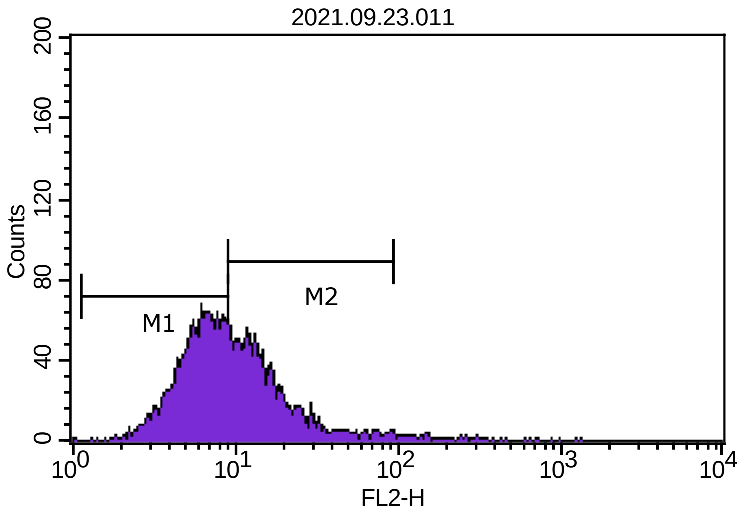

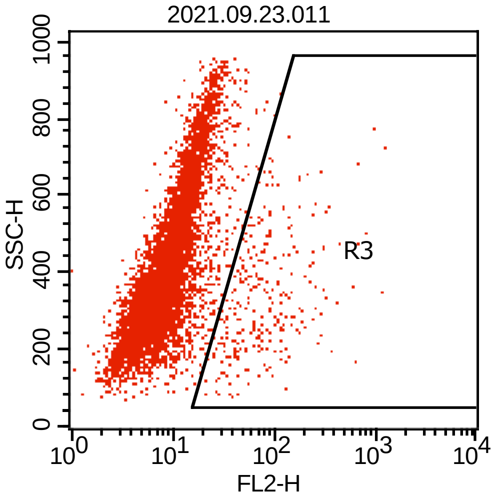

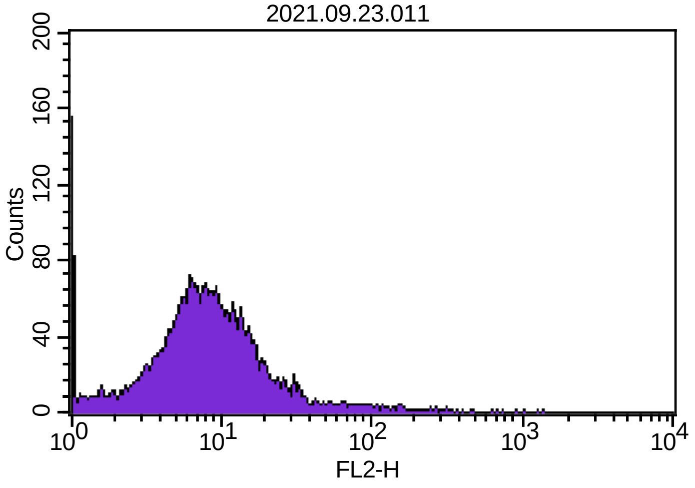

Region Statistics

File: 2021.09.23.011   Sample ID: MCF7 MIX IC50 II
Acquisition Date: 23-Sep-21

Region	% Gated
R1	100.00
R3	5.16
R2	94.46

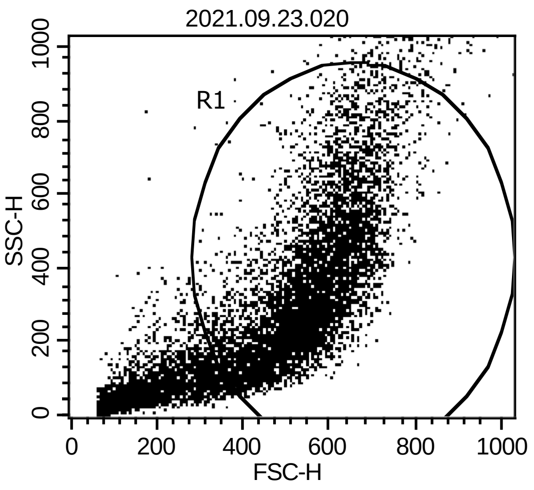

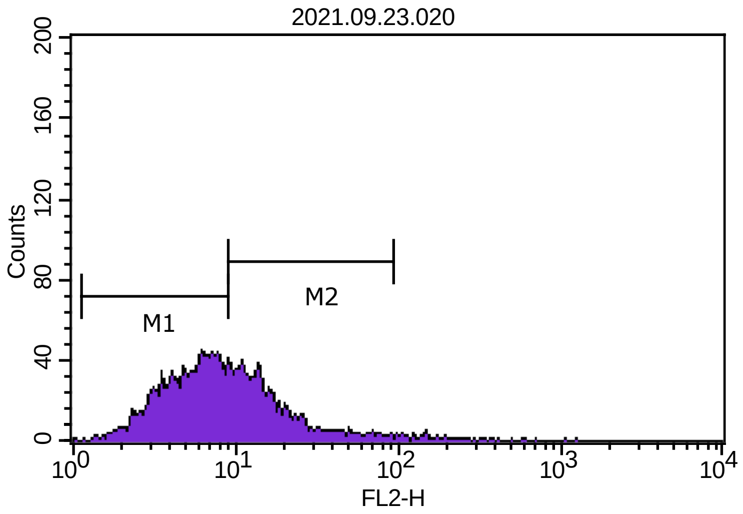

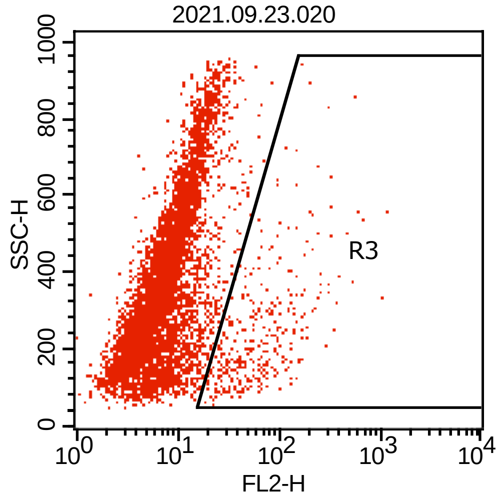

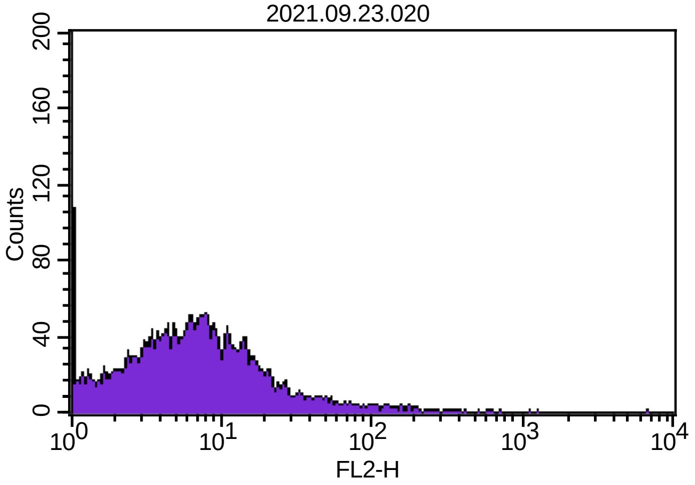

Region Statistics

File: 2021.09.23.020
Sample ID: MCF7 MIX 2IC50 II
Acquisition Date: 23-Sep-21

Region	% Gated
R1	100.00
R3	9.08
R2	90.29
