# Supplementary material for: Additive pharmacological interaction between sirtuin inhibitor cambinol and paclitaxel in MCF7 luminal and MDA-MB-231 triple-negative breast cancer cells
Source: Pharmacol Rep. 2022 Jul 28;74(5):1011–24. doi: 10.1007/s43440-022-00393-w (PMC9585000; doi:10.1007/s43440-022-00393-w)
Supplement: Supplementary file 14 — Supplementary file14 (PDF 335 kb) [file 43440_2022_393_MOESM14_ESM.pdf]

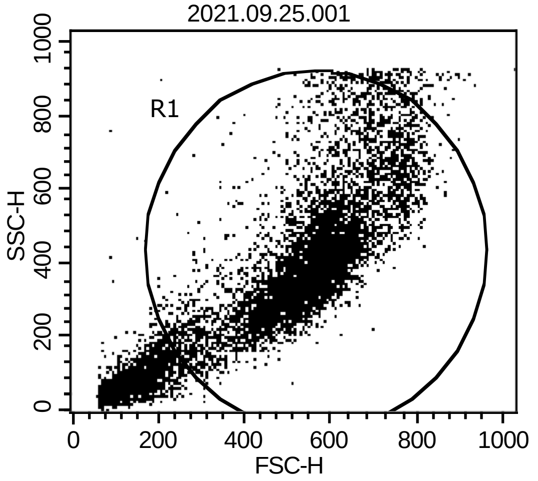

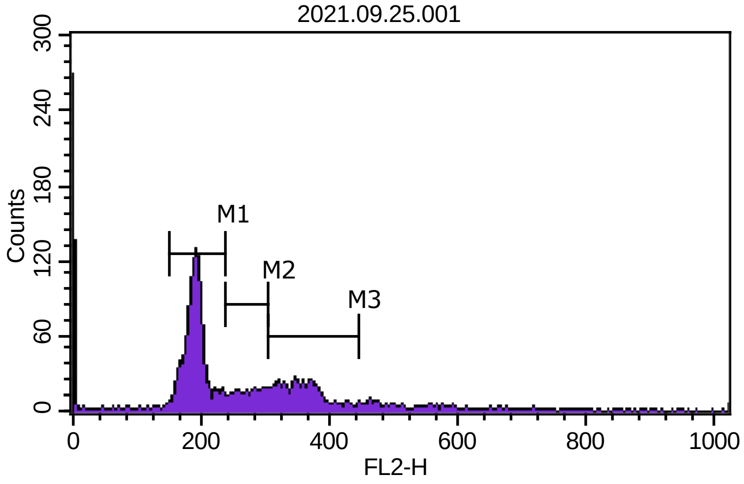

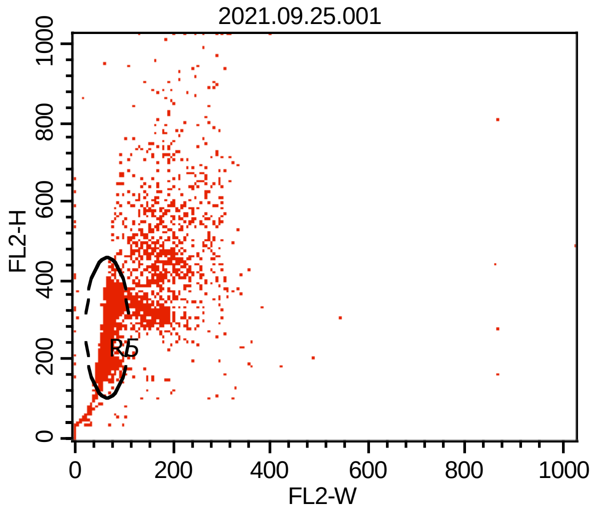

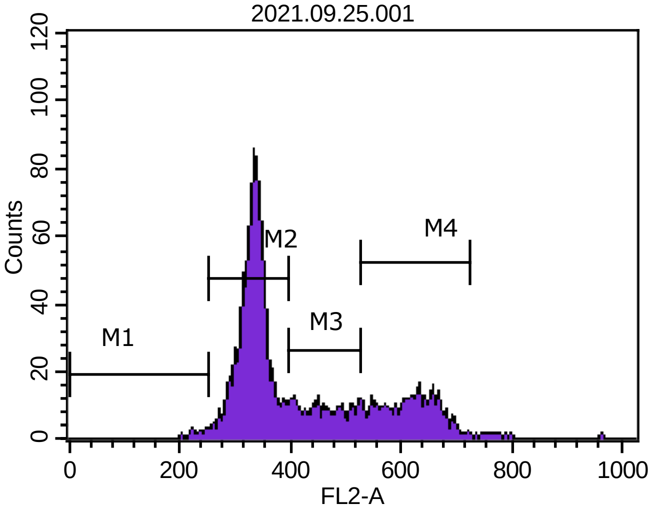

File: 2021.09.25.001 Sample ID: MCF7 Ctr I

Marker	% Gated
All	100.00
M1	0.56
M2	62.45
M3	15.04
M4	21.89

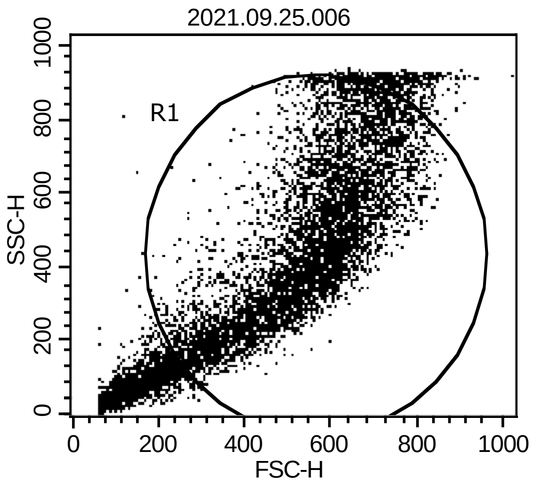

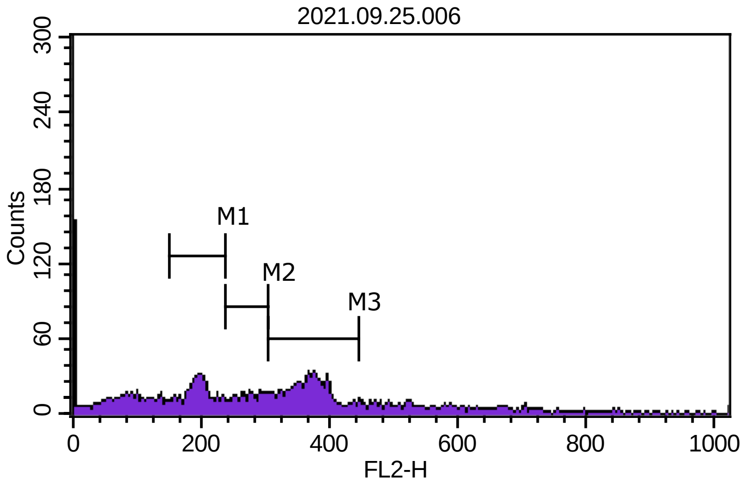

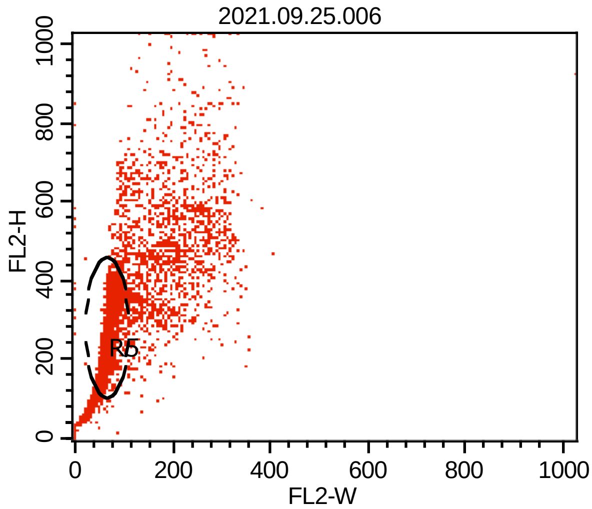

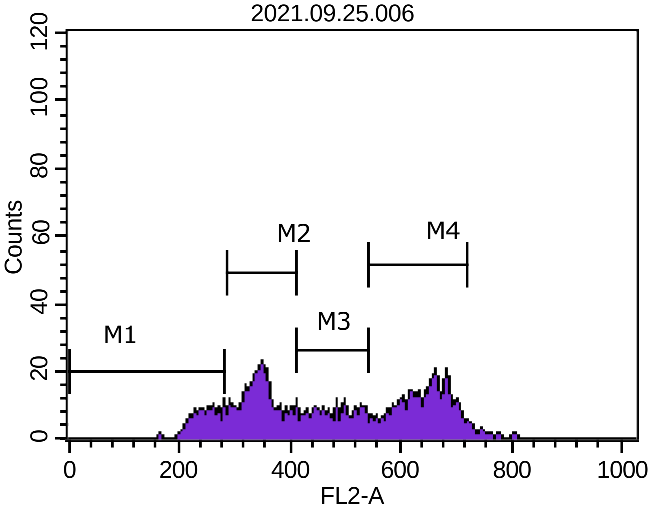

File: 2021.09.25.006 Sample ID: MCF7 PAX IC50 II

Marker	% Gated
All	100.00
M1	9.98
M2	30.16
M3	18.49
M4	40.14

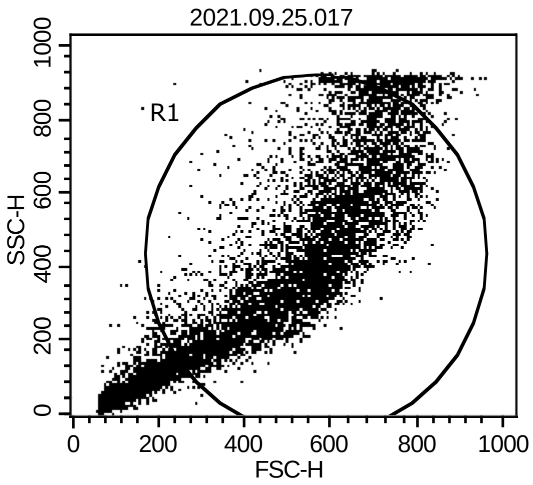

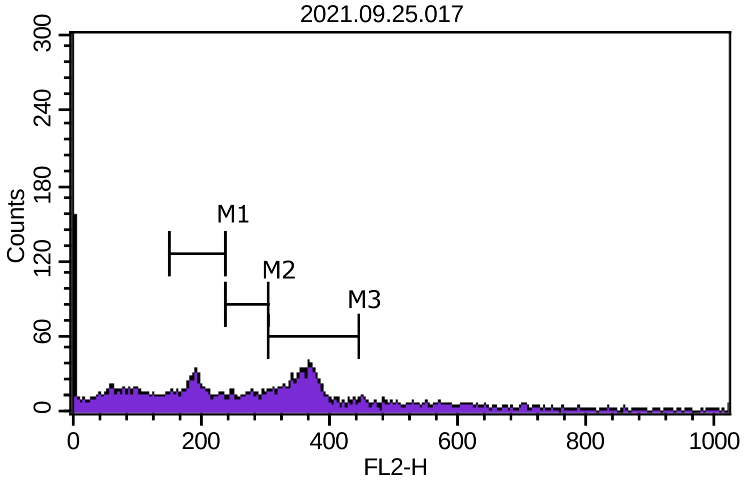

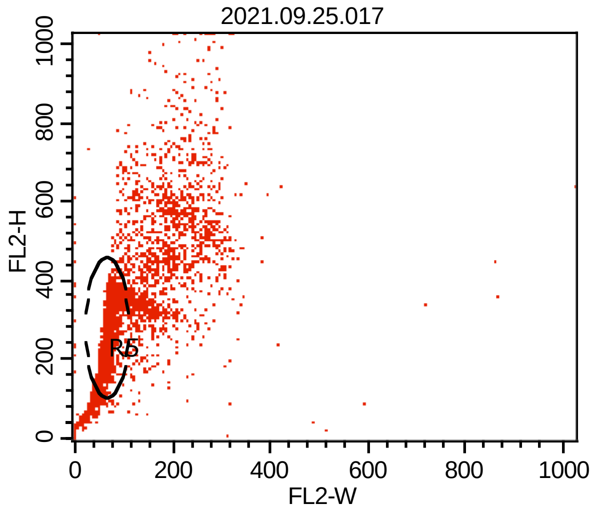

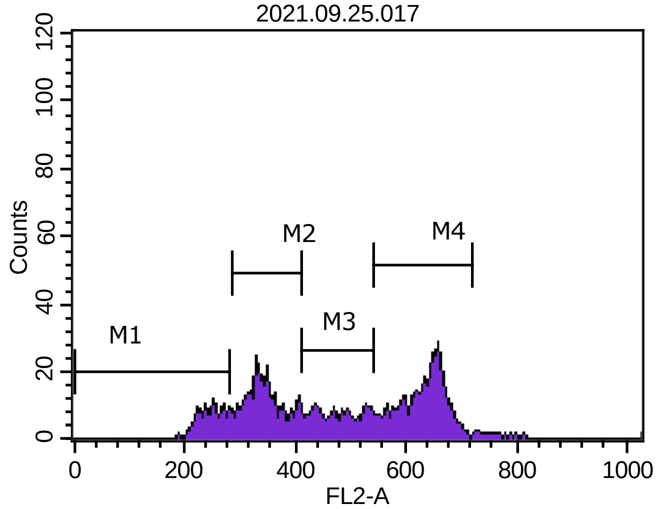

File: 2021.09.25.017 Sample ID: MCF7 PAX 2IC50 I

Marker	% Gated
All	100.00
M1	11.31
M2	30.06
M3	18.46
M4	39.19

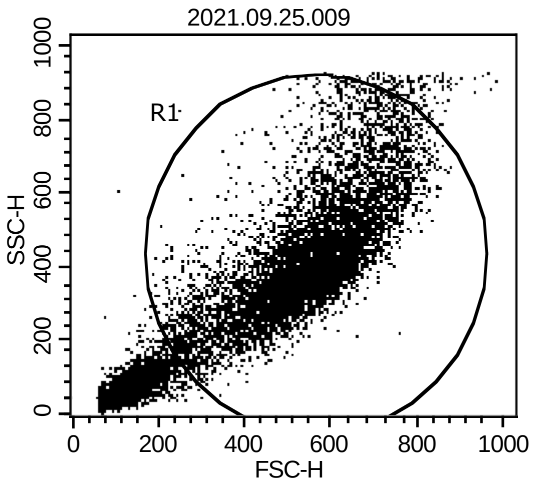

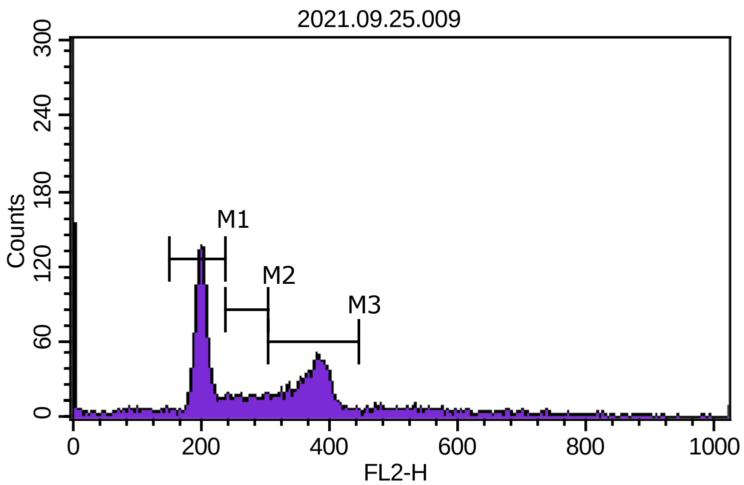

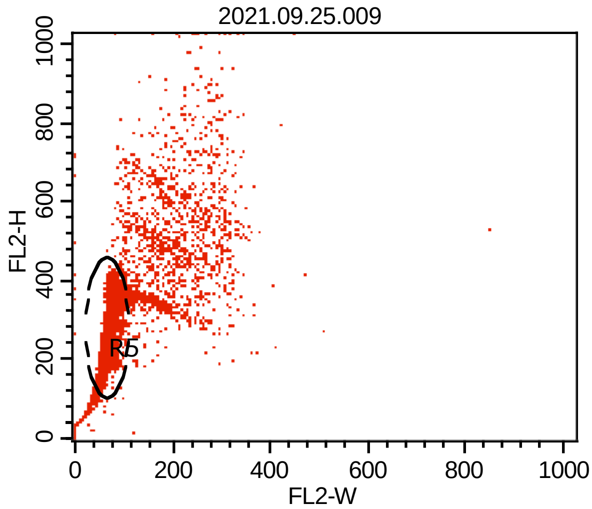

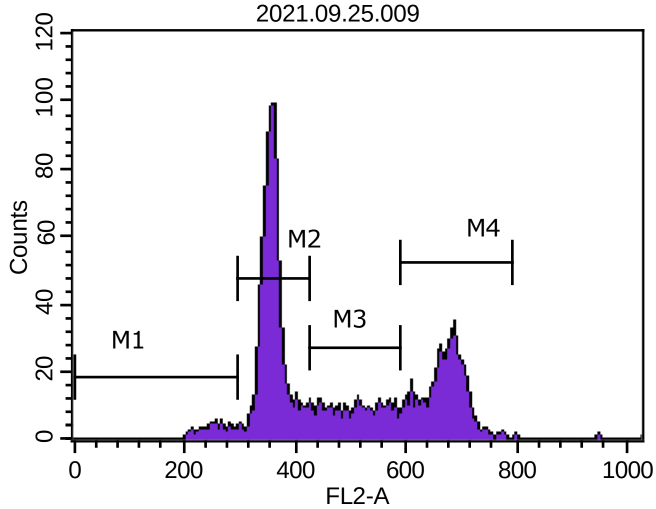

File: 2021.09.25.009 Sample ID: MCF7 CAM IC50 I

Marker	% Gated
All	100.00
M1	2.59
M2	51.46
M3	17.08
M4	29.06

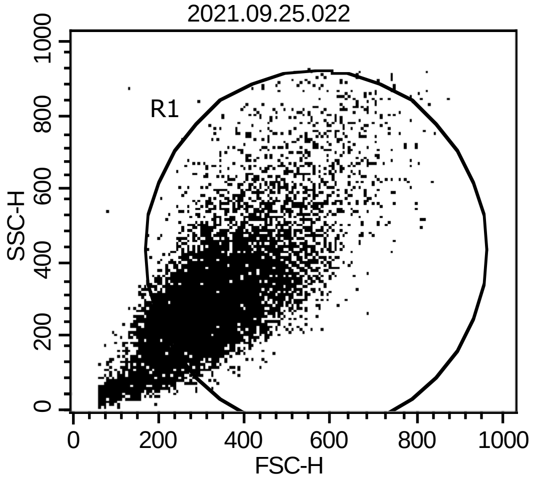

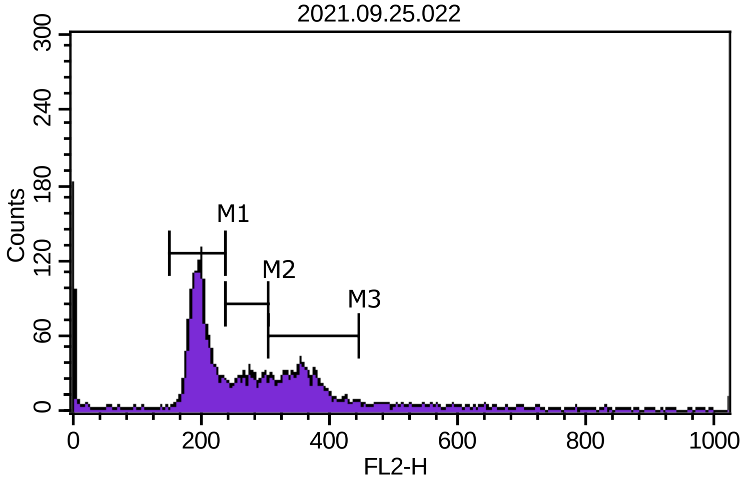

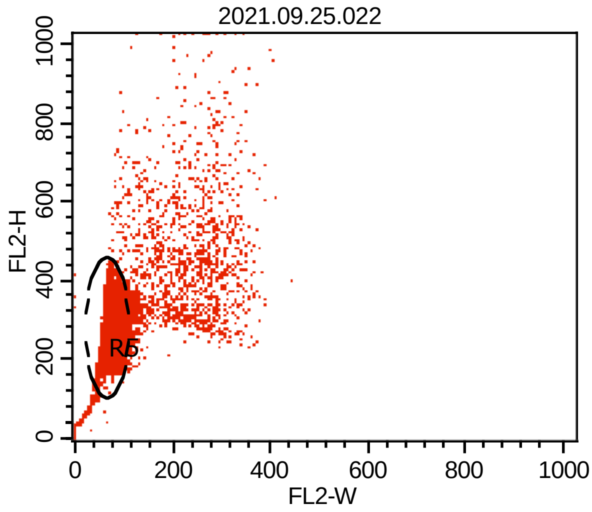

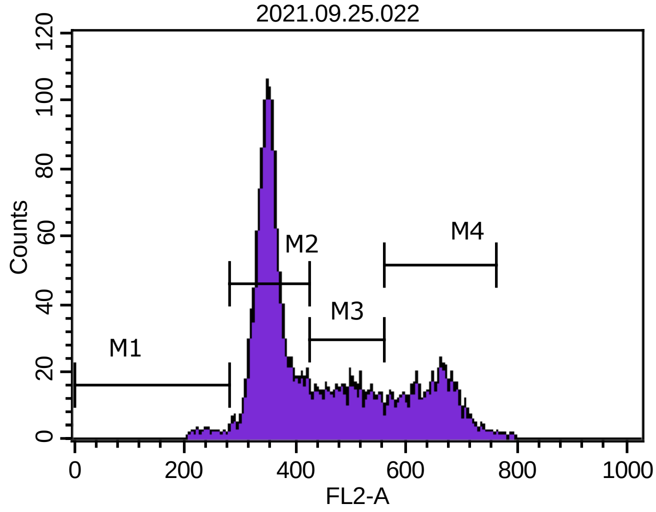

File: 2021.09.25.022 Sample ID: MCF7 CAM 2IC50 II

Marker	% Gated
All	100.00
M1	0.84
M2	60.68
M3	18.08
M4	20.61

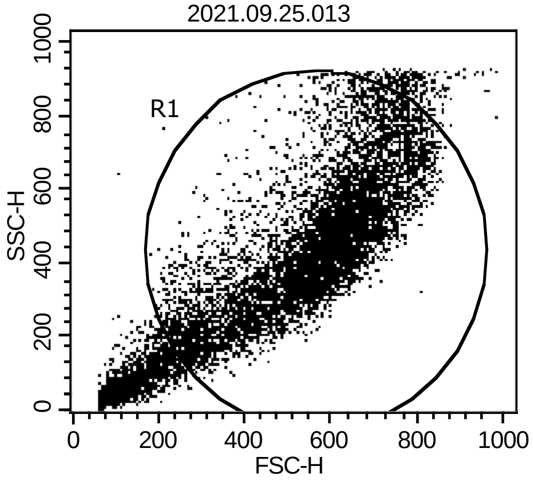

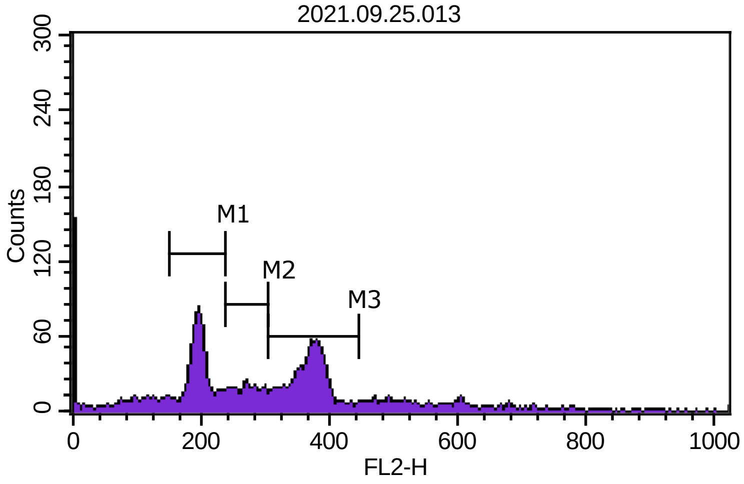

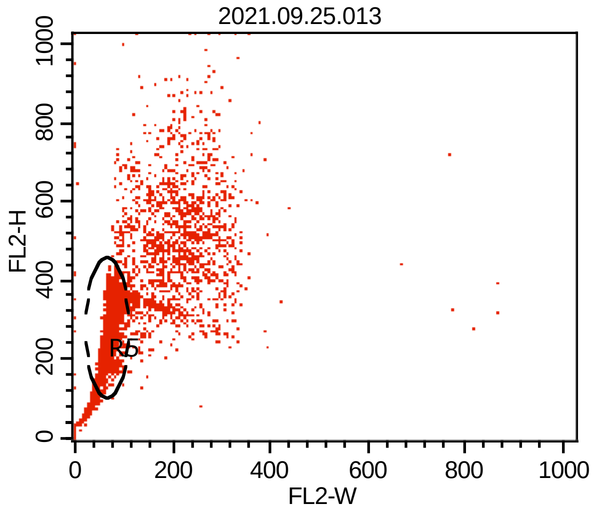

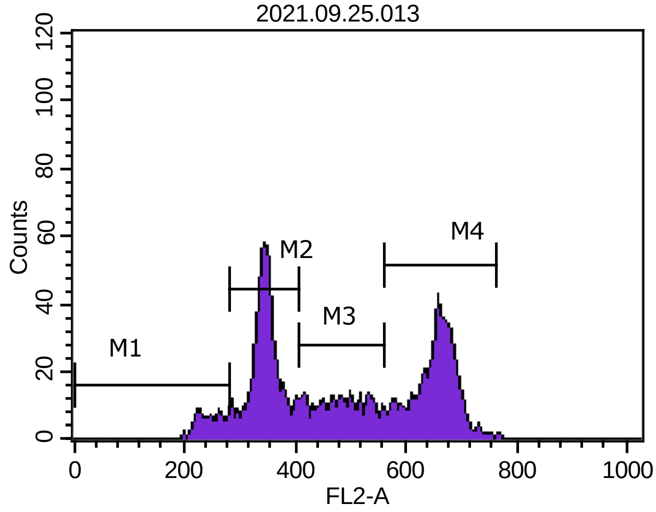

File: 2021.09.25.013 Sample ID: MCF7 MIX IC50 I

Marker	% Gated
All	100.00
M1	5.18
M2	37.71
M3	19.81
M4	37.69

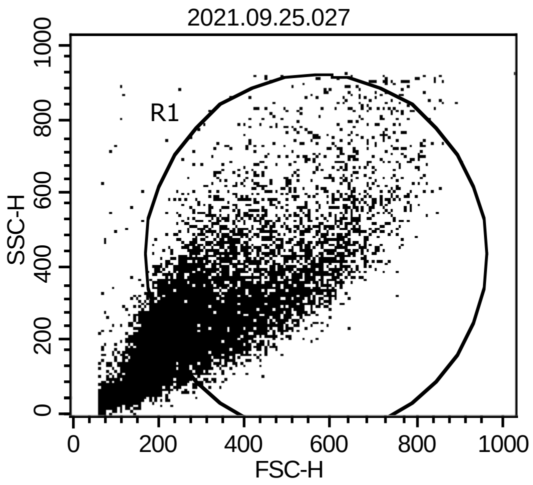

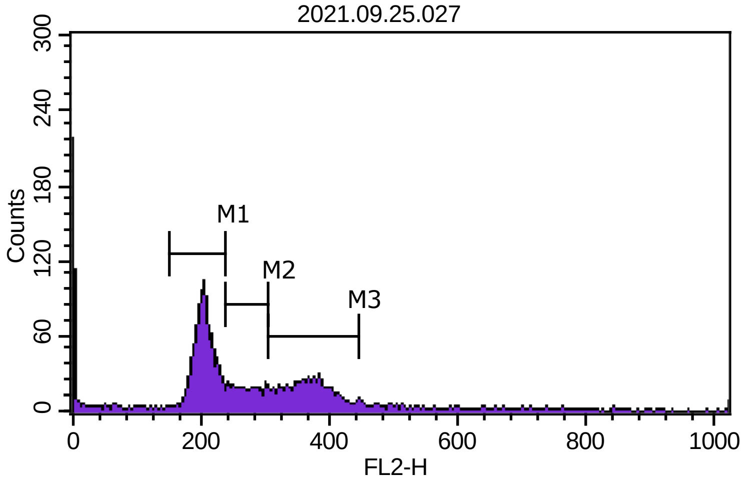

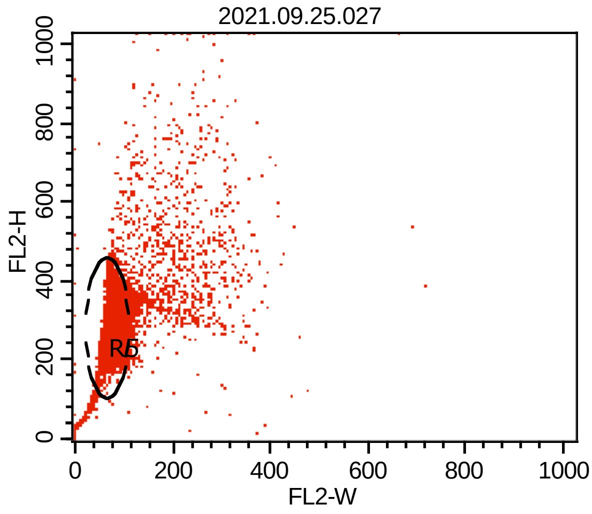

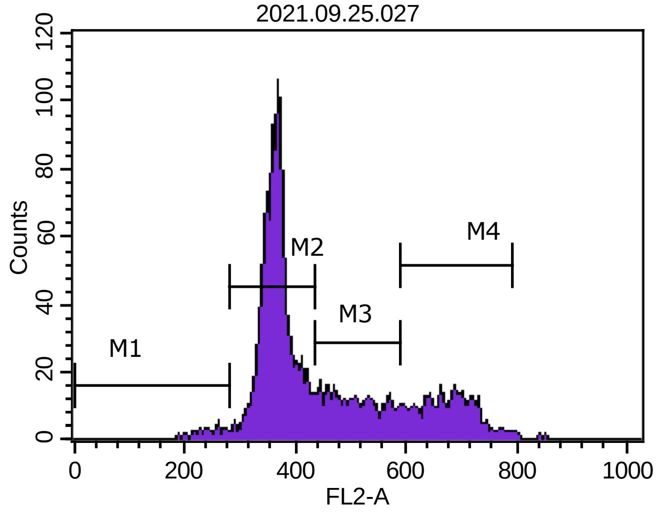

File: 2021.09.25.027 Sample ID: MCF7 MIX 2IC50 III

Marker	% Gated
All	100.00
M1	1.20
M2	62.28
M3	18.95
M4	17.82
