# Supplementary material for: Additive pharmacological interaction between sirtuin inhibitor cambinol and paclitaxel in MCF7 luminal and MDA-MB-231 triple-negative breast cancer cells
Source: Pharmacol Rep. 2022 Jul 28;74(5):1011–24. doi: 10.1007/s43440-022-00393-w (PMC9585000; doi:10.1007/s43440-022-00393-w)
Supplement: Supplementary file 15 — Supplementary file15 (PDF 318 kb) [file 43440_2022_393_MOESM15_ESM.pdf]

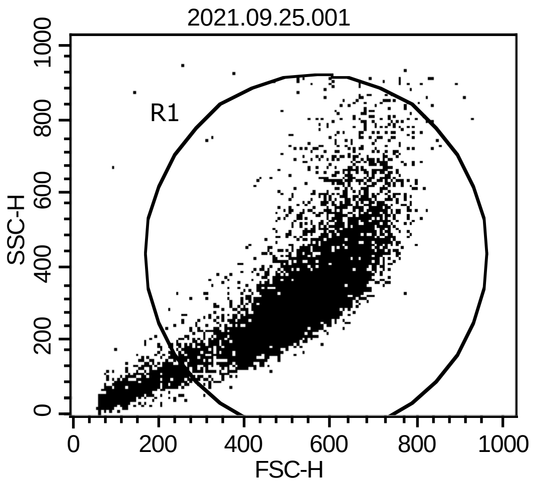

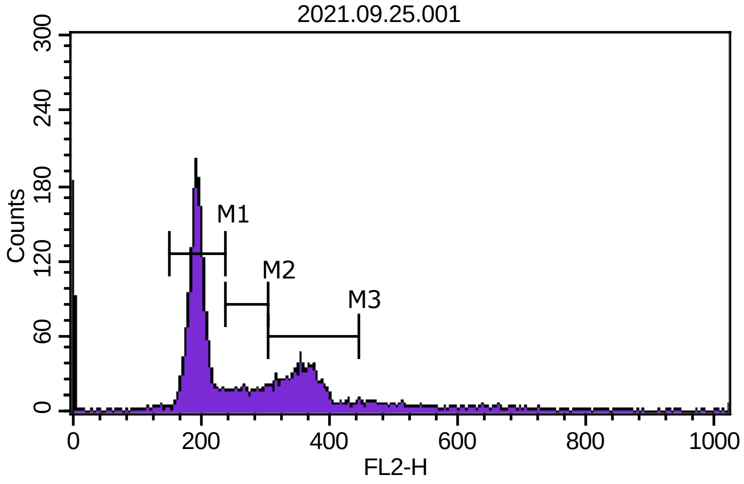

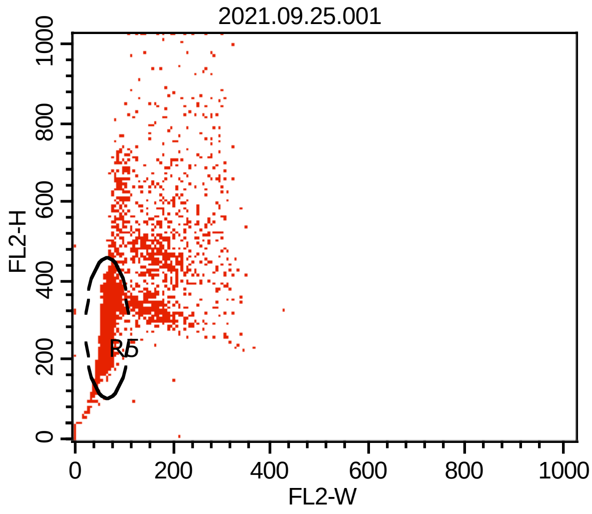

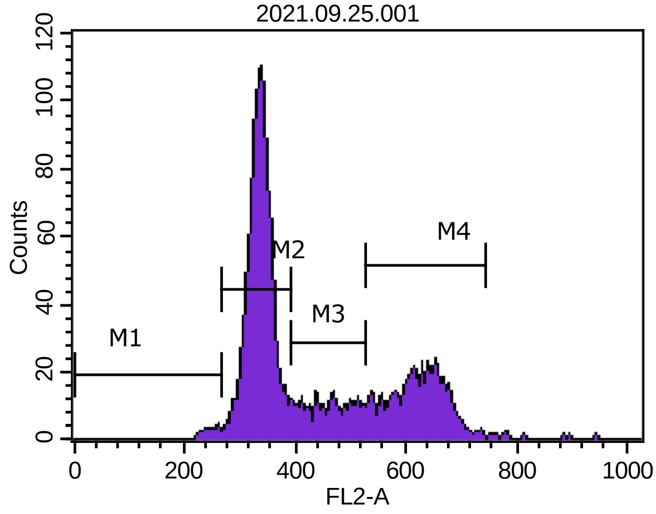

File: 2021.09.25.001 Sample ID: MDA231 Ctr I

Marker	% Gated
All	100.00
M1	0.70
M2	61.43
M3	12.96
M4	24.88

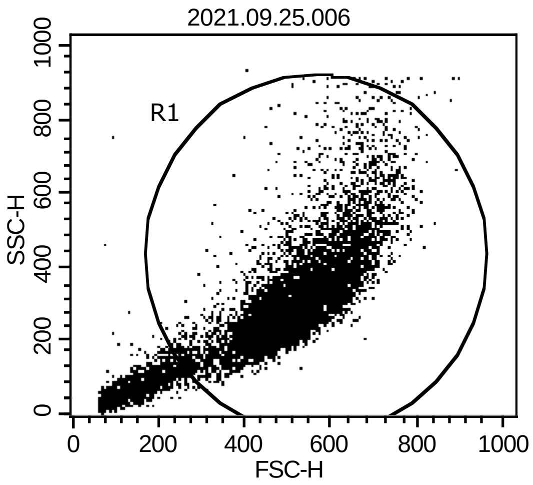

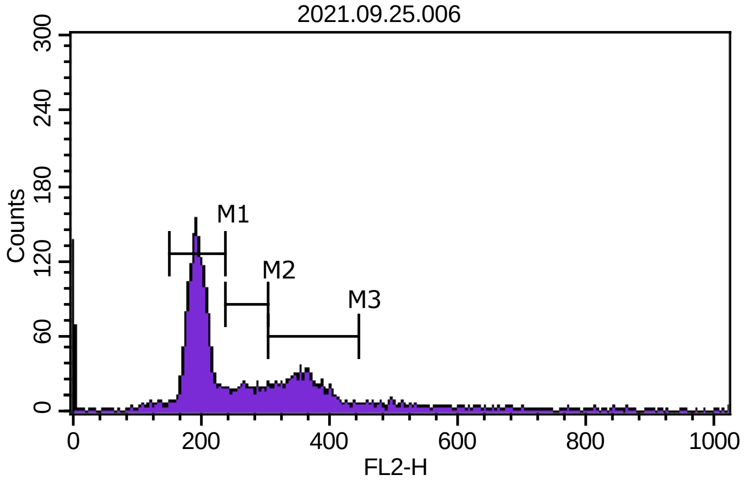

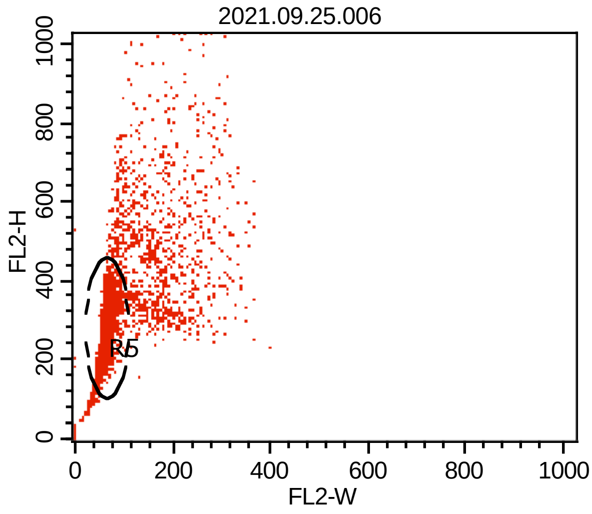

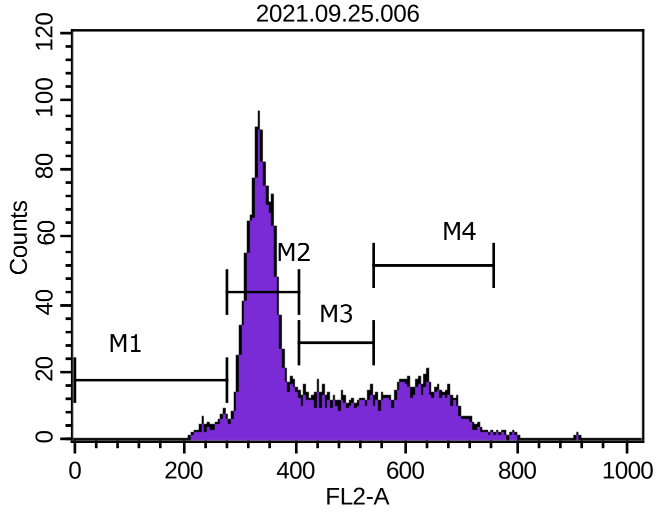

File: 2021.09.25.006 Sample ID: MDA231 PAX IC50 II

Marker	% Gated
All	100.00
M1	1.98
M2	61.52
M3	14.46
M4	22.07

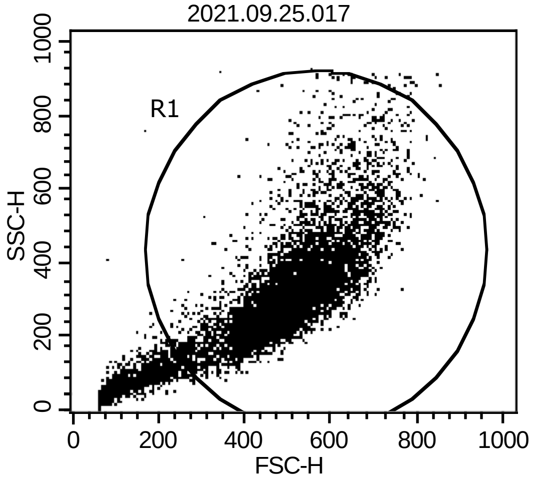

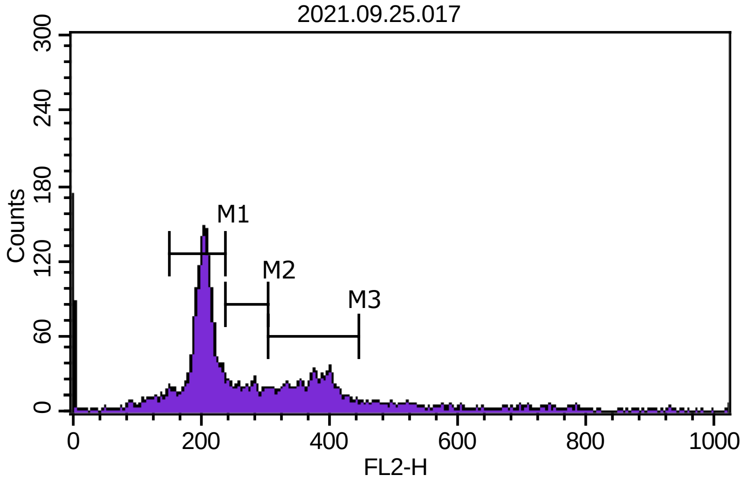

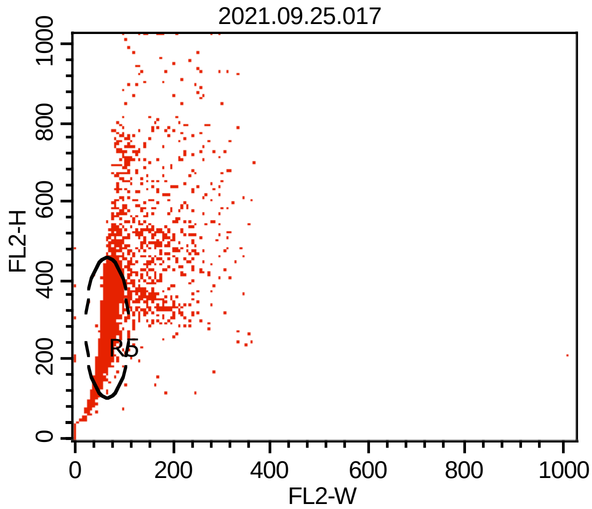

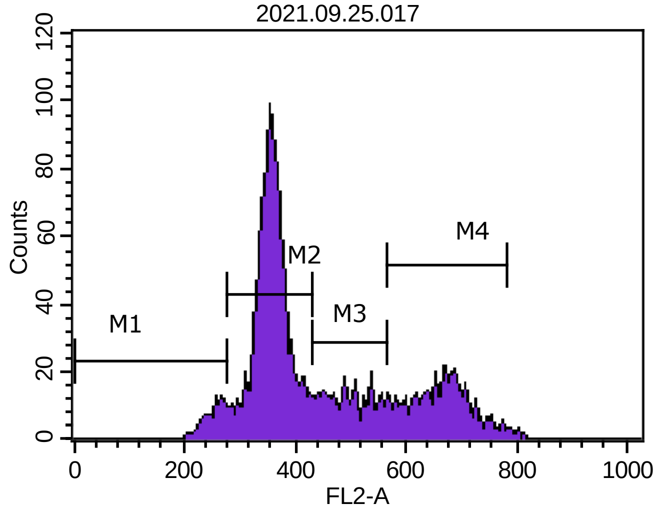

File: 2021.09.25.017 Sample ID: MDA231 PAX 2IC50 I

Marker	% Gated
All	100.00
M1	3.87
M2	60.46
M3	14.39
M4	21.52

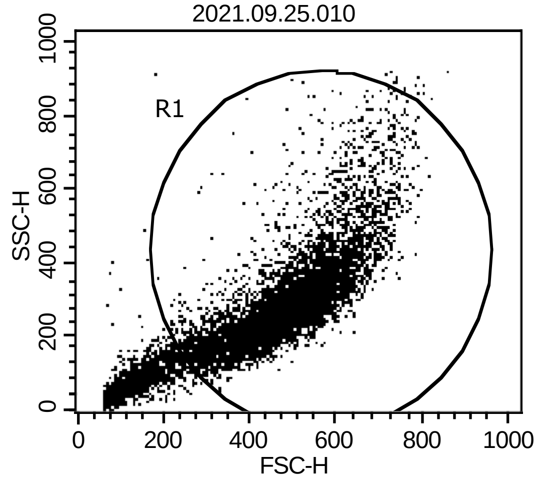

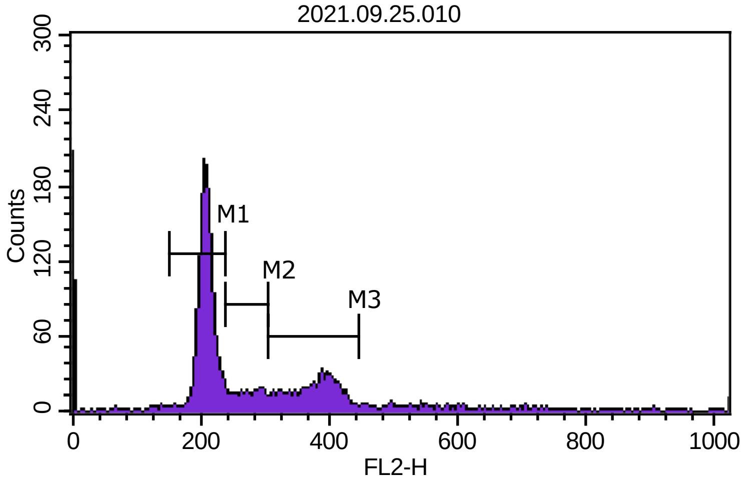

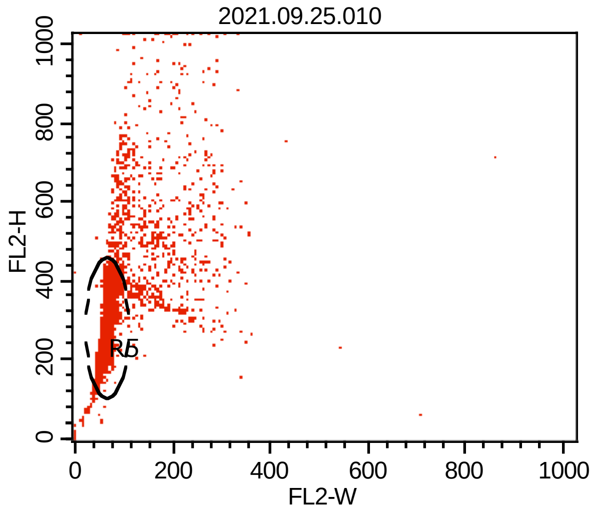

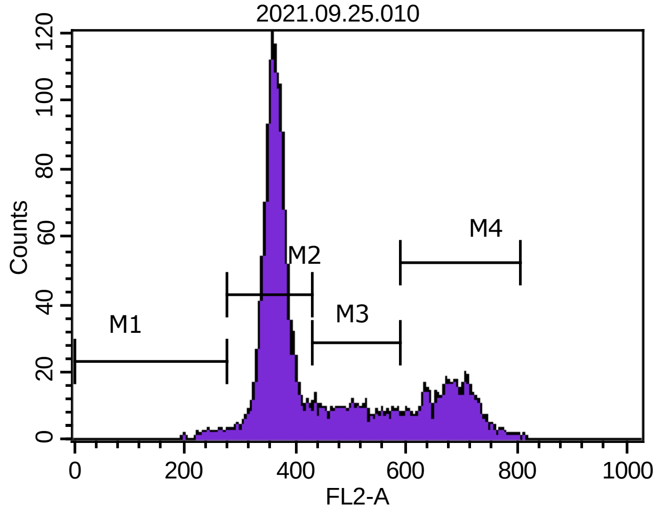

File: 2021.09.25.010 Sample ID: MDA231 CAM IC50 II

Marker	% Gated
All	100.00
M1	1.00
M2	66.69
M3	13.47
M4	18.98

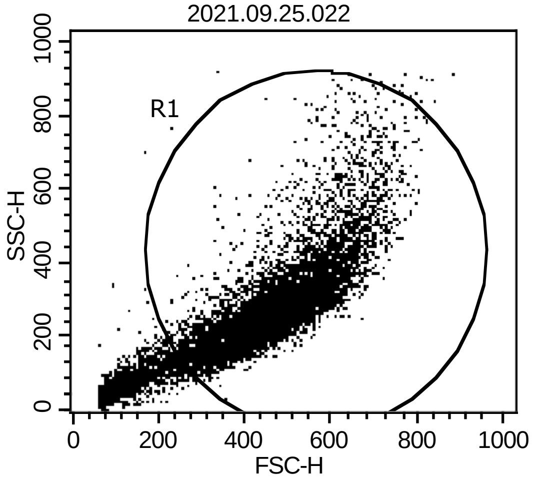

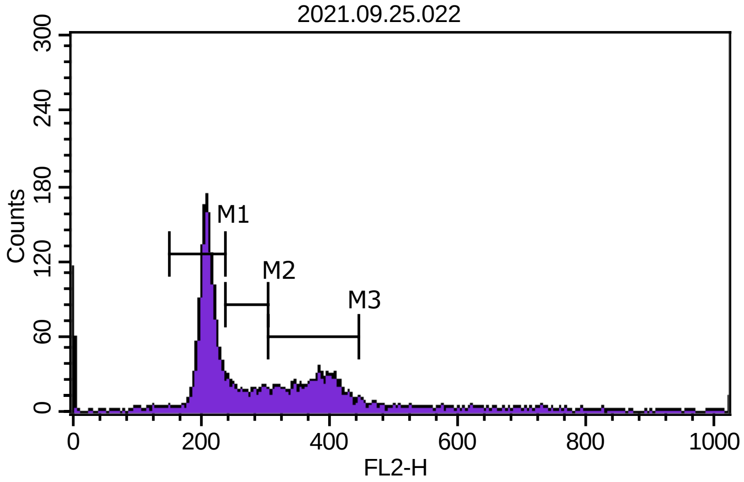

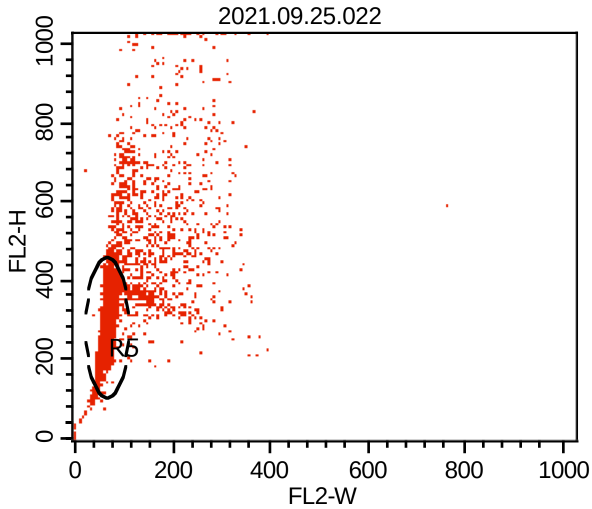

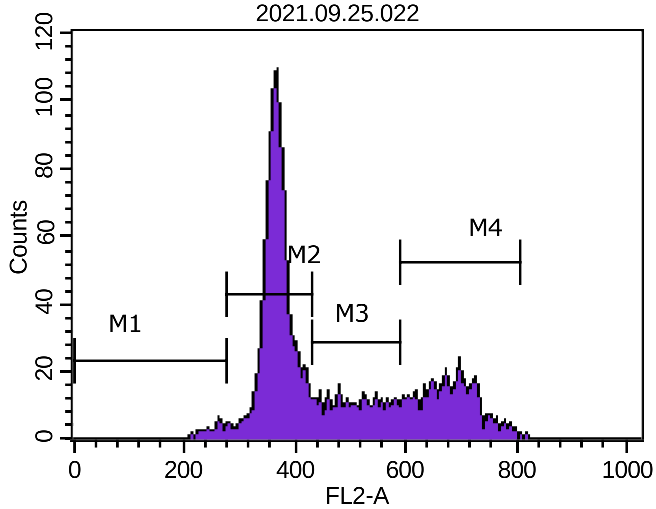

File: 2021.09.25.022 Sample ID: MDA231 CAM 2IC50 II

Marker	% Gated
All	100.00
M1	1.08
M2	60.30
M3	16.70
M4	22.23

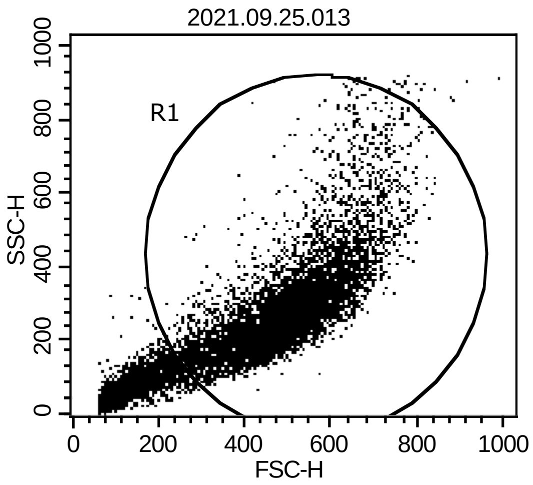

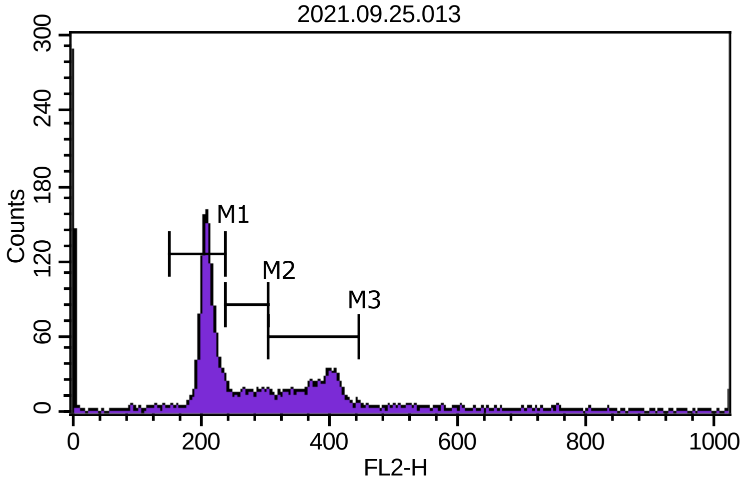

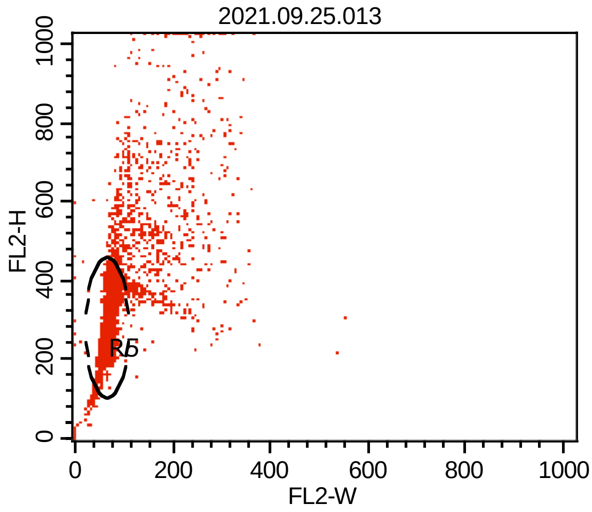

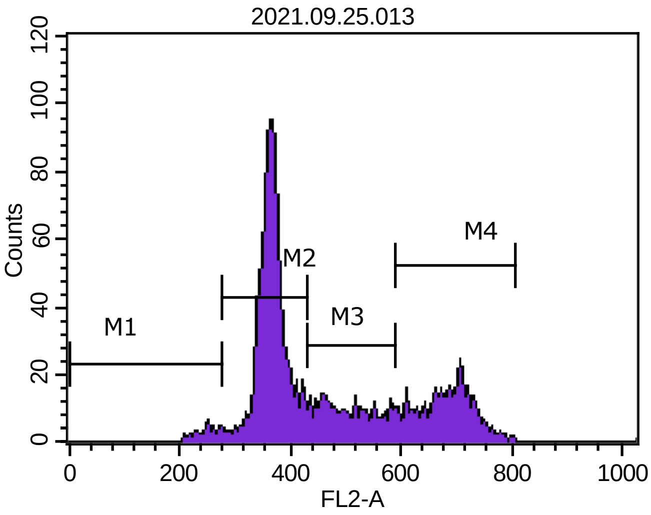

File: 2021.09.25.013 Sample ID: MDA231 MIX IC50 I

Marker	% Gated
All	100.00
M1	1.62
M2	59.22
M3	17.09
M4	22.25

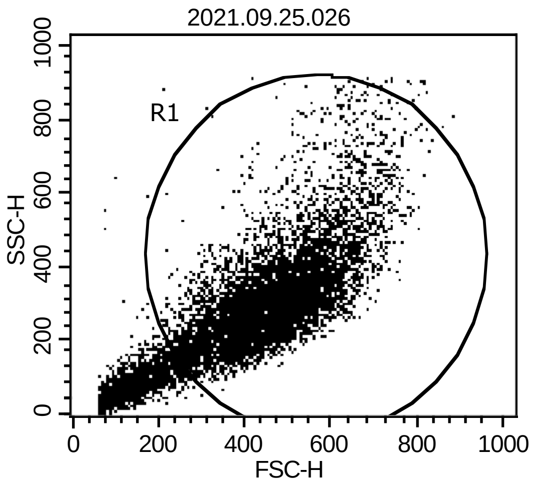

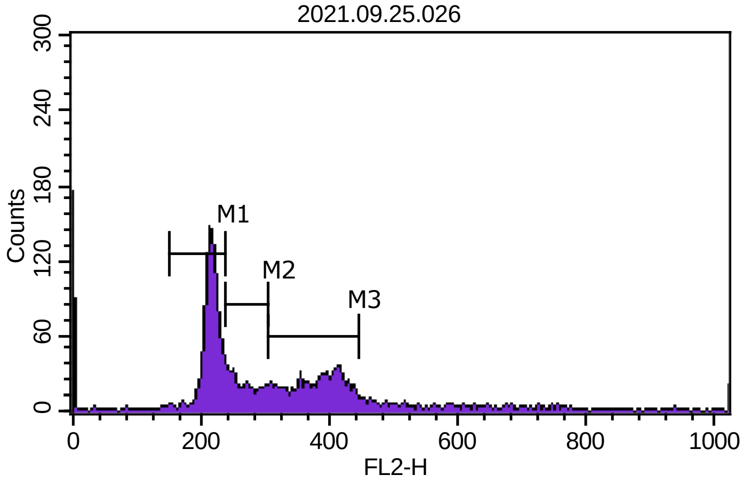

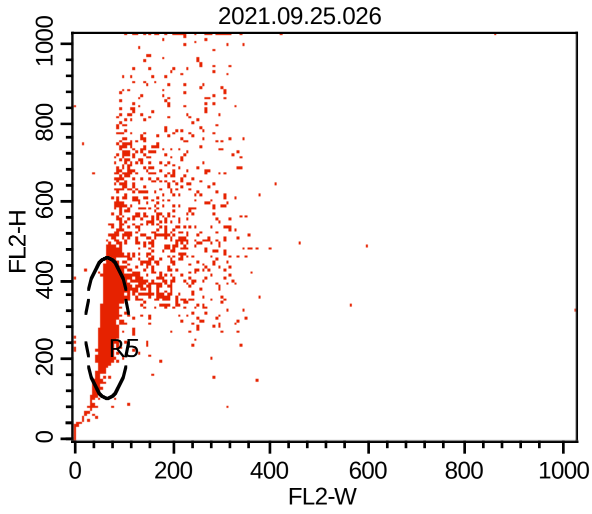

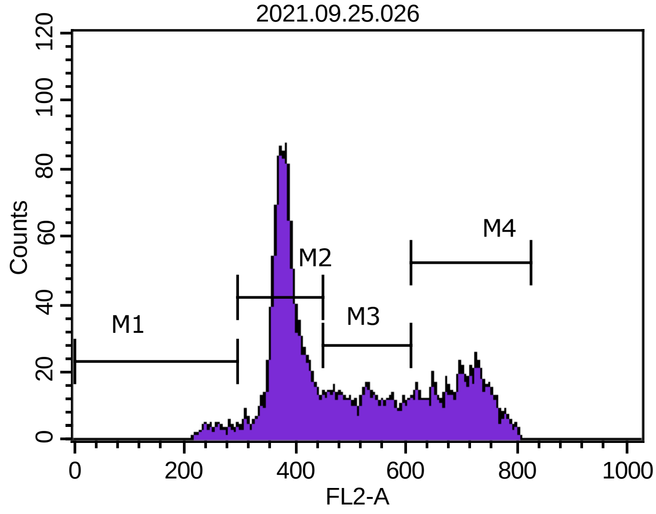

File: 2021.09.25.026   Sample ID: MDA231 MIX 2IC50 II

Marker	% Gated
All	100.00
M1	1.60
M2	54.23
M3	19.59
M4	24.85
